# From *Escherichia coli* mutant ^13^C labeling data to a core kinetic model: A kinetic model parameterization pipeline

**DOI:** 10.1371/journal.pcbi.1007319

**Published:** 2019-09-10

**Authors:** Charles J. Foster, Saratram Gopalakrishnan, Maciek R. Antoniewicz, Costas D. Maranas

**Affiliations:** 1 Department of Chemical Engineering, The Pennsylvania State University, University Park, Pennsylvania, United States of America; 2 Department of Chemical and Biomolecular Engineering, University of Delaware. Newark, Delaware, United States of America; CPERI, GREECE

## Abstract

Kinetic models of metabolic networks offer the promise of quantitative phenotype prediction. The mechanistic characterization of enzyme catalyzed reactions allows for tracing the effect of perturbations in metabolite concentrations and reaction fluxes in response to genetic and environmental perturbation that are beyond the scope of stoichiometric models. In this study, we develop a two-step computational pipeline for the rapid parameterization of kinetic models of metabolic networks using a curated metabolic model and available ^13^C-labeling distributions under multiple genetic and environmental perturbations. The first step involves the elucidation of all intracellular fluxes in a core model of *E*. *coli* containing 74 reactions and 61 metabolites using ^13^C-Metabolic Flux Analysis (^13^C-MFA). Here, fluxes corresponding to the mid-exponential growth phase are elucidated for seven single gene deletion mutants from upper glycolysis, pentose phosphate pathway and the Entner-Doudoroff pathway. The computed flux ranges are then used to parameterize the same (i.e., k-ecoli74) core kinetic model for *E*. *coli* with 55 substrate-level regulations using the newly developed K-FIT parameterization algorithm. The K-FIT algorithm employs a combination of equation decomposition and iterative solution techniques to evaluate steady-state fluxes in response to genetic perturbations. k-ecoli74 predicted 86% of flux values for strains used during fitting within a single standard deviation of ^13^C-MFA estimated values. By performing both tasks using the same network, errors associated with lack of congruity between the two networks are avoided, allowing for seamless integration of data with model building. Product yield predictions and comparison with previously developed kinetic models indicate shifts in flux ranges and the presence or absence of mutant strains delivering flux towards pathways of interest from training data significantly impact predictive capabilities. Using this workflow, the impact of completeness of fluxomic datasets and the importance of specific genetic perturbations on uncertainties in kinetic parameter estimation are evaluated.

## Author summary

Microbial production hosts are used for production of a wide range of commodity chemicals. Improving the conversion efficiency of microbial strains is critical to the economic viability and the continued push towards the use of environmentally neutral bioprocesses as a means for producing the chemicals society depends on. Metabolic models have played a key role in helping us to predict metabolic behavior in response to environmental and genetic perturbation that can maximize efficiency. Recently, kinetic models of metabolism have re-emerged as a means for characterizing metabolism, offering improvements over their stoichiometric counterparts in both the type of information that can be gleaned from them, and in prediction accuracy. Despite recent developments, a lack of raw experimental data needed for flux elucidation and, subsequently, kinetic parameterization, and high computation cost have prevented the development of a uniform workflow for construction of the most informative kinetic models. Here, we have incorporated raw ^13^C-isotopic labeling data and a computationally inexpensive parameterization algorithm into a kinetic parameterization pipeline to ensure that the resulting kinetic model (k-ecoli74) conforms to experimental data. We show how the use of an identical metabolic network for flux elucidation and kinetic parameterization influences predictive capabilities.

## Introduction

### Background

The standardization and automation of genome characterization and editing techniques has been accompanied by a rapid increase in the number of prokaryotic and eukaryotic microbial organisms available for engineering for overproduction of target commodity metabolites. With annotated genomes and CRISPR-Cas toolboxes consolidated in organism-specific biofoundries [1–5], the demand for biologically robust genetic intervention strategies for target metabolite overproduction has also increased. This has created a need for a standardized computational workflow capable of reliably predicting phenotype based on genetic intervention strategies. Traditional stoichiometric models of metabolic networks and integer programming design algorithms such as OptKnock [6] have provided insight into metabolic state as a function of genetic perturbation. These tools provide information on how an organism may behave under a specific genetic condition. However, the types of information that can be gleaned from them is limited to what can be deduced from reaction flux distributions, and flux ranges predicted via stoichiometry-based models are generally broad and subject to variability based on a user-defined cellular objective (i.e. maximum butyric acid production [7], maximum biomass [8], MOMA [9]). In recent years kinetic models of metabolism have (re)-emerged as a promising modeling paradigm offering a number of advantages over their stoichiometric counterparts albeit with a much higher effort associated with their construction. Kinetic models incorporate the mechanistic details of enzyme catalyzed reactions in metabolic networks to characterize a metabolite concentration/reaction flux pair as a function of physiological state. Kinetic models developed to date have primarily focused on characterizing either metabolic pathway behavior [10–13] or core metabolic function [14–17], as the computational burden and data needs associated with parameter estimation has been a limiting factor in both the rate of kinetic model development and scale-up of metabolic network. A number of kinetic formalisms and parameterization methods have been used to characterize and predict the dynamic behavior of metabolic systems. Mass action [18–20], S-system [21, 22], and log-transformed kinetic [10, 23–25] models have used canonical kinetic rate expressions to describe enzyme-catalyzed reactions. A number of models have also used mechanistic or approximate mechanistic expressions to characterize behavior of metabolic pathways [11–13, 26] and central carbon metabolism [14, 16, 17]. Both gradient-based [27–29] and stochastic [14, 30, 31] optimization methods have been developed for *in silico* identification of optimal sets of kinetic parameters. However, probabilistic [12] and meta-heuristic [30] parameterization methods have been at the forefront of recent kinetic model development [12, 14–16, 32] to bypass the computational challenges arising from the nonconvexity of the constraints and interdependence of kinetic parameters. Off the shelf solvers are ill-equipped to address the kinetic parametrization problem for these reasons, as the non-linearity of algebraic equality constraints required to ensure conservation of mass makes finding even an initial feasible point challenging. Furthermore, evaluation of mutant strain metabolite concentration, enzyme level, and reaction flux requires integration of a system of ordinary differential equations that tends to be stiff and prone to failure. The ensemble modeling paradigm [30] was introduced to address these challenges, and incorporated mechanistic rate expressions. However, application of this method to large metabolic networks requires very significant computational resources for parametrization rendering follow-up analysis of parameter robustness and sensitivity analysis prohibitive. This is due to the costly integration step needed each time a new steady-state is evaluated and the many thousands of recombination operations needed for convergence due to the non-inclusion of gradient information to guide the search. Greene et al. [33] have demonstrated how conservation and stability analysis on kinetic models in an ensemble can significantly improve parameterization time in the ensemble modeling paradigm by reducing both the number of model evaluations required to parameterize a kinetic model and the time required for a single model evaluation. Their complete methodology, however, has been limited in application to toy networks and a core kinetic model. Lee et al. [34] have used first order partial derivatives with the ensemble modeling paradigm to characterize the robustness of synthetic metabolic pathways by perturbing Michaelis-Menten (*K*_*m*_) and maximum rate of reaction (*Vmax*) parameters across all models in an ensemble and determining the probability of failure. Their analysis, however, was limited to systems with less than 20 reactions and did not require experimental training data.

Kinetic model development has been further hindered by a lack of experimental datasets to use in parametrization. Fluxomic data (in the case of ensemble modeling paradigm) across a range of single or multiple gene knockout conditions, and coverage across the entire metabolic network considered in a kinetic model, is required to generate a set of kinetic parameters capable of predicting metabolic state for any given condition. Mechanistic microbial kinetic models have been developed for core [14, 17] and genome-wide [32] metabolism of *E*. *coli* as well as core metabolism for *C*. *thermocellum* [16], while canonical models have been shown to be scalable to genome scale size [18].

A prominent tool for characterizing reaction flux distribution in living cells is ^13^C metabolic flux analysis (^13^C-MFA) [35–41]. The workflow for ^13^C-MFA is carried out in experimental and computational stages. The experimental stage is performed by first introducing an isotopically labeled substrate to a growing cell culture. Then the labeling distribution of mass isotopmers of labeled metabolites produced by the cell is measured using gas chromatography-mass spectrometry [42], liquid chromatography-mass spectrometry [43], or nuclear magnetic resonance spectroscopy [44]. Proteinogenic amino acid fragments and metabolites from central carbon metabolism are prominently featured in isotopic labeling datasets [42, 43, 45]. The ^13^C-MFA computational workflow consists of a least-squares fitting problem, whereby a metabolic flux distribution is estimated by minimizing the variance weighted sum of squared residuals (SSR) between the experimentally measured isotopic labeling distribution and a predicted isotopic labeling distribution inferred via the estimated flux distribution [46]. Application of ^13^C-MFA has yielded quantitative core metabolic characterization of a plethora of prokaryotic and eukaryotic organisms and cell types [37, 47–52]. It has also shed light on flux redirection under genetically and environmentally perturbed conditions [53–55] and revealed previously unknown pathway activity usage [56, 57]. Elucidation of atom mappings for peripheral carbon pathways and more elegant methods for mapping carbon flow (*i*.*e*. the EMU framework [58]) has allowed for scale-up of ^13^C-MFA to the genome-scale in three organism: *E*. *coli* [40], *Synechocystis PCC 6803* [59], and *Synechococcus PCC 7923* [60].

### Scope of work

In order to accelerate the emergence of kinetic metabolic models as a viable tool for use in microbial strain design, we have developed a pipeline for rapid kinetic parameterization. By coupling ^13^C-MFA and kinetic parameterization computational methods using the same metabolic network, we acknowledge the intrinsic dependence of kinetic modeling on the metabolic network and ^13^C-glucose labeling datasets used to elucidate the flux distributions required for kinetic parametrization. We also provide a customizable framework for generating kinetic models that are consistent with reported flux ranges and applicable to any microbial metabolic network for which a set of isotopic labeling data across multiple genetic or environmental conditions can be procured. Our workflow for rapidly generating kinetic models of metabolic networks was carried out in two phases: flux elucidation was carried out via ^13^C-MFA and kinetic model parameterization using the gradient-based K-FIT algorithm developed by Gopalakrishnan et al. [61].

K-FIT differs from previously developed elementary decomposition approaches to kinetic parameterization by optimizing the model on the space of wild-type enzyme fractions and reverse elementary fluxes. Net fluxes and concentrations for the mutant networks are then recovered based on an iterative decomposition approach. The inclusion of gradient information in K- FIT also allows for the direct assessment kinetic parameter sensitivities. Taking advantage of ^13^C labeling data available for *E*. *coli* generated using glucose feedstock labeled at the first two carbon positions ([1,2-^13^C]glucose, known to yield precise flux estimations in *E*. *coli* core metabolism by ^13^C-MFA when compared to other single-tracer experiments [62]), we have applied our seamless workflow to the development of a kinetic model of *E*. *coli* core metabolism. Our model can predict metabolite pool size and metabolic flux distribution, satisfies flux distributions for wild-type and seven single gene deletion mutants from upper glycolysis, PP pathway, and Entner-Doudoroff (ED) pathway under mid-exponential growth conditions, and recapitulates carbon uptake kinetics.

We elucidated flux distributions and 95% confidence ranges for wild-type and seven single gene deletion mutant strains of *E*. *coli (Δfbp*, *Δedd*, *Δeda*, *Δpgi*, *Δrpe*, *Δzwf*, and *Δgnd*). For strains *Δfbp*, *Δedd*, and *Δeda*, the flux distributions were similar to the wild-type strain, with statistically insignificant variations from the wild-type strain in glucose uptake rate. Strains *Δpgi*, *Δrpe*, *Δzwf* (glucose-6-phosphate dehydrogenase (G6PDH2r) knock-out), and *Δgnd* each exhibited flux redirections compared to the wild-type strain. Given the obtained flux datasets, we then parameterized a core kinetic model using a metabolic network identical to that used for flux elucidation with 74 reactions, 61 metabolites, and 55 substrate-level inhibitions. Although activation is a prevalent regulatory mechanism in metabolism [63], it was not included in the model due to the absence of complete cofactor balance and known inaccuracy of energy metabolism representation in core metabolism flux distributions. The use of identical metabolic networks for both flux elucidation and kinetic parameterization safeguards against information loss in the form of feasible flux distributions due to flux projection from a core model to a larger model stemming from incomplete atom mapping and stoichiometric information.

Kinetic parameterization time was reduced by approximately 80% over the ensemble modeling (EM) method employed by Khodayari et al. [14] from more than a week to 36 hours (real time, due to evaluation of locally unstable parameter sets) for a core kinetic model. The average parameterization time per random initialization during k-ecoli74 parameterization was approximately four hours. The model constructed in this study (k-ecoli74) predicted 86% of reaction fluxes within a single standard deviation (SD), 95% within two SDs, and 99% within three SDs of ^13^C-MFA estimated flux values for mutant strains used in fitting.

k-ecoli74 was validated, and its predictive capabilities tested by comparing product yields for seven metabolites produced by nine engineered strains with experimental yield values and those reported for the previously developed k-ecoli457 kinetic model [32]. k-ecoli74 predicted product yields well overall, significantly outperforming the predictive capabilities of k-ecoli457 for malate and acetate production by engineered strains. This was due to similarity in experimental conditions between the strain engineering studies and those used for ^13^C-labeling data generation in this study (i.e. glucose-rich batch culture, mid-exponential growth phase). Metabolites not included in k-ecoli74 were systematically overpredicted due to the use of central carbon metabolism drains as proxies for pathways not included in the model. For example, 2,3-butanediol was over-predicted due to the use of pyruvate dehydrogenase (PDH) as a proxy for the heterologous 2,3-butanediol synthesis pathway (not included in k-ecoli74).

When flux data generated using a simplified metabolic network was used to parameterize a kinetic model with a metabolic network identical to k-ecoli74, discrepancies in flux predictions were observed in amino acid metabolism. In particular, there were significant differences in both the magnitude and directionality of reactions in serine, glycine, threonine, and glutamate metabolism. A significant decrease in predictive capability was observed when the kinetic model parameterized using simplified flux dataset was used to predict metabolite yields for the nine validation strains. The model generated with the reduced flux set was only able to predict feasible flux distributions for five of nine validation strains tested, and four out of those five strains yielded predictions similar to or worse than k-ecoli74 predictions.

The method developed in this study provides a framework for constructing kinetic models of metabolic networks from experimental data that ensures all pathways with resolvable flux ranges are accounted for in parameter estimation, and carbon and energy balance are characterized as accurately as possible. In addition, the relative computational tractability of the kinetic parameterization method used in this approach allows for the *a posteriori* analyses on kinetic parameter identifiability and sensitivity. Application of kinetic parameterization pipeline developed in this study to any organism or metabolic network requires a set of ^13^C labeling data, an identical metabolic network for flux elucidation and kinetic parameterization, a ^13^C-MFA software package for flux elucidation, and the K-FIT algorithm for kinetic parametrization.

## Materials and methods

### Kinetic parameterization pipeline

**Fig 1:**
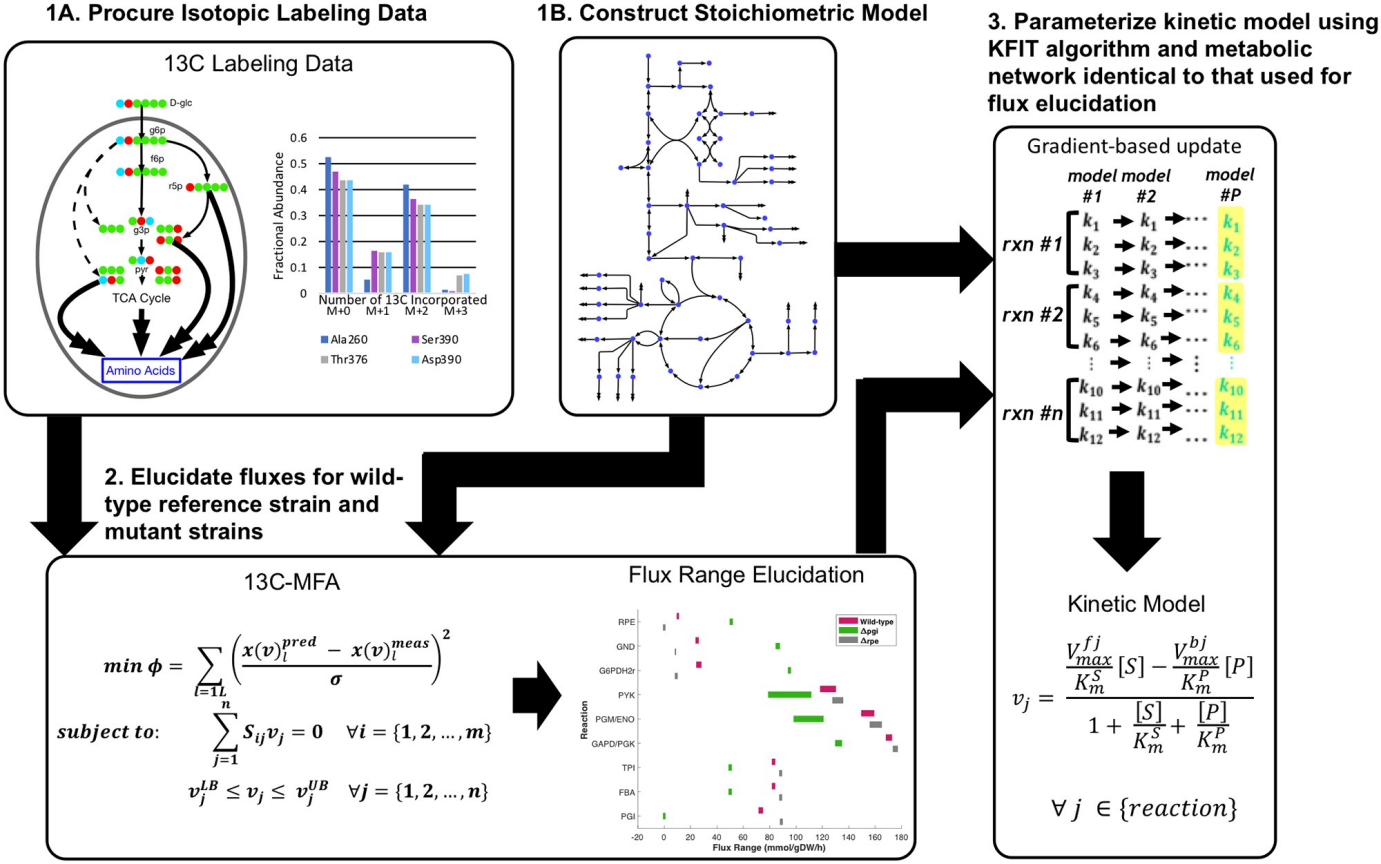
Pictorial representation of the kinetic parameterization pipeline for constructing kinetic models of metabolic networks. (1A): A set of isotopic labeling data across a range of genetic and/or environmental conditions must be generated or procured. (1B): A stoichiometric model must be constructed. (2): ^13^C-MFA is performed, and flux ranges are elucidated using the procured isotopic labeling data across all strains from step 1A and the stoichiometric model constructed in step 1B. (3): The flux distributions that were generated in step 2 are used as training data for parameterizing the kinetic model using the stoichiometric model constructed in step 1B and the K-FIT algorithm.

The developed workflow for kinetic model construction relies on identical metabolic networks for flux elucidation and kinetic parameterization. This circumvents the information loss in the form of feasible solutions associated with the projection of the core model flux distributions onto the genome scale metabolic model and allows for the seamless integration of biomass yield information on precursor pathway drains. A pictorial representation of the kinetic parameterization pipeline is presented in Fig 1. The steps for constructing a kinetic model using the pipeline are as follows: first the stoichiometric model (Fig 1, step 1B) and corresponding atom mapping model are assembled (Fig 1, step 1A). Then they are used for flux elucidation of wild-type and genetic mutant strains of the organism of interest via ^13^C-MFA from ^13^C-isotopic labeling data (Fig 1, step 2). Finally, using the constructed stoichiometric model, the elucidated flux ranges, and any substrate level regulatory events identified in literature or inferred via computational methods (*e*.*g*. SIMMER [41] or model-based identification [64]), the kinetic model is parameterized using the K-FIT kinetic parameterization algorithm (Fig 1, step 3).

### Model coverage

**Fig 2:**
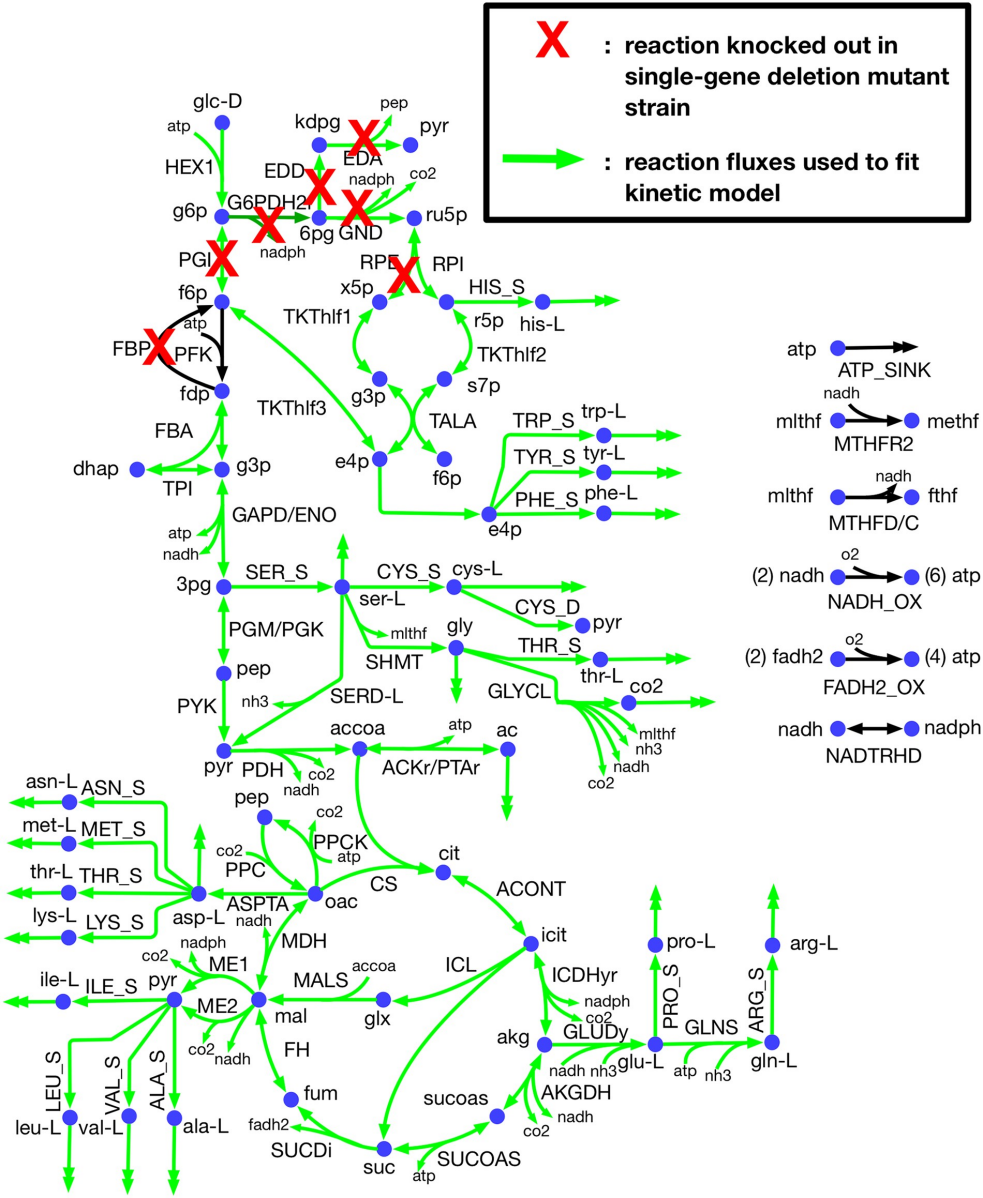
k-ecoli74 metabolic network. Reaction and metabolite abbreviations provided in S4 File.

**Fig 3:**
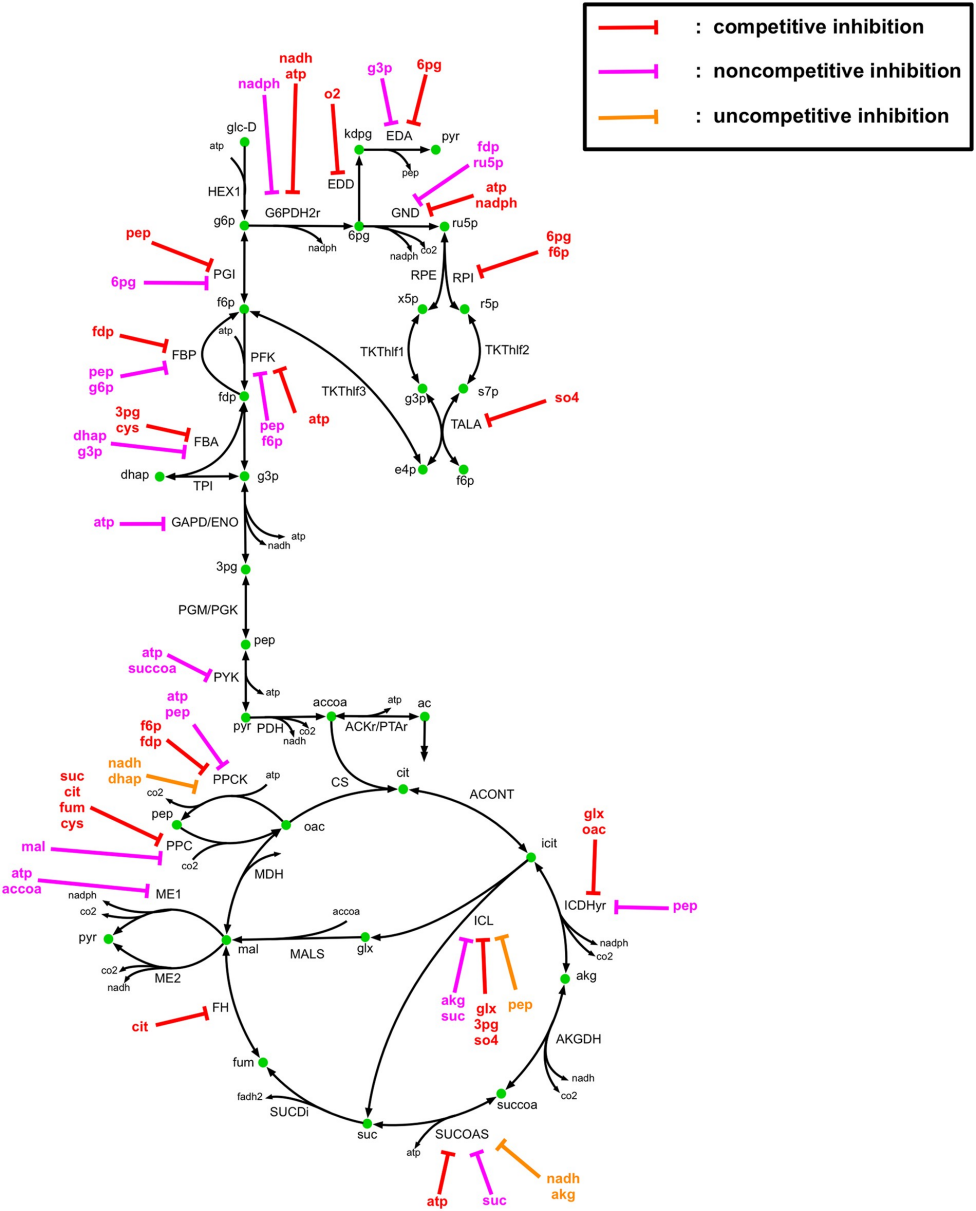
k-ecoli74 regulatory network. Reaction and metabolite abbreviations provided in S4 File.

The core metabolic network used for ^13^C-MFA in this study (Fig 2) contains 74 reactions and 61 metabolites. The metabolic network and atom mapping model developed by Leighty and Antoniewicz [42] was used as a basis with the addition of L-serine deaminase (SERD-L). Pyruvate kinase (PYK) was also allowed to carry reverse flux to account for the significant flux converting pyruvate to phosphoenolpyruvate by the terminal phosphotransferase in the PTS system observed in vivo [65] and phosphenolpyruvate synthase activity. Atom transitions for SERD-L were taken from the imEco726 genome-scale atom mapping model [40]. The network included glycolytic, pentose phosphate (PP) pathway, and tricarboxylic acid (TCA) cycle pathways, as well as anaplerotic and cataplerotic reactions, lumped amino acid synthesis pathways, glycine cleavage, energy metabolism, acetate metabolism, and a biomass sink reaction. The metabolic network used for kinetic parameterization included identical reactions to those used for ^13^C-MFA. However, the biomass sink reaction was decomposed into individual metabolite sinks for each biomass precursor. A total of 55 substrate level regulations on central carbon metabolism reactions curated from the BRENDA [66] and EcoCyc [67] databases were included in the kinetic model, and are depicted in Fig 3. Substrate level regulations included competitive, uncompetitive, and noncompetitive inhibition. The reactions, metabolites, allosteric regulations, and atom mapping model used in this study are provided in S4 File.

### Experimental data

^13^C isotopic labeling datasets for wild-type and seven single gene deletion mutant strains with glucose feedstock labeled at the first two carbon positions (100% [1,2-^13^C] glucose) generated by Long and Antoniewicz [68] was used as input data for the kinetic parameterization pipeline. Mass isotopomer distributions for 22 metabolite fragments derived from 10 amino acids (alanine, glycine, valine, leucine, serine, threonine, phenylalanine, aspartate, glutamate, tyrosine) and two sugar phosphates (ribose 5-phosphate, glucose 6-phosphate) were included in each labeling dataset. The seven mutant strains with available ^13^C isotopic labeling data included *pgi*, *fbp*, *zwf* (glucose-6-phosphate dehydrogenase (G6PDH2r) knock-out), *gnd*, *rpe*, *edd*, and *eda* knockout strains. Fig 2 shows the location of reactions in upper glycolysis, PP pathway, and ED pathway inactivated by genetic knockouts in strains use for parameterization.

Metabolite yield data from a series of genetically engineered overproducing strains was procured from literature [69–78] and used for model validation and testing predictive capabilities under conditions not included in the training data. Model validation strains included both up and downregulation of central carbon metabolism reactions as well as genetic knockouts. Genetic perturbation strategies, metabolites whose yields were tested, and experimental yield values are listed in S9 File, and a visual representation of perturbation strategies are provided in Fig D in S3 File. Strains designed to overproduce malate, acetate, L-valine, naringenin, lactic acid, 2,3-butanediol, and glucaric acid were included in the validation set. The malate overproduction strain was characterized by a downregulation of phosphotransacetylase (PTAr) and upregulation of phosphoenolpyruvate carboxylase (PPC). The acetate overproduction strain was characterized by a downregulation of ribose-5-phosphate isomerase (RPI). Two naringenin overproducing strains were considered, one characterized by succinyl-CoA synthetase (SUCOAS) knockout and fumarase (FUM) downregulation, and the other malate dehydrogenase (MDH) knockout and SUCOAS downregulation. Two lactic acid overproduction strains were also included, one characterized by acetate kinase (ACKr) downregulation and the other by ACKr knockout. One 2,3-butanediol overproduction strain was characterized by PYK overexpression, and one glucaric acid overproduction strain was characterized by NAD transhydrogenase (NADTRHD) overexpression.

### ^13^C-MFA

Flux elucidation and 95% confidence interval estimation was performed for wild-type and each of the seven mutant strains using [1,2-^13^C]glucose isotope tracer data. The atom mapping model assembled from the atom transitions gleaned from Leighty and Antoniewicz [42] and the imEco726 model [40] was used to construct the elementary metabolite unit (EMU) network. An EMU is a subset of carbon atoms of any metabolite included in the stoichiometric model, and the EMU network characterizes how these subsets of carbon travel through the reactions in the network. The EMU network allows for characterization of the mass isotopmoer distribution of each metabolite in the metabolic network based on the isotope labeling scheme of the substrate upon estimation of a steady-state flux distribution [58]. Strain-specific biomass composition and acetate yields determined by Long et al. [79] were used for flux fitting. EMU decomposition, flux elucidation, and confidence interval estimation were performed according to the procedure outlined by Gopalakrishnan and Maranas [40], and glucose uptake was normalized to 100 flux units as a basis for each fitting. A summary of the ^13^C-MFA computational procedure is provided in S1 File. In order to ensure the best flux distribution was selected for use in the kinetic parameterization procedure, 100 randomly initialized multi-starts were performed for each strain. The minimized ^13^C-MFA objective was the variance weighted SSR between experimentally measured mass isotopomer distributions and mass isotopomer distributions inferred using the EMU network and steady-state flux distribution. A solution was accepted only if the algorithm converged to that solution at least 50% of the time. This does not guarantee convergence to the true global minimum but provides a practical safeguard against accepting local minima as solutions.

### Kinetic parameterization

Kinetic parameterization was performed using the flux distributions estimated via ^13^C-MFA for wild-type and all mutant strains as training data. The gradient-based K-FIT algorithm [61] was used to parameterize the kinetic model. The wild-type flux distribution was used to estimate a set of elementary kinetic parameters (i.e. a set of kinetic parameters satisfying the wild-type flux distribution was generated). This was done to ensure the set of elementary parameters corresponds to a feasible steady-state solution in the wild-type strain. The elementary kinetic parameters were then used to estimate mutant flux distributions, and calculate the variance weighted sum of squared residual error (SSR) between all ^13^C-MFA mutant flux distributions and mutant flux distributions predicted using the estimated elementary parameters. Kinetic parameters were updated using gradient-based optimization, and the process was repeated until a local minimum was reached. To ensure reactions carrying little flux with narrow flux ranges were not over-weighted in the objective function, standard deviation used for weighting of residual errors was defined as the maximum value of either 1.0, five percent of the corresponding flux value, or the standard deviation value calculated according to the ^13^C-MFA 95% confidence interval. A summary of the K-FIT optimization algorithm is included in S1 File. Due to the nonconvexity of the resultant optimization model, 500 randomly-initialized multi-starts were performed. A threshold for change in concentration of any metabolite with respect to time was set at 10^−6^ flux unit to ensure strict adherence to the pseudo-steady state assumption [80]. All fluxes used as training data were scaled by the ratio of absolute mutant glucose uptake rate to absolute wild-type glucose uptake rate.

Model acceptance criteria was based on the SSR value, flux distribution reproducibility, and ability to predict flux distributions for genetic conditions not used for parameterization. In order for a model to be selected as a best model, two criteria had to be satisfied: first, the model had to yield the lowest SSR with at least one other model yielding a local minimum SSR value within 10% of the best model’s value (to ensure a reproducible solution). Because elementary kinetic parameters are the product of two distinct optimization variables (reverse elementary reaction flux and enzyme complex fractional abundance), local minima with similar SSR values but different elementary parameters may exist. Selecting a model with SSR similar to other local minima (i.e. within 10% of optimal SSR value) allows for the assessment of the sensitivity of *K*_*m*_ and *Vmax* parameters using models yielding similar flux distributions through comparison of parameters generated by different models with similar fitness to data. The second condition for model acceptance was the capability to estimate steady-state flux distributions under genetic conditions not used for parameterization [81].

#### Standard deviation of elementary kinetic parameters

First, the covariance matrix was calculated from the Hessian matrix of flux residuals (discrepancies) with respect to elementary kinetic parameters (model parameters). The Hessian matrix (***H***) was calculated using numerical approximation by first assessing the Jacobian matrix ***J*** (i.e. the partial derivatives) of the residual vector with respect to elementary kinetic parameters ($\frac{dr}{dk}$, where ***r*** is the vector of all residuals in the objective function and ***k*** is the vector of all elementary kinetic parameters in the model). From ***J***, ***H*** was approximated (**H**~**J**′*W*J, where ***W*** is a matrix whose diagonal element are the weighting of all residuals in the objective function). The variance of each elementary parameter corresponds to the diagonal elements of the covariance matrix (covariance is defined as ***H***^−1^) [82]. From the variance vector, the approximated standard deviation SD of each elementary kinetic parameter was directly assessed as ($SD=\sqrt{Variance}$) [82]. Sensitivity was then assessed by computing the coefficient of variation (*CV*(%) = 100*SD/*Converged Parameter value*) [83].

#### Michaelis-Menten parameter and maximum rate of reaction derivation

Michaelis-Menten parameter (*K*_*m*_) and maximum rate of reaction (*Vmax*) ranges were assembled from the optimal elementary rate constants. A generalized algorithm for deriving *K*_*m*_ and *Vmax* terms from elementary kinetic parameters using the King-Altman method [84] was developed, accounting for competitive, uncompetitive, and noncompetitive inhibition. For each enzyme complex participating in a given reaction, the algorithm first traces each possible path leading to its formation. Then elementary kinetic rate constants that account for the formation and consumption of inhibitory complexes for that enzyme are incorporated into each expression. The resulting expressions are then arranged into Michaelis-Menten form by constructing a rate law characterizing the change in concentration of the final product released in the reaction with respect to time. A total of 1,246 *K*_*m*_ and 100 *Vmax* parameters were considered, corresponding to those parameters for which metabolomic data required for scaling of elementary kinetic parameters was available. Because of the complexity of Michaelis-Menten expressions derived using the King-Altman method, each kinetic term in the denominator of each Michaelis-Menten expression was considered a distinct *K*_*m*_ parameter.

Wild-type *E*. *coli* metabolomics data was gleaned from Park et al. [85] due to the similarity of the growth conditions. Lumped parameter ranges were identified by assessing *K*_*m*_ and *Vmax* values using metabolite concentrations either one standard deviation above or one standard deviation below the mean concentration value to scale elementary kinetic parameters. Unscaled elementary kinetic parameter ranges were defined as one standard deviation above and one standard deviation below the average parameter value in each model. Range expansion of *K*_*m*_ and *Vmax* parameters (and thus resolvability of *K*_*m*_ and *Vmax* parameters) was determined by assessing the total range expansion of each *K*_*m*_ and *Vmax* parameter when the spanned values in all models within 10% of the optimal SSR were considered.

#### Model validation under genetic conditions not included in training data

A series of genetic perturbation tests were performed to validate k-ecoli74, explore its predictive capabilities, and compare the results with those of previously developed kinetic models. The model’s ability to predict experimental product yields under conditions not used for parameterization was tested for nine engineered strains targeting overproduction of seven products with available experimental yield data [69–77] in a method consistent with that employed by Khodayari *et al*. [32, 86]. Only strains whose entire set of perturbed reactions were included in the kinetic model and whose metabolite of interest, or in the case of metabolites outside of central carbon metabolism and lactic acid, whose direct precursors were included in the model, were considered for comparison. The location of genetic perturbations in the validation strains were different from those used for training the model. Whereas genetic interventions in training data were confined to upper glycolysis, the ED pathway, and the pentose phosphate pathway, intervention strategies in model validation strains were primarily from lower glycolysis, the TCA cycle, or pathways leading to metabolic byproduct formation. The only validation strain with a genetic intervention strategy targeting a reaction in either upper glycolysis or the pentose phosphate pathway was the acetate overproduction strain, which was characterized by a downregulation of RPI.

## Results

### Kinetic model training data recapitulation

**Fig 4:**
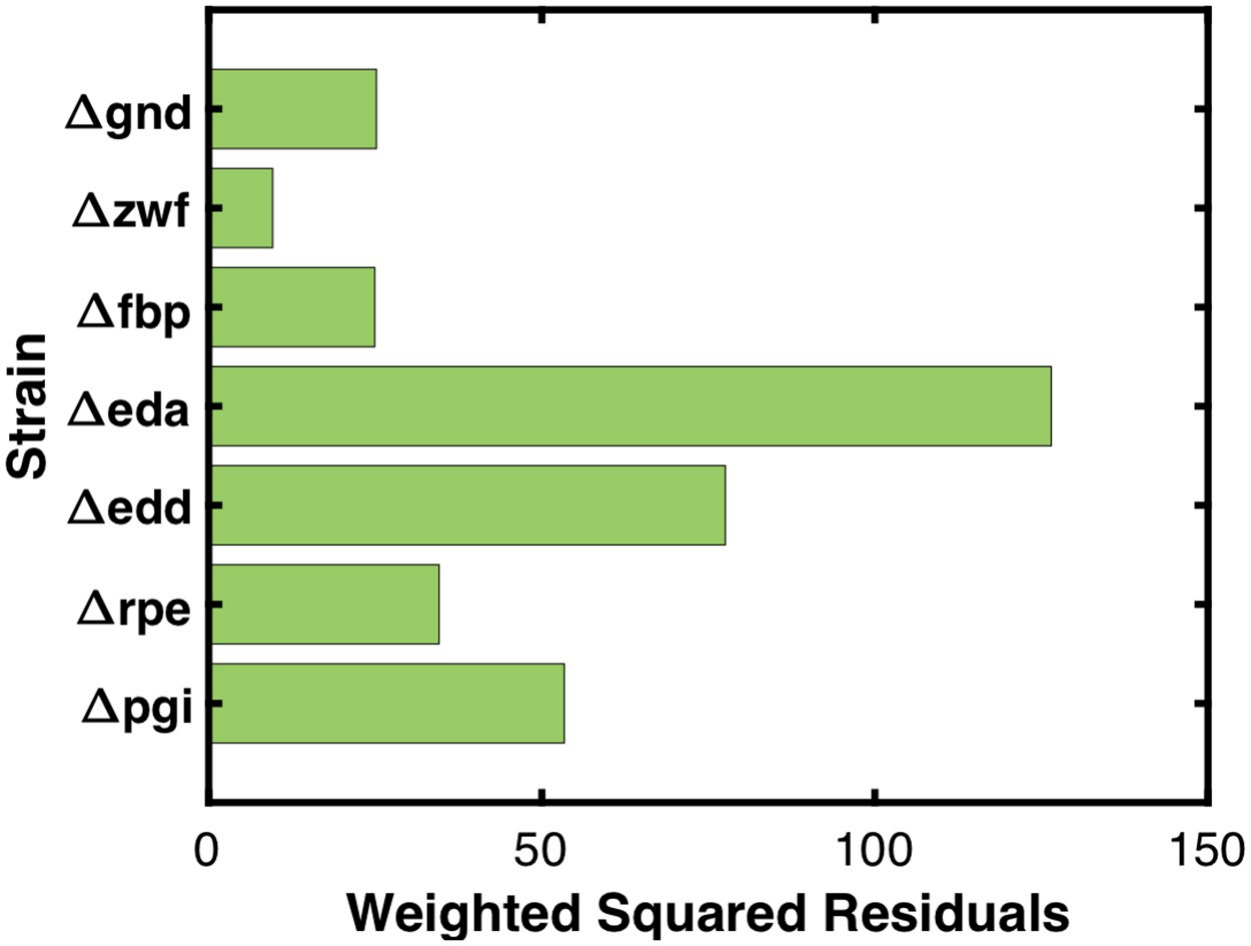
k-ecoli74 fitness to mutant flux distributions used for kinetic parameterization.

**Fig 5:**
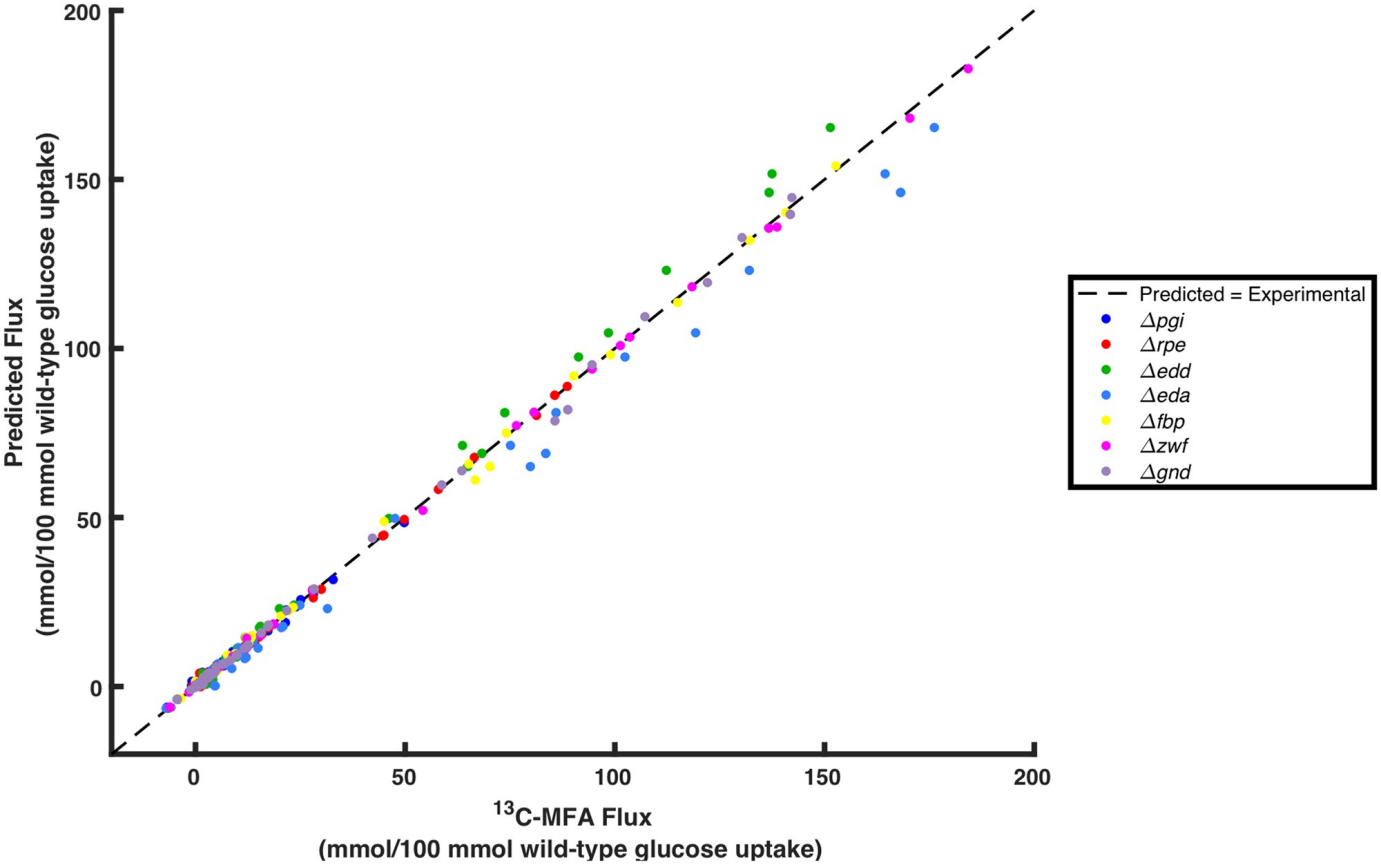
Comparison of k-ecoli74-predicted flux values with ^13^C-MFA flux values.

**Fig 6:**
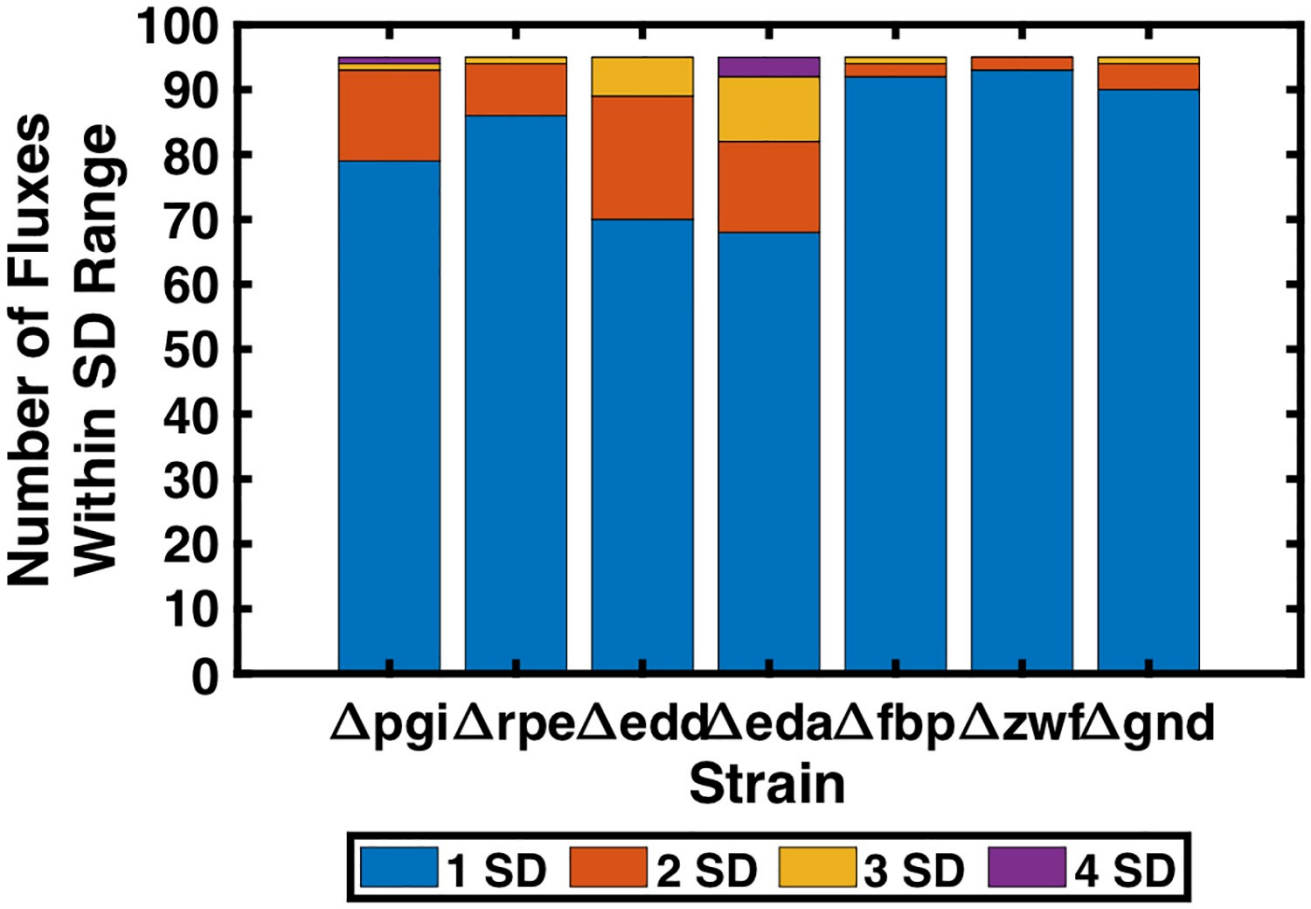
Number of k-ecoli74-predicted fluxes from each strain used for parameterization falling with one, two, three, or four SDs of the corresponding ^13^C-MFA value.

A total of 896 elementary kinetic parameters and 78 inhibitor constants were estimated corresponding to the 74 reactions, 34 biomass precursor sink reactions, and 55 substrate-level regulations in the metabolic network. Fluxes corresponding to central carbon metabolism, amino acid synthesis and degradation, and biomass formation were fitted. Fructose bisphosphatase (FBP) and phosphofructokinase (PFK) fluxes were excluded from fitting due to unresolvability (i.e., very wide ranges) stemming from simplifications made to energy metabolism in the core model. Energy metabolism and nutrient uptake reactions were also disregarded in the fitting due to simplifications and unavoidable inaccuracy of energy metabolism fluxes due to the nature of core metabolism ^13^C-MFA [40]. A total of 94 fluxes were fitted per mutant strain. The best-fitting model across seven mutant strains that also exhibited model stability across all strains for which metabolite yields were predicted had a SSR of 338 and an average weighted squared residual per mutant reaction flux of 0.52 (SSR was calculated for the seven single gene deletion strains used for training. The nine non-inclusion strains used to validate the model and evaluate predictive capability were not included in the calculation of SSR or evaluation of model fitness). Two additional models at neighboring local minima yielded SSR values within 10% of the optimal SSR value. The average percent error for reactions whose SD was within 20% of the experimental flux value (210 of 665 reactions) was 5.7%. Of those reactions whose SD was greater than 20% of the experimental flux value (455 of 665 reactions), 91% of predicted values differed from the corresponding experimental value by less than 1 mmol/100 mmol wild-type glucose uptake, and the average deviation was 0.36 mmol/100 mmol wild-type glucose uptake. The contribution of each strain to overall lack of fitness is shown in Fig 4. *Δeda* was the worst fitting strain, and contributed 36% of the overall SSR, while *Δedd* contributed to 23% of the overall SSR. *Δpgi* contributed to 19% of the total SSR, and no other strain contributed more than 8% of the total SSR. The best fitting strain was *Δzwf*, and contributed only 2% of overall SSR. Fig 5 shows a comparison of model-predicted flux values and ^13^C-MFA-estimated flux values. The plotted data yielded a Pearson correlation coefficient of 0.997, indicating a strong positive correlation between model predictions and ^13^C-MFA values. No single flux in the metabolic network deviated from the ^13^C-MFA value by more than a single SD for more than three fitted strains. Five predicted fluxes deviated from ^13^C-MFA values by more than a single SD across three strains. Lower glycolytic reaction PDH deviated by more than a single SD in *Δpgi*, *Δedd*, and *Δeda*. Fig E in S3 File depicts the relative contribution of each reaction in each strain to the total SSR. Acetate exchange, deviated by more than a single SD in *Δpgi*, *Δeda*, and *Δgnd*, and TCA cycle reactions citrate synthase (CS) and aconitase (ACONT) deviated by more than a single SD in *Δedd*, *Δeda*, and *Δfbp*, while isocitrate dehydrogenase (ICDHyr) and alpha ketoglutarate dehydrogenase (AKGDH), deviated by more than a single SD in *Δpgi*, *Δedd*, and *Δeda*. Fig 6 shows the number of fluxes falling within one, two, three, or four SDs of ^13^C-MFA values across strains. The results indicate 86% of all fluxes fitted fell within a single SD, 96% fell within two SD, and 99% within three SDs of their corresponding ^13^C-MFA values.

Glycolytic reaction fluxes were underpredicted in *Δpgi* due to the misdirection of flux towards the biomass sink reaction for ribose 5-phosphate and through glycine cleavage (GLYCL) instead of through L-serine deaminase (SERD-L). k-ecoli74 predicted *Δpgi* succinyl-CoA synthetase (SUCOAS) flux in the opposite direction of the ^13^C-MFA flux. Because SUCOAS flux was in the positive direction in all other strains used for parameterization, the fitted kinetic parameters were incapable of delivering reverse flux through SUCOAS. Serine hydroxymethyltransferase (SHMT) and GLYCL flux were also overpredicted by more than one SD in *Δpgi*. k-ecoli74 underpredicted SERD-L flux, while serine synthesis flux was predicted higher than ^13^C-MFA flux to satisfy biomass precursor demand. GLYCL, therefore, served as a sink for carbon that should have been delivered back to glycolysis. Most *Δpgi* PP and ED pathway fluxes deviated from ^13^C-MFA values by less than a single SD. k-ecoli74 predicted a 98% reduction in atp concentration and a 76% reduction in nadh concentration (competitive inhibitors of G6PDH2r) in *Δpgi* relative to the wild-type strain. This caused an increase in enzyme available to catalyze the G6PDH2r reaction, and k-ecoli74 was, therefore, able to successfully re-direct the entirety of carbon flux through the PP pathway. Acetate exchange was a significant carbon sink in all strains except for *Δpgi*. The optimal set of kinetic parameters, therefore, were suited for delivering significant flux towards acetate secretion, and that flux was overpredicted in the *Δpgi* strain.

Due to ED pathway irreversibility, the effect of *Δeda* and *Δedd* knockouts on the predicted flux distributions were almost identical, despite having different ^13^C-MFA flux distributions when scaled by absolute glucose uptake rate. In *Δedd*, reactions whose predicted fluxes were greater than a single SD from the corresponding ^13^C-MFA value (and contributed most to SSR) were primarily found in glycolysis and the TCA cycle. Fluxes were overpredicted in both pathways. *Δedd* glucose uptake rate when scaled to 100 mmol of wild-type glucose uptake was 8.7 mmol/100 mmol wild-type glucose uptake lower than wild-type, thus fluxes were overpredicted. In the *Δeda* strain, TCA cycle reactions, acetate formation and excretion, and PDH contributed most to SSR. Scaled *Δeda* glucose uptake rate was 2.4 mmol/100 mmol wild-type glucose uptake greater than in the wild-type strain. Despite their differences in glucose uptake rates from the wild-type strain and each other, glucose uptake rate did not contribute significantly to SSR in either strain compared to the aforementioned reactions. This is because standard deviations for glucose uptake rate in both strains was large, ensuring glucose uptake was not a primary source of error.

In *Δfbp*, only TCA cycle predicted fluxes deviated from ^13^C-MFA values by more than a single SD. Specifically, upper TCA cycle fluxes were overpredicted by the model. Because CS flux was increased, ACONT flux was increased as well. Isocitrate lyase (ICL) showed increased activity compared to the experimental data, redirecting carbon flowing through the TCA cycle. Glutamate dehydrogenase (GLUDy) also had a higher predicted flux than the ^13^C-MFA value in order to drain the excess carbon. While FBP and PFK reactions were not used to fit the kinetic model because of their unresolvability across all other strains during ^13^C-MFA, leaving them out of the fitting did not have an impact on the resulting fitness of the *Δfbp* strain. FBP flux was fixed to zero in *Δfbp*, and the flux through PFK was constrained by the other reactions in the network producing and consuming f6p (glucose-6-phosphate isomerase (PGI), transaldolase (TALA), fructose bisphosphate aldolase (FBA)) to ensure conservation of mass. Because flux through these reactions fit the data well in *Δfbp*, so did PFK flux. This was confirmed upon comparison of PFK flux with ^13^C-MFA value (PFK residual error in the *Δfbp* strain was 0.2).

In *Δrpe*, *Δzwf*, and *Δgnd*, no more than one predicted flux deviated from ^13^C-MFA values by more than a single SD. In *Δrpe*, arginine synthesis flux was underpredicted, and was the only non-biomass reactions with predicted fluxes outside of a single SD from the ^13^C-MFA value. No predicted *Δzwf* fluxes deviated from the ^13^C-MFA flux values by more than a single SD. In *Δgnd*, only acetate exchange deviated from the ^13^C-MFA flux values by more than a single SD.

**Fig 7:**
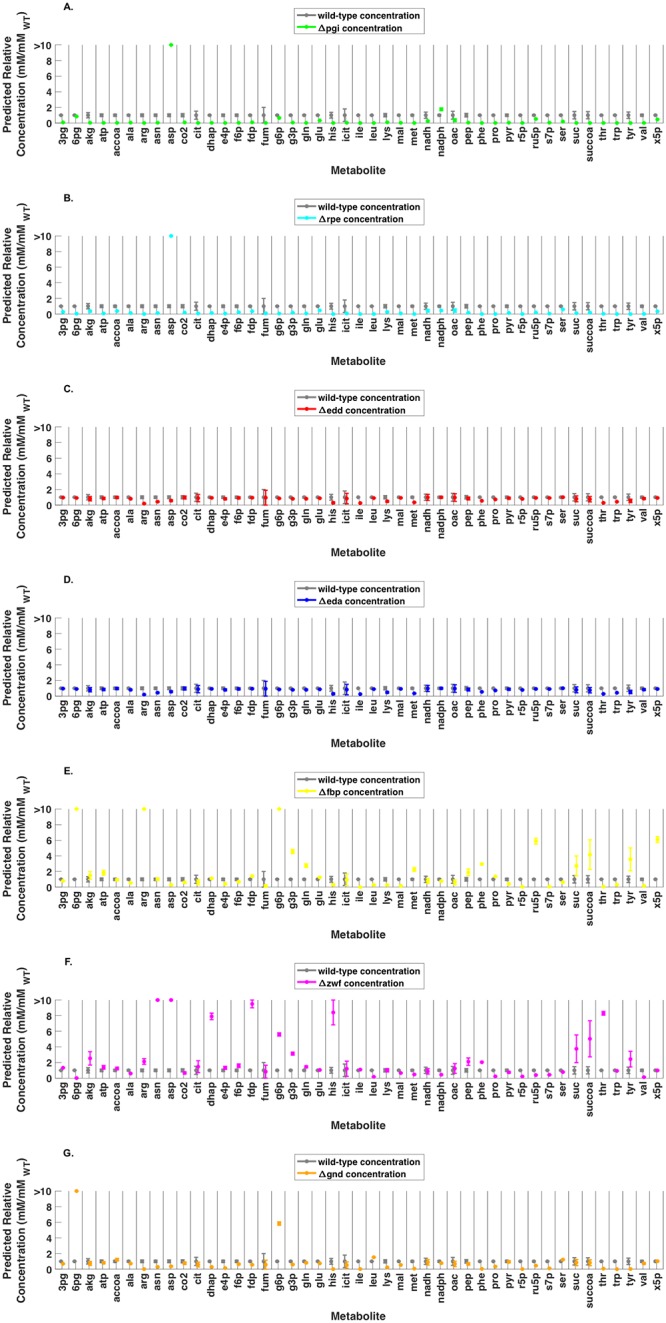
Comparison of mutant strain predicted scaled metabolite concentrations with wild-type metabolite concentration (all values scaled by wild-type absolute metabolite concentration). (A) *Δpgi* relative concentration (B) *Δrpe* relative concentration (C) *Δeda* relative concentration (D) *Δedd* relative concentration (E) *Δfbp* relative concentration (F) *Δzwf* relative concentration (G) *Δgnd* relative concentration. Error bars denote range of a single standard deviation from mean scaled concentration value.

Changes in metabolite concentration due to genetic perturbation were also assessed. The scaled metabolite concentrations for all metabolites with corresponding wild-type experimental data across each mutant strain used for parameterization are compared to wild-type metabolite concentrations in Fig 7. Significant changes in concentration were observed across all strains except *Δedd*. The only notable pool size changes in *Δedd* was a general decrease in amino acid concentrations. In *Δpgi*, a significant decrease in metabolite pool sizes for most metabolites across the metabolic networks was observed. The only metabolites with increased pool sizes were aspartate (>1000%) and nadph (+76%). *Δrpe* also exhibited significant decreases in pool size for most metabolites in the network. The only increase in concentration relative to the wild-type strain was for aspartate (>1000%). In *Δeda*, 2-dehydro-3-deoxy-D-gluconate 6-phosphate (kdpg) concentration increased significantly (>1000%), and there was a general decrease in amino acid concentrations. In *Δfbp*, glycolytic intermediates glucose-6-phosphate (g6p) (>1000%) and glyceraldehyde-3-phosphate (g3p) (+350%) and PP pathway intermediates 6-phospho-D-gluconate (6pg) (>1000%), D-ribulose-5-phosphate (ru5p) (+486%), and D-xylulose-5-phosphate (x5p) (+506%) increased. A redistribution of amino acid pool sizes was also observed: aspartate (-72%), histidine (-72%), isoleucine (-99%), leucine (-73%), lysine (-73%), threonine (-94%), tryptophan (-71%), and valine (-83%) concentrations all decreased significantly, while arginine (>1000%), glutamine (+174%), methionine (+117%), phenylalanine (+190%), and tyrosine (+249%) concentration increased. In *Δzwf*, a general increase in the pool size of aspartate and aspartate-derived amino acids and a decrease in pyruvate and pyruvate derived amino acid pool sizes was observed. Histidine (>1000%) concentration also increased, as well as the pool sizes for glycolytic intermediates dihydroxyacetone phosphate (dhap) (688%), fructose 1,6-bisphosphate (fdp) (875%), g6p (457%), and g3p (212%). In *Δgnd*, both g6p (481%) and 6pg (+933%) concentrations increased significantly, causing ED pathway activity to increase compared to wild-type and all other mutant strains. PP pathway intermediates erythrose-4-phosphate (e4p) (-86%) and sedoheptulose-7-phsophate (s7p) (-90%) pools sizes decreased, and a general decrease in amino acid pool sizes was observed.

While the majority of metabolite concentration values estimated by k-ecoli74 fell within ranges consistent with experimental data [85], the loose constraints placed on optimization variables (enzyme complex fractional abundance (1*e^−3^ < [*e*] < 1) and reverse reaction flux (0 < *v*_*r*_ < 10000)) allowed elementary kinetic parameters to assume a broad range of values (0 < *k* < 1000000). As a result, mutant strain metabolite fold changes also had the potential to assume large values upon steady-state evaluation that were not always physiologically relevant. This occured when the magnitudes of the estimated forward and reverse elementary kinetic parameters for any elementary step in the network were different by several orders of magnitude. The fold changes predicted for aspartate in the *Δpgi*, *Δrpe*, and *Δzwf* strain reflected this, as the elementary parameters for production and consumption of aspartate by ASPTA differ by several orders of magnitude. Small ASPTA free enzyme fractional abundance in those strains relative to other enzyme complexes ensured aspartate concentration had to be large to recapitulate flux. Another reason for large metabolite concentrations was to help to characterize flux redirections in mutant strains. In *Δgnd*, 6pg and kdpg concentrations were increased to values >1000% of the wild-type concentration to help characterize the increased ED pathway flux that was dependent upon their concentrations, and only observed in the *Δgnd* strain. In *Δpgi*, extracellular glucose concentration was increased because glycolysis was blocked and growth rate was low, causing the model to predict an accumulation of glucose. In other instances, large metabolite concentrations were an artifact of the assumptions made in the modeling framework. kdpg was only produced and consumed in the ED pathway, and was not an inhibitor of any reactions. As a result, in the *Δedd* and *Δeda* knockout strains, because only EDD or EDA enzyme level was forced to zero, kdpg was able to assume large values as long as the net flux through the reaction that was not knocked out in the pathway was zero. These predictions are a limitation of the elementary decomposition approach for kinetic parameterization in the absence of metabolomics training data, as the mathematical framework of K-FIT allows for flexibility in metabolite concentration predictions when only fluxes are fitted.

### Parameterization with reduced flux dataset

To test how using the kinetic parameterization pipeline changes training data recapitulation and impacts kinetic model predictive capabilities, we parameterized a kinetic model with metabolic network coverage identical to k-ecoli74, but using flux data for wild-type and the seven mutant strains of *E*. *coli* generated with a reduced metabolic network. Our first attempt at parameterizing a kinetic model using a reduced network was with a set of fluxes generated using the minimal metabolic network used by Ishii et al. [36]. K-FIT, however, was unable to converge to a solution because simplifications to the Ishii et al. network and biomass equation when compared to k-ecoli74 were too drastic. Flux redirections upon flux projection associated with differences in the biomass formulation caused the wild-type and mutant strain flux distributions to fall outside of the nullspace of the k-ecoli74 stoichiometric matrix, and the gradient-based search tended to go towards unstable parameter sets.

The reduced network used for flux elucidation, therefore, was a network simplified from the k-ecoli74 metabolic network (see S4 File). It did not contain amino acid degradation pathways, and amino acid synthesis pathways were simplified to reflect only the transfer of carbon from central carbon metabolites to the amino acids being synthesized. All metabolism that did not contribute to carbon mapping (nitrogen, oxygen, and sulfur metabolism, energy metabolism and cofactors) was also removed from the metabolic network. Flux elucidation with these network simplifications using the same labeling data resulted in 95% confidence intervals that did not contain a steady-state flux distribution for the k-ecoli74 network upon flux projection. Flux projection was, therefore, performed by minimizing the violation of 95% confidence intervals of the reduced network by the wild-type flux distribution at metabolic steady-state. Differences between the projected flux distribution and the reduced network 95% confidence intervals were confined to amino acid synthesis and degradation pathways. The minimum total violation of reduced network 95% confidence intervals in the wild-type strain was 53 mmol/100 mmol glucose uptake, and the most notable rearrangement was in GLUDy flux (as GLUDy carried only the flux towards glutamate required for biomass formation). Serine, and glycine metabolism also underwent shifts in fluxes (due to the absence of ammonium from those pathways in the reduced network). SSR for each flux elucidation and the kinetic parameterization are provided in Table 1. The kinetic model parameterized using the reduced flux dataset that yielded the best SSR out of 30 multi-starts, had an SSR of 559, and an average SSR per data point of 2.5. An increase in SSR across conditions was observed, and is expected due to simplifications in the metabolic network used for flux elucidation. While 30 parameterizations converged to local minima, over 500 parameterizations were initialized. K-FIT encountered many unstable models during parameterization, but a limited number of initializations were able to converge, which was an improvement over parameterization using flux data generated with the Ishii et al. [36] network.

**Table 1:** SSR value and degrees of freedom (DOF) from ^13^C-MFA flux elucidation for wild-type and 7 single gene deletion mutant strains using reduced model, SSR value for K-FIT kinetic parameterization using reduced model elucidated fluxes as training data.

Strain	DOF	SSR
wild-type	68	77.9
Δpgi	70	37.2
Δrpe	40	46.4
Δeda	52	92.9
Δedd	69	93.8
Δzwf	70	83.5
Δfbp	68	69.9
Δgnd	69	190.0
K-FIT Parameterization	-	559

Predictions by the model parameterized with the reduced network flux dataset were inconsistent with k-ecoli74 predictions for several reactions peripheral to central carbon metabolism. Table 2 highlights the significant inconsistencies between fluxes estimated using the kinetic model parameterized using the reduced network flux dataset and k-ecoli74. We included only central carbon metabolism reactions with ^13^C-MFA-elucidated fluxes in reduced network mutant training datasets. When compared to the flux ranges predicted by k-ecoli74, the flux ranges predicted by the core model parameterized with reduced flux dataset were consistent across glycolysis, PP pathway, and TCA cycle. However, due to deviation from k-ecoli74 training data observed in the wild-type strain upon flux projection, both the directionality and magnitude of amino acid synthesis and degradation reactions was impacted across all strains used for parameterization. In the projected wild-type flux distribution, reverse flux through SERD-L was required to generate a flux distribution at metabolic steady state with minimum violation of the bounds defined using the reduced network 95% confidence intervals. As a result, SERD-L flux was predicted to be reversed across all strains used for parameterization. In *Δzwf*, *Δgnd*, and *Δrpe*, the magnitude of SERD-L flux was so large that the lumped serine synthesis reaction was also predicted to carry reverse flux. GLYCL and threonine degradation were also required to carry negative flux in order to minimize deviation from the reduced network 95% confidence flux bounds in the wild-type strain. Because glutamate did not re-enter central carbon metabolism as 2-oxoglutarate (akg) during flux elucidation, glutamate demand across all strains was significantly reduced when compared to k-ecoli74 training data. The behavior of the parameterized model reflected these discrepancies. The most notable underpredictions occurred in *Δpgi*, *Δrpe*, *Δzwf*. In each of these strains, GLUDy flux was underpredicted by more than 50%. The observed discrepancies between the model generated using a projected wild-type flux distribution and the flux distribution used for k-ecoli74 indicates that the information lost due to the absence of atom mappings and stoichiometric information for omitted and simplified reactions is significant. When flux information for these reactions is generated via constraint-based flux projection in a manner consistent with that employed by Khodayari et al. [14, 32] flux predictions in amino acid metabolism are greatly affected. It is, therefore, even for a core metabolism kinetic model, critical that the metabolic network used for flux elucidation include cofactor metabolism and carbon metabolism that are consistent with the kinetic model metabolic network to ensure construction of the most accurate and informative kinetic model possible.

**Table 2:** Flux predictions using kinetic model parameterized with reduced network flux dataset deviating significantly from k-ecoli74 predictions.

Reaction	Strain	Reduced network model prediction (mmol/100 mmol wild-type glucose uptake)	k-ecoli74 prediction (mmol/100 mmol wild-type glucose uptake)
**SERD-L**	*Δpgi*	-2.9	0.3
	*Δrpe*	-7.7	1.9
	*Δedd*	-1.3	0.6
	*Δeda*	-1.3	0.6
	*Δfbp*	-2.4	1.6
	*Δzwf*	-21.4	0.4
	*Δgnd*	-11.4	0.6
**Serine Synthesis**	*Δrpe*	-0.02	6.4
	*Δzwf*	-13.9	8.8
	*Δgnd*	-3.6	7.7
**GLYCL**	*Δpgi*	-0.2	1.5
	*Δrpe*	-0.4	1.2
	*Δedd*	-0.5	0.9
	*Δeda*	-0.5	0.9
	*Δfbp*	-0.4	1.2
	*Δzwf*	-2.0	1.2
	*Δgnd*	-1.1	0.8
**Threonine Degradation**	*Δpgi*	-0.04	0.1
	*Δrpe*	-0.6	0.3
	*Δedd*	-2.1	0.2
	*Δeda*	-2.1	0.2
	*Δfbp*	-2.6	0.2
	*Δzwf*	-0.9	0.4
	*Δgnd*	0.3	0.1
**GLUDy**	*Δpgi*	5.3	16.4
	*Δrpe*	12.7	26.2
	*Δgnd*	19.3	43.9

### Kinetic model predictive evaluation

Target product overproduction was evaluated using k-ecoli74 for seven metabolites using nine engineered strains not used during parameterization. All genetic intervention strategies, target metabolites for overproduction, target reactions used to estimate product yield, predicted product yields, and comparisons with experimental values and k-ecoli457 predictions are reported in Table 3. The reduced model was also used to predict metabolite yields. Reduced model metabolite yields are reported alongside k-ecoli74 metabolite yield predictions in Table 4. Fig D in S3 File depicts the perturbation strategies. Over and under expression were modeled as a fold-change in enzyme level applied to all enzyme complexes of the effected reaction. Because a model for protein synthesis is currently beyond the scope of K-FIT, an x-fold change in gene expression was modeled as an x-fold change in enzyme level. Only enzymes corresponding to perturbed genes had their fold-changes adjusted in the mutant strains. Out of the seven products tested, three were included in k-ecoli74, and four were not. Products present in k-ecoli74 included L-valine, acetate, and malate. L-valine yield was estimated using a lumped L-valine synthesis reaction flux. Acetate exchange flux was used directly to evaluate acetate yield, while MDH flux was used to evaluate malate yield. Products not included in the metabolic network included naringenin, lactic acid, 2,3-butanediol, and glucaric acid. Yields for these metabolites were estimated using flux through reactions producing or consuming precursor metabolites as proxies for heterologous pathways or, in the case of lactic acid, the lactic acid secretion pathway.

**Table 3:** k-ecoli74 metabolite yield predictions under genetic conditions not used for parameterization and comparison with experimental values and values reported for identical strains using the k-ecoli457 model.

			Yield (moles per mole of glucose)/ Percent Overprediction					
Product	Fold Change in Gene Expression	Target Reaction	Experimental/ Reference		k-ecoli74		k-ecoli457	
Malate	0.3x PTAr 10x PPC	MDH	0.15	[69]	0.42	180%	0.84	460%
Acetate	0.1x RPI	Acetate Exchange	0.74	[71]	0.87	18%	0.2	73%
L-Valine	0.1x THRD-L	Valine Synthesis	0.34	[77]	0.03	-91%	0.02	-94%
Naringenin	ΔSUCOAS 0.1x FUM	Tyrosine Synthesis	0.0083 0.0085 0.0055	[76]	0.011	33%	0.015	36%
	ΔMDH 0.1x SUCOAS		0.0033	[76]	0.011	233%	0.0091	176%
Lactic Acid	0.1x ACKr	PGM/ENO	1.07	[78]	1.55	45%	1.2	12%
	ΔACKr		1.13	[78]	1.26	12%	1.11	-2%
2,3-Butanediol	5x PYK	PDH	0.9	[75]	1.12	24%	0.83	-8%
Glucaric Acid	5x NADTRHD	FBA	0.13	[70]	0.76	485%	0.36	177%

Abbreviations: Δ (reaction knockout), PTAr (phosphotransacetylase), PPC (phosphoenolpyruvate carboxylase), RPI (ribose-5-phosphate isomerase), THRD-L (L-threonine deaminase), SUCOAS (succinyl-CoA synthetase), FUM (fumarase), MDH (malate dehydrogenase), ACKr (acetate kinase), PYK (pyruvate kinase), NADTRHD (NAD transhydrogenase), PGM/ENO (phosphoglycerate mutase/enolase, lumped), PDH (pyruvate dehydrogenase), FBA (fructose-bisphosphate aldolase)

**Table 4:** A comparison of k-ecoli74 metabolite yield predictions and reduced model metabolite yield predictions under genetic conditions not used for parameterization.

			Yield (moles per mole of glucose)/ Percent Overprediction					
Product	Fold Change in Gene Expression	Target Reaction	Experimental/ Reference		k-ecoli74		Reduced model	
Malate	0.3x PTAr 10x PPC	MDH	0.15	[69]	0.42	180%	0.16	6%
Acetate	0.1x RPI	Acetate Exchange	0.74	[71]	0.87	18%	-	-
L-Valine	0.1x THRD-L	Valine Synthesis	0.34	[77]	0.03	-91%	0.03	-91%
Naringenin	ΔSUCOAS 0.1x FUM	Tyrosine Synthesis	0.0083 0.0085 0.0055	[76]	0.011	33%	0	-
	ΔMDH 0.1x SUCOAS		0.0033	[76]	0.011	233%	0	-
Lactic Acid	0.1x ACKr	PGM/ENO	1.07	[78]	1.55	45%	1.54	44%
	ΔACKr		1.13	[78]	1.26	12%	1.46	29%
2,3-Butanediol	5x PYK	PDH	0.9	[75]	1.12	24%	1.12	24%
Glucaric Acid	5x NADTRHD	FBA	0.13	[70]	0.76	485%	0	-

Kinetic model predictions for malate and acetate yields were consistent with experimental observations. In both cases, the kinetic model outperformed k-ecoli457 in estimating product yield. In the case of malate, although an approximate three-fold increase in the predicted value was observed compared to the experimental value, TCA cycle flux is required for strain viability, and therefore only a fraction of that flux would be directed towards malate secretion. The reported value is, therefore, a maximum theoretical yield. These results indicate that k-ecoli74 is better suited for predicting central carbon metabolite yields under glucose-rich batch conditions than k-ecoli457.

As expected, L-valine yield was underpredicted by k-ecoli74 by an order of magnitude (similar to k-ecoli457). This underprediction was due to the absence of an excretion pathway known to exist in *E*. *coli* [67], and the absence of flux distributions delivering significant flux towards the L-valine synthesis reaction from the training data. The only reaction consuming L-valine was the L-valine biomass sink reaction. The incomplete pathway coverage offered no drain for flux directed towards L-valine synthesis, and the model was thus incapable of carrying significant flux in that pathway.

k-ecoli74 overpredicted yield for metabolites not included in the network for all six strains tested. This was due to the use of central carbon drains as proxies for pathways not included in the model. This overprediction was expected, since only a fraction of central carbon flux can be directed towards branched pathways if the strain is viable due to the need for carbon flux towards biomass precursors synthesis and energy generation reactions. In three of those six strains, the engineered strain product yield was higher than the wild-type product yield, indicating a favorable re-direction of flux resulting from the genetic perturbations. k-ecoli457, however, outperformed k-ecoli74 when predicting yields in those strains. This was due to the inclusion of pathways peripheral to central carbon metabolism in k-ecoli457, and anaerobic conditions included in the training data which delivered significant flux towards lactic acid secretion, allowing for the generation of a kinetic parameter set better suited to predict lactic acid yield.

The reduced model was only capable of predicting metabolite yields for five of the nine model validation strains tested. In both naringenin overproduction strains and in the glucaric acid overproduction strain, the reduced model predicted no carbon uptake or growth. In the acetate overproducing strain, the model became unstable upon downregulation of RPI expression, and a steady-state flux distribution could not be reached in the perturbed state. The 2,3-butanediol yield prediction was similar to k-ecoli74 prediction, as was L-valine yield, and lactic acid yield under ACKr downregulation. k-ecoli74 outperformed the reduced model significantly, however, when predicting lactic acid yield under the ACKr knockout condition. This indicates that k-ecoli74 is more sensitive to small changes in enzyme level than the model parameterized with the reduced network. The reduced model outperformed k-ecoli74 significantly when predicting malate yield. Reduced model malate yield was within 9% of the experimental value, while k-ecoli74 overpredicted malate yield by 180%. Overall, the reduced model had difficulty predicting metabolite yields when TCA cycle, PP pathway, or oxidative phosphorylation enzyme levels were perturbed. Thus, the feasible prediction space of the core model parameterized by the reduced flux dataset is substantially reduced compared to k-ecoli74, as these pathways represent significant components of core metabolism and potential targets for perturbation strategies that kinetic model of core metabolism are designed to predict.

**Fig 8:**
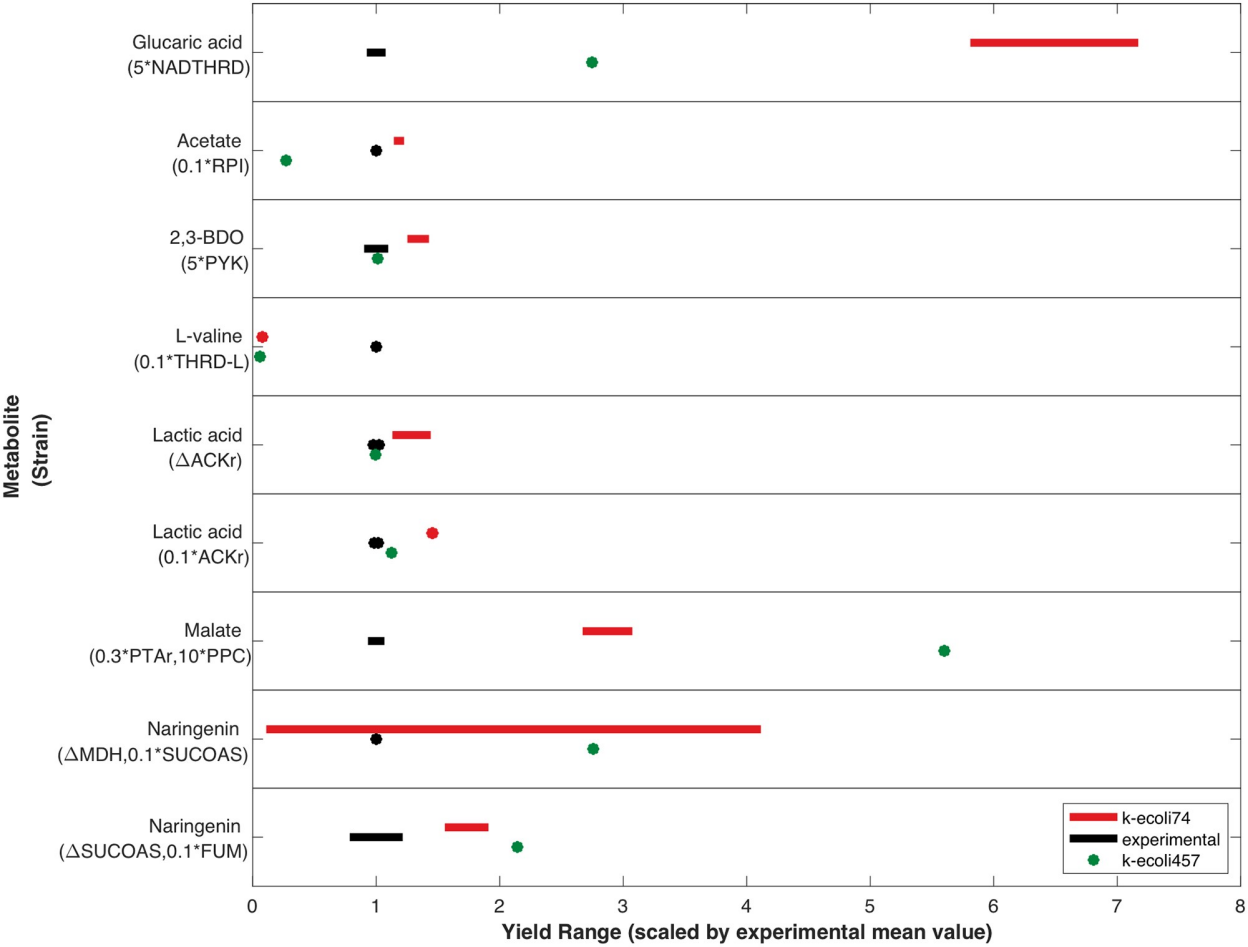
Comparison of overproducing strain metabolite yield ranges when top three models are used to evaluate target metabolite yields with experimental ranges and k-ecoli457 yield values.

Metabolite yield variability was also assessed by comparing the upper and lower bounds of metabolite yields predicted by the three top performing model with experimental ranges. Fig 8 compares the ranges of predicted metabolite yields with experimental ranges. Because the k-ecoli457 model did not provide any information on parameter uncertainty or alternate models with similar fitness, k-ecoli457 yield predictions are represented with a single point. Only naringenin yield produced by the MDH knockout, SUCOAS downregulation strain expanded considerably when the three best models were considered. All other ranges spanned less than 25% of the mean yield value. Overall, narrow ranges of metabolite yields across models confirms the accuracy of k-ecoli74.

### Leave-one-out cross validation

To test k-ecoli74’s ability to predict flux in strains not used for training and determine the impact of parameterization with and without unique flux phenotype in the training data, leave-one-out cross validation was performed. For each cross-validation, a unique kinetic model was parameterized while removing one mutant strain from the training data at a time. Parameterizations were either initialized until a model was generated with SSR less than the best identified model with all data included, or initialized until the recovery rate for a single model fell below the recovery rate obtained during the primary parameterization. In the latter case, the best model identified was used for cross validation analysis. Optimality was tested after each parameterization to ensure a local minimum was reached. Number of parameterizations, recovery rates and minimum SSR for each cross validation are listed in Table 5.

**Table 5:** Leave-one-out cross-validation parameterization results.

Cross-Validation Strain	Number of Parameterizations	Recovery Rate (%)	Minimum SSR
Full Parameterization	500	0.6	338
*Δpgi*	170	0	698
*Δrpe*	24	4.2	299
*Δedd*	94	1.1	257
*Δeda*	36	4.5	214
*Δfbp*	46	2.2	273
*Δzwf*	86	1.2	241
*Δgnd*	14	7.1	323

**Fig 9:**
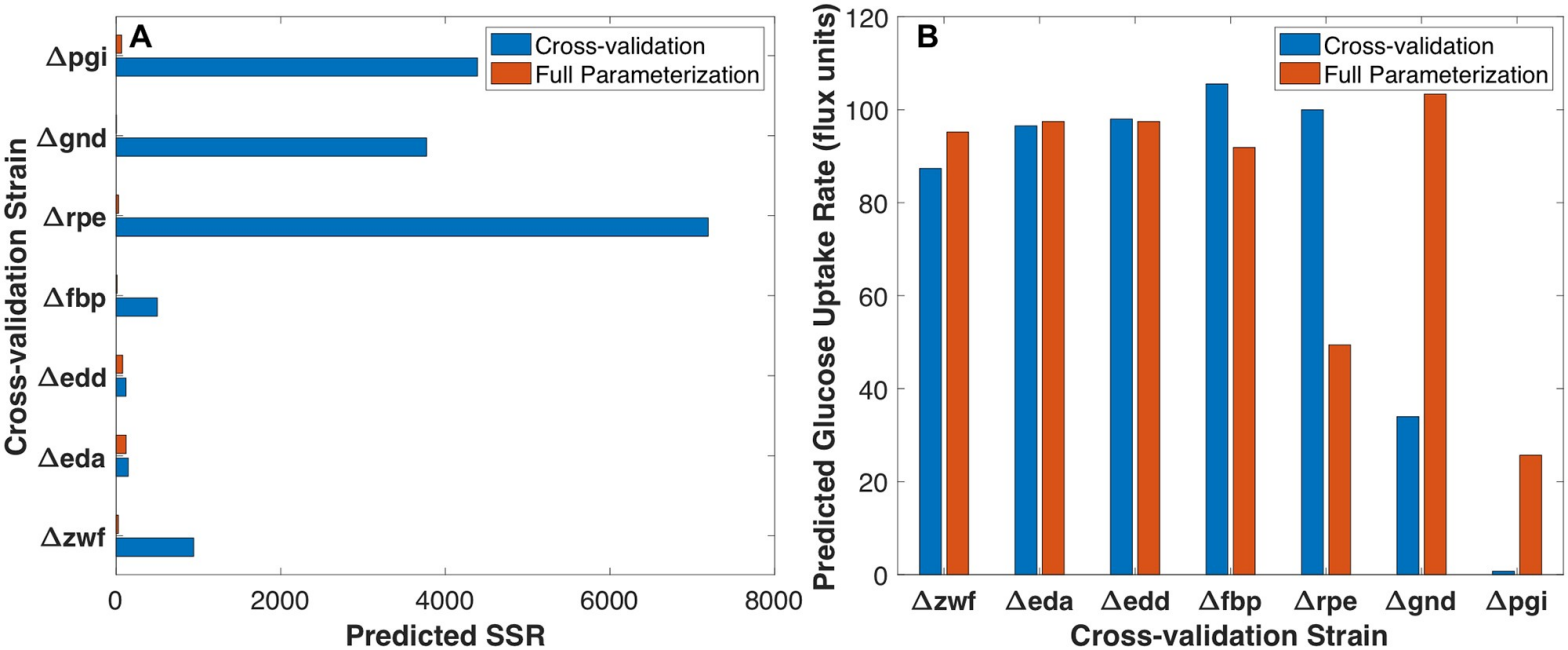
Leave-one-out cross-validation comparison with full parameterization. (A) Predicted cross-validation SSR vs. full parameterization SSR per-strain comparison (B) Predicted cross-validation glucose uptake rate vs. full parameterization glucose uptake rate per-strain comparison.

*Δpgi* was the only strain for which a cross-validation model with SSR less than the best model parameterized with all strains could not be identified. Fig 9A shows the residual error of each strain when the model parameterized without it was used to predict its flux distribution compared with its fitted flux in the full parameterization model. *Δrpe*, *Δpgi*, and *Δgnd* performed the worst during cross-validation, as there were phenotypic attributes of these strains that were unique when compared with the other training datasets. In each of these cases, the cross-validation model was incapable of predicting glucose uptake accurately, and the associated error propagated throughout the entire metabolic network. Fig 9B compares the glucose uptake rate of the cross-validation predictions.

In *Δpgi*, the cross-validation model predicted negligible glucose uptake because the PP pathway kinetic parameters were not well suited for carrying the entirety of flux from g6p. The *Δrpe* cross-validation model wasn’t well-suited for predicting the reduced glucose uptake observed experimentally in the absence of similar training data. Rather than predicting reduced glucose uptake, the *Δrpe* cross-validation predicted increased glycolytic flux compared to experimental fluxes and increased ED pathway flux to compensate for the partial blockage of the nonoxidative PP pathway. Because the training data in the *Δgnd* cross validation lacked a strain delivering significant flux through the ED pathway, the PP pathway and glycolytic kinetic parameters were unable to accommodate the full amount of glucose uptake observed, and the ED pathway was incapable of carrying significant flux. *Δeda* and *Δedd* cross validations models each performed well due to their phenotypic similarities, which ensured that a similar phenotype was included in the training data when one was left out. The resulting models were able to predict the flux distributions of interest well, even though they were the worst fitting strains when all sets were included in the training data. *Δfbp* and *Δzwf* each performed marginally well when compared with the other strains. In *Δfbp*, glycolytic reactions and GLUDy had the highest associated residual errors. The *Δfbp* cross-validation model predicted an increase in glucose uptake flux relative to the wild-type strain, which was propagated through glycolysis. The excess carbon fed into the network was sinked towards glutamate and glutamate-derived amino acid synthesis, rather than the metabolic byproduct, acetate, resulting in the high residual error for GLUDy. In *Δzwf*, a decrease in glucose uptake rate was predicted by the cross-validation model relative to the wild-type strain. This decrease in carbon availability was propagated through glycolysis, where all reactions had high residual error. Similar to *Δfbp*, GLUDy was the central carbon sink reaction with the highest residual error to compensate for the low glucose uptake flux. The results indicate that the model isn’t able to predict metabolic fluxes well when the strains being predicted have phenotypic characteristics that are drastically different from the training datasets.

Overall, the cross-validation results, as well as the full parameterization results, parameterization with reduced flux dataset results, and yield prediction results highlight the large influence that mutant flux dataset selection has on the value of the inferred parameters. Ideally, one would want to assemble a set of mutant flux datasets that uniformly perturb the flux in all major pathways. Unfortunately, this is difficult to *a priori* achieve due to the scarcity of experimental data. Metabolic flux magnitude and directionality dominant in the datasets is often reflected in resultant kinetic parameters, causing inaccurate prediction of unique flux redirections in the context of those used for training data. This implies that parameterization results must be carefully interpreted and flux datasets revealing unique flux redirections may have to be more heavily weighted during the parameterization process.

### Identification of essential regulatory network

**Fig 10:**
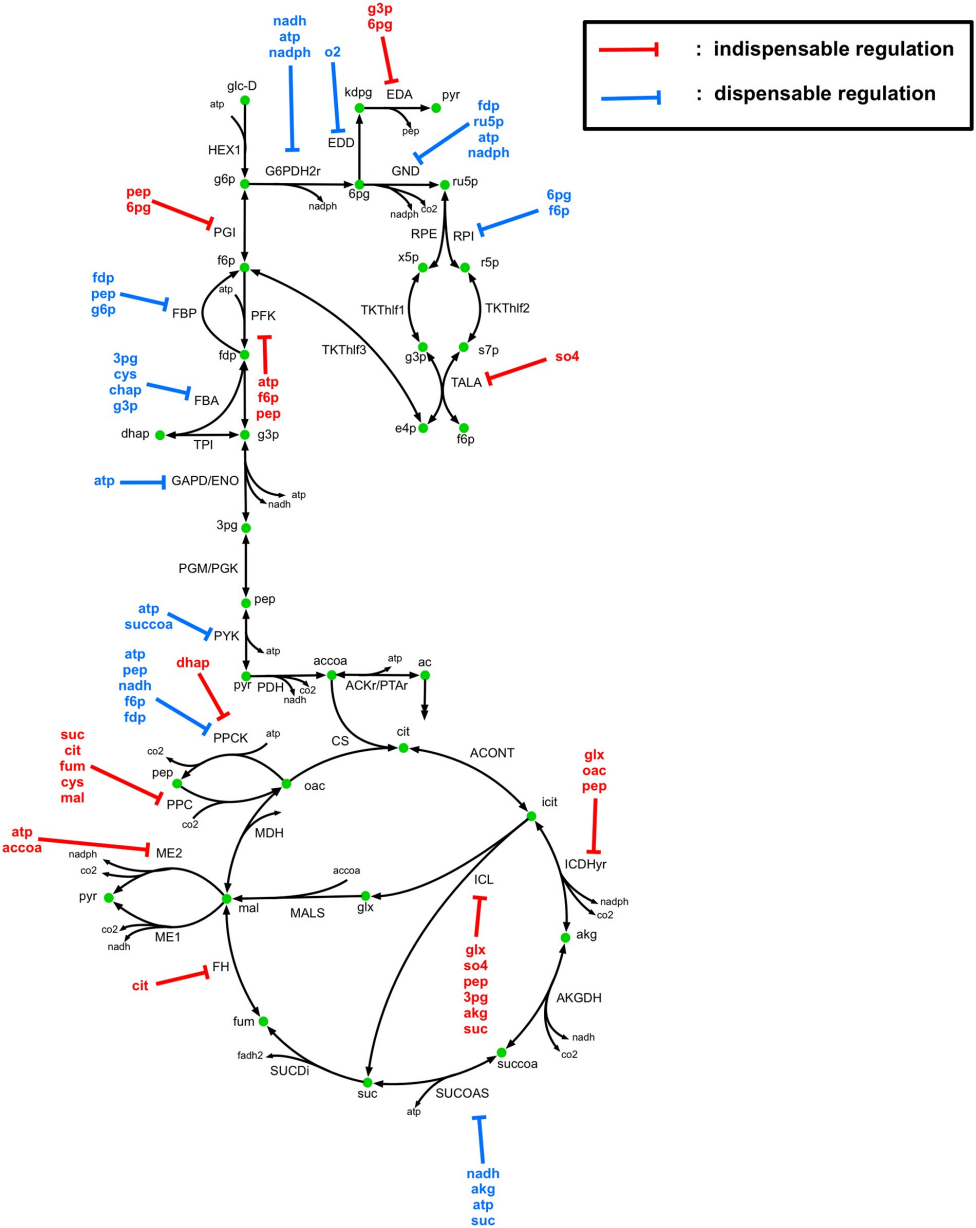
Regulatory mechanisms that are dispensable and indispensable to k-ecoli74 fitness.

To assess the necessity of regulatory mechanisms included in k-ecoli74 and identify nonessential regulations, we compared the locally approximated standard deviation of the inhibition parameters to their parameter values. Regulations were identified as nonessential if the parameter range characterized by the standard deviation had a lower bound of zero. Fig 10 illustrates all regulations determined to be non-essential for model fitness to training data. It was determined that 26 of 55 regulations were essential to model fitness. The results indicate that regulation on one or two key enzymes in a pathway were sufficient to control flux through the entire pathway. The results also indicate that the gradient-based parametrization tended to drive non-essential inhibition constants towards small values (less than one), while essential inhibitions were driven towards large values, as there was a five order of magnitude difference between the average essential inhibition constant value and the average non-essential inhibition constant value. Regulations on the first two glycolytic steps (PGI and PFK) were determined to be essential, while all other glycolytic regulations (on FBP, FBA, GAPD/PGK, and PYK) were dispensable. All regulatory mechanisms in the PP pathway were identified as dispensable except for competitive inhibition of TALA by so4. Regulation of EDA by 6pg and 3pg were sufficient to control ED pathway flux, while EDD regulation by o2 was dispensable. The TCA cycle contained regulations on ICDHyr, SUCCOAS, and FUM. Out of these, SUCOAS regulations were dispensable, with flux controlled by regulation of ICDHyr and FUM. All glyoxylate shunt regulations, and at least one regulation on all anaplerotic reaction with included regulatory mechanisms were identified as essential to model fitness. The results indicate that the inclusion of substrate level regulations in the model is critical for characterizing metabolite pool sizes and enzyme complex fractional abundances, and consequently flux distribution. However, the number of regulatory mechanisms actively controlling flux through the network is limited to a few per pathway controlling key reactions and those peripheral reactions that serve a condition-dependent purpose, such as ME2, PPC, or ICL.

## Discussion

The kinetic parameterization pipeline developed in this study and applied to the development of k-ecoli74 is unique compared to other frameworks used for kinetic model construction in that a single metabolic network was used for flux elucidation and kinetic parameterization. Recent kinetic models of *E*. *coli* metabolism have either relied on experimentally determined kinetic parameters gleaned from a database and a combination of metabolomics and fluxomics data gleaned from a number of sources [87], or relied on fluxomics data generated using a metabolic network inconsistent with that used for kinetic parameterization [14, 32]. A comparison of k-ecoli74 kinetic parameters with those of other models and experimental parameters indicates that the parameterization method can significantly affect the resultant model.

When *K*_*m*_ parameters were derived from the regressed elementary rate constants and compared to those generated using a previously developed core *E*. *coli* kinetic model [14], significant differences were observed (see S2 File). These can be attributed to the differences between growth conditions used in training data, differences in substrate binding and product release orders defined during elementary decomposition between the two models, and the absence of complete cofactor balances in the k-ecoli74 model. Discrepancies were also observed between *K*_*m*_ parameters calculated from both models and experimental ranges, indicating that the optimal set of kinetic parameters for predicting in-vivo flux differs from the optimal kinetic parameters determined from in-vitro experiments. While no definitive reason can be identified based on the results of this study, possible reasons include differences in physiological states in vivo and in vitro (such as macromolecular crowding effects in vivo) [88–93], and differences in physiological state between strains (such as variable enzyme level [36]). The developed workflow, therefore, offers an alternative over kinetic modeling efforts that extract kinetic rate constants from databases because the rate constants are derived from data that is consistent with the conditions for which the model is designed to predict.

The use of the K-FIT algorithm has improved parameterization time by almost an order of magnitude over EM-based parameterization of a core *E*. *coli* model performed by Khodayari et al. [14]. Whereas parameterization of a core kinetic model using the ensemble modeling method generally takes more than a week, kinetic parameterization of k-ecoli74 took less than two days. The parameterization time for k-ecoli74 was greater than that reported by Gopalakrishnan et al. [61] for a similar core model parameterized with a set of toy data. The increase in computational expense was the result of inclusion of experimental training data with competing objectives, as solution reproducibility and parameterization time both improved when leave-one-out cross-validation was performed. This highlights the challenges that can arise when applying modeling frameworks steeped in assumptions to data taken directly from physical systems, and the influence that experimental uncertainty can have on parameterization. Model parameterization time was greater than the reported parameterization time for a core *E*. *coli* model with metabolic network conservation analysis and model stability check performed by Greene et al. [33]. Greene et al. were able to reduce ensemble modeling parameterization time by reducing the solution space based on a pre-evaluation stability analysis for all models in their ensembles, while the K-FIT algorithm reduces parameterization time using a gradient-based search and customized algebraic solvers.

Compared to the previously developed ensemble modeling-parameterized kinetic model of *E*. *coli* core metabolism constructed using an elementary decomposition approach, the model developed here shows improvement in fitness to ^13^C-MFA-derived flux distributions. In this study, 86% of fitted fluxes fell within a single standard deviation of their corresponding ^13^C-MFA value. The ensemble modeling-parameterized core kinetic model of *E*. *coli*, parameterized using the same number of mutant strains, estimated 78% of fitted flux values within a single standard deviation of ^13^C-MFA values [14].

A comparison of the yields for acetate and malate predicted by k-ecoli74 and those predicted by k-ecoli457 highlight the impact that the biological conditions of the cell culture used to generate the labeling data required for for ^13^C-MFA (and consequently, shifts in flux ranges) can have on the resulting kinetic model and prediction accuracy. k-ecoli457 was parameterized using flux distributions for wild-type and mutant strains grown primarily under chemostat conditions with growth rate fixed at a uniform, arbitrarily low value (0.2h^-1^) [36, 94]. The flux distributions exhibited zero acetate excretion across strains, and increased TCA cycle flux (wild-type TCA cycle flux five times higher than in the wild-type flux distribution generated in this study) [36]. Experimental growth conditions under which the acetate [71] and malate [69] yields were measured in the engineered strains were similar to experimental conditions used to generate ^13^C labeling data for this study (*i*.*e*. glucose-rich batch culture, mid-exponential growth phase). As a result, k-ecoli457 overpredicted malate yield by more than 540% and under predicted acetate yield by 75%, while k-ecoli74 overpredicted malate yield by only 180% and predicted acetate yield within 18% of the experimental value. While k-ecoli74 performed better than k-ecoli457 in predicting product yields for metabolites in central carbon metabolism, it was limited in its ability to predict yields for metabolites outside of the k-ecoli74 network. k-ecoli457 outperformed k-ecoli74 when predicting yields for 2,3-butanediol and glucaric acid due to the inclusion of pathways peripheral to central carbon metabolism in the model. k-ecoli457 also outperformed k-ecoli74 when predicting lactic acid yields. This was because the k-ecoli457 training data included datasets generated under anaerobic conditions which exhibited significant flux towards lactic acid secretion. The limited coverage of k-ecoli74 also limited the number of metabolite yields that could be effectively predicted. Whereas k-ecoli457 was able to predict metabolite yield for 24 metabolites across 320 genetic conditions, k-ecoli74 was only able to predict yields for seven metabolites across nine of those conditions. This was due to the non-inclusion of reactions that were perturbed in mutant strains and the non-inclusion of precursor metabolites to the pathway producing the metabolite of interest.

Our results from parameterization with a reduced flux dataset indicate that there is potential for significant discrepancies between kinetics models parameterized using fluxes inferred using the same network compared to fluxes inferred from a simplified model. The differences in the directionality and magnitude of amino acid synthesis and degradation reactions between k-ecoli74 and the kinetic model parameterized with reduced flux dataset demonstrate this. The reduced flux dataset parameterization also show a clear loss in predictive capabilities when the full stoichiometric model is not considered during flux elucidation, as the model parameterized with the reduced flux dataset failed to predict a feasible flux distribution for four of nine validation strains tested. Thus, the inclusion of the full metabolic network in the flux elucidation step of the kinetic parameterization pipeline was essential to successful k-ecoli74 parameterization.

The flux data used for kinetic parameterization also had an impact on the recovery rate of the best solution using the gradient-based methodology. Only 0.6% of solutions recovered agreed with our best model, despite all solutions satisfying local optimality criteria (i.e. zero gradient, non-negative Hessian). Recovery rate increased to 4% and 7%, respectively, when cross-validation was performed for *Δrpe* and *Δgnd*, and in the *Δrpe* cross-validation, 12.5% of solutions yielded SSR values within 10% of the best model when all data was used in fitting. This indicates that the existence of unique phenotypic behavior in mutant strains can lead to the existence of a large number of local minima and decreased recovery rate. It is important to note that our criteria for reproducibility was stringent. The maximum average square residual deviation from the best model was 0.05 per reaction flux. In both cross-validation parameterizations and the primary k-ecoli74 parameterization, many models were generated that produced similar flux distributions to the best model, but did not satisfy the specified reproducibility criteria.

While the developed kinetic parameterization pipeline addresses some issues that have slowed the development and application of kinetic models of metabolism in strain design and lays a framework for kinetic model scale-up in *E*. *coli*, a number of issues still exist. To construct a genome-scale kinetic model using ^13^C-labeling data for parameterization, a comprehensive set of genetic knockout strains across not only upper glycolysis and the PP pathway, but also lower glycolysis and the TCA cycle is required to generate the informative parameter sets for peripheral pathways branching from lower glycolytic and TCA cycle metabolites (such as pyruvate, acetyl-CoA, succinate, oxaloacetate, and 2-oxoglutarate). Another limitation requiring attention is that kinetic models constructed using elementary decomposition methods [30] are able to exclude enzyme concentration from kinetic expressions by assuming that enzyme concentrations do not change from wild type (except for enzymes coded by deleted genes). This assumption simplifies calculations in the absence of comprehensive proteomics data across multiple genetic and/or environmental conditions. Incorporation of kinetic descriptions of transcriptional and translational events into kinetic parameterization procedures would allow for the decoupling of enzyme concentration and elementary kinetic parameters. As an alternative to direct description of protein synthesis events in the cell, protein cost studies have shown that cellular enzyme concentration can be reasonably predicted using kinetic rate expressions for metabolic reactions [95, 96]. It may, therefore, be possible to use kinetic rate expressions for enzyme catalyzed reactions directly to characterize changes in enzyme level across conditions rather than kinetic expressions for transcription and translation. Proteomics data also suggest that changes in enzyme concentration in identical strains across different growth conditions is not simply proportional to the change in growth rate [36, 97], confirming that kinetic parameters regressed using the current framework are growth condition-specific. While the experimental data used in this study was taken from mutant strains with identical growth conditions (*i*.*e*. glucose-rich batch culture, mid-exponential growth phase), a systematic method for updating enzyme level according to growth rate or growth condition would allow for a broader range of applications. This would be a step towards the development of mechanistic kinetic model of metabolism with universal application.

## Supporting information

S1 File Supplementary methods. (DOCX)

S2 File Supplementary results. (DOCX)

S3 File Supplementary figures. (DOCX)

S4 File k-ecoli74 model. (XLSX)

S5 File *K*_*m*_ parameters expressed as a function of elementary kinetic parameters. (DOCX)

S6 File *Vmax* parameters expressed as a function of elementary kinetic parameters and enzyme concentration. (DOCX)

S7 File Michaelis-Menten rate expressions for central carbon reactions in k-ecoli74 metabolic network. (DOCX)

S8 File ^13^C labeling data for wild-type and 7 genetic knockout strains of *E*. *coli*. (XLSX)

S9 File k-ecoli74 results. (XLSX)
