# Supplementary material for: From Escherichia coli mutant 13C labeling data to a core kinetic model: A kinetic model parameterization pipeline
Source: PLoS Comput Biol. 2019 Sep 10;15(9):e1007319. doi: 10.1371/journal.pcbi.1007319 (PMC6759195; doi:10.1371/journal.pcbi.1007319)
Supplement: S1 File — (DOCX) [file pcbi.1007319.s001.docx]

**S1 File: Supplementary Methods**

**^13^C-MFA Least-squares NLP**

We used a procedure consistent with that reported by Gopalakrishnan and Maranas [1] for ^13^C-MFA flux elucidation, summarized below. The purpose of ^13^C-MFA is to identify flux ranges for each reaction in a metabolic network capable of explaining experimentally observed isotopic labeling patterns for a set of measured metabolite fragments. In the ^13^C-MFA framework, isotopic labeling patterns are expressed as mass isotopomer distribution vectors (MDVs). Each MDV is a vector of fractional values corresponding to the percentage of the total pool of that metabolite fragment present in the cell containing any number of ^13^C atoms. Experimental MDVs are generally procured via gas chromatography/mass spectrometry (GC/MS) measurements of amino acid from hydrolyzed proteins [2] or via liquid chromatography/mass spectroscopy (LC/MS) of intermediary metabolites [3] after a cell culture is fed an isotopically labeled substrate and allowed to reach an isotopic steady state. We denote experimentally measured MDVs as $X_{i}^{meas}$, where $i$ represents a single EMU in the set $I$, defined below. In the context of ^13^C-MFA, where only carbon isotopes are considered in MDVs, an EMU is a subset of carbon atoms of any metabolite included in the stoichiometric model [4]. A variance-weighted-least-squares-non-linear-programming problem is used to find the flux distribution that best recapitulates the observed experimental labeling patterns. To help in summarizing ^13^C-MFA procedure and NLP formulation, we define the following sets, parameters, and variables:

**Sets**

Set of EMUs $I=\{i|i=1,\ldots,n_{EMU}\}$

Set of reactions $J=\{j|j=1,\ldots,n_{R}\}$

Set of metabolites $Q=\{q|q=1,\ldots,n_{Q}\}$

$I_{meas}\subseteq I$ is the set of EMUs with corresponding experimental measurements

$J_{meas}\subseteq J$: is the set of all extracellular exchange reactions with corresponding experimental measurements

$J_{ind}\subseteq J$: is the set of linearly independent reactions in the metabolic network corresponding to the rational basis for the right nullspace [5] of the stoichiometric matrix

**Parameters**

$\boldsymbol{S}$: ($n_{Q}x n_{J})$ stoichiometric matrix for the metabolic network

$\boldsymbol{N}$: ($n_{Q}x n_{J_{ind}})$ matrix that is a rational basis for the right nullspace [5] of stoichiometric matrix of the metabolic network

$\boldsymbol{G}$: ($n_{EMU}x n_{EMU} xn_{J}$) matrix that specifies the reactions producing and consuming each EMU in the metabolic network and the source EMUs in reactions producing EMUs

$e_{i}$: measurement variance of MDV $X_{i}^{meas}$, $\forall i \in I_{meas}$

$\sigma_{j}$: standard deviation of reaction flux through reaction $j$, $j\in J_{meas}$

**Variables**

$\phi$ : Variance weighted sum of square residual error

$X_{i}$: MDV of ${EMU}_{i}$ in the EMU network, $, i\in I$

$X_{i}^{meas}$: experimentally measured MDV corresponding to ${EMU}_{i}, i\in I_{meas}$

$v_{j}:$flux through reaction $j$ in the metabolic network, $j \in J$

$\boldsymbol{v}$: ($n_{J} x 1$) vector of fluxes, elements corresponding to the columns of the stoichiometric matrix (***S***)

$\boldsymbol{u}$: ($n_{J_{ind}} x 1$) vector of free fluxes, elements corresponding to the columns of the rational basis for the nullspace (***N***) of the stoichiometric matrix (***S***)

$\boldsymbol{W}$: ($n_{EMU}x n_{EMU}$) matrix that characterizes the rate of EMU production and consumption occurring in the metabolic network and the source EMUs in reactions producing EMUs

$\boldsymbol{A}$: Submatrix of matrix $\boldsymbol{W}$ that characterizes the rate of unknown EMU (defined below) production and consumption occurring in the metabolic network for a given EMU size

$\boldsymbol{B}$: Submatrix of matrix $\boldsymbol{W}$ that characterizes the rate of production and consumption of known EMUs (defined below) occurring in the metabolic network for a given EMU size

***Y***: Set of MDVs of EMUs with known fractional composition for a given EMU size

Using the sets, variables, and parameters defined above, an optimization framework can be established for minimizing the weighted sum of squared residual error (SSR) between a set of experimentally measured MDVs and a set of estimated MDVs that are a function of reaction fluxes in the metabolic network. The ^13^C-MFA optimization formulation is expressed below.

|  | $\min\phi=\sum_{i\in I_{meas}} \left( \frac{X_{i}^{meas}- {X(\boldsymbol{v})}_{i}}{e_{i}} \right)^{2}+\sum_{j\in J_{meas}} \left( \frac{v_{j}^{meas}- v_{j}}{\sigma_{j}} \right)^{2}$ | (6) |
| --- | --- | --- |
|  | Subject to: |  |
|  | $\boldsymbol{S}*\boldsymbol{v}=\boldsymbol{0}, \forall q\in Q$ | (7) |
|  | $v_{j}\geq v_{j}^{LB}, \forall j\in J$ | (8) |
|  | $v_{j}\leq v_{j}^{UB}, \forall j\in J$ | (9) |

Constraint 7 ensures that the system is at metabolic steady state. Constraints 8 and 9 establish upper and lower bounds for estimated flux values based on flux variability analysis (FVA) upper and lower bounds for the system. The optimization formulation above can be simplified by finding a rational basis for the right nullspace of the ***S*** matrix, allowing the vector of fluxes (***v***) to be expressed as a function of a vector of linearly independent fluxes (free fluxes, ***u***) [6, 7] (by first finding the reduced row echelon form of the $\boldsymbol{S}$ matrix). The resulting relationship between the ***v*** and ***u*** is expressed in equation 10:

|  | $\boldsymbol{v} = \boldsymbol{N}*\boldsymbol{u}$ | (10) |
| --- | --- | --- |

Constraints 7, 8, and 9 can then be replaced by a pair of inequality constraints, defined by constrains 12 and 13 in the optimization formulation below, and the objective function can be expressed as a function of ***u***, rather than ***v*** (Equation 11), reducing the dimensionality of the solution space. The resulting simplified optimization formulation is expressed below:

|  | $\min\phi=\sum_{i\in I_{meas}} \left( \frac{X_{i}^{meas}- {X(\boldsymbol{u})}_{i}}{e_{i}} \right)^{2}+\sum_{j\in J_{meas}} \left( \frac{v_{j}^{meas}- v_{j}}{\sigma_{j}} \right)^{2}$ | (11) |
| --- | --- | --- |
|  | Subject to: |  |
|  | $\boldsymbol{N}*\boldsymbol{u}\geq\boldsymbol{v}^{\boldsymbol{LB}}$ | (12) |
|  | $\boldsymbol{N}*\boldsymbol{u}\leq\boldsymbol{v}^{\boldsymbol{UB}}$ | (13) |

The linearly independent flux vector is updated according to the Levenberg-Marquardt algorithm [8] with approximated gradient and Hessian matrices estimated using first-order derivative of residual error between predicted and measured MDV vectors with respect to a reaction network flux vector.

**EMU Decomposition**

We carried out ^13^C-MFA using the EMU framework [4]. In the EMU decomposition approach, sink EMUs are traced back through reactions in the metabolic network to the EMUs corresponding to the carbon source of the system (glucose EMUs in this study). The traceback method used in this study first traced back metabolites to the carbon source, then individual atoms, and finally individual EMUs to preemptively reduce the number of EMUs considered in the final problem. Both the stoichiometric model used for the study and an atom mapping matrix were used to construct the EMU mapping model for each EMU size characterized by the network. An atom mapping matrix characterizes the carbon atom transition from one metabolite to another through a reaction in the network for all atoms and reactions in the network.

**Labeling Distribution Simulation**

The labeling distribution simulation procedure solves for MDV composition in a recursive manner starting with the smallest (size 1) EMU, and then solving for the next largest EMU size iteratively until all unknown MDVs have been estimated [4]. The change in MDV fractional composition over time can be expressed as the sum of all reaction fluxes producing and consuming that particular EMU multiplied by the MDV corresponding to the EMU that is producing it (if it is being produced) or the MDV corresponding to itself (if it is being consumed). At steady state, the conservation of mass principle dictates that the fractional distribution of each MDV must remain constant. Thus, a set of algebraic equations can be constructed to infer a predicted MDV distribution from an estimated flux distribution. We define a matrix form that is readily solvable by first defining a ($n_{EMU}$ x $n_{EMU}$ x $n_{R}$) matrix $\boldsymbol{G}$. Elements in matrix $\boldsymbol{G}$ are determined according to the rules defined in Expression 14 below.

|  | $G_{ii^{'}j}= \left\{ \begin{aligned} 1 if reaction j converts {EMU}_{i^{'}} to {EMU}_{i} \\ -1 if reaction j consumes {EMU}_{i^{'}}={EMU}_{i} \\ 0 otherwise \end{aligned} \right., \forall i\in I, i^{'}\in I, j\in J$ | (14) |
| --- | --- | --- |

When matrix $\boldsymbol{G}$ is multiplied by a vector of fluxes ($\boldsymbol{v}$) across all reactions in the metabolic network, a matrix is produced characterizing the net generation and consumption of each MDV fractional component in in the network, which we refer to as an EMU balance matrix and define as $\boldsymbol{W}$. Equation 15 expresses this operation.

|  | $\boldsymbol{W}= \boldsymbol{G}*\boldsymbol{v}$ | (15) |
| --- | --- | --- |

For a given MDV size, matrix $\boldsymbol{W}$ can be partitioned into submatrices such that columns corresponding to known and unknown MDVs are expressed independently. Equation 16 represents this partition, with submatrix $\boldsymbol{A}$ containing columns corresponding to unknown MDVs and submatrix $\boldsymbol{B}$ containing columns corresponding to known MDVs.

|  | $\boldsymbol{W}=[\boldsymbol{A} \boldsymbol{B}]$ | (16) |
| --- | --- | --- |

The resultant $\boldsymbol{A}$ and $\boldsymbol{B}$ matrices can then be used to characterize the net production and consumption of MDVs of a particular size by multiplying the $\boldsymbol{A}$ and $\boldsymbol{B}$ matrices by all known and unknown MDVs of that size, respectively. In Equation 17, $\boldsymbol{X}$ is the matrix of unknown MDVs of a particular size, and $\boldsymbol{Y}$ is the matrix representing all known MDVs of a particular size. Because the system is at metabolic steady state (i.e. $d\boldsymbol{X}/dt = \boldsymbol{0}$, $d\boldsymbol{Y}/dt = \boldsymbol{0}$), $\boldsymbol{X}$ can be expressed as a function of A, B, and Y (all comprised of known values).

|  | $\boldsymbol{X} = \boldsymbol{A}^{-1}\boldsymbol{BY}$ | (17) |
| --- | --- | --- |

The solution of unknown MDVs is initiated for EMU size one. For single carbon EMUs, the vector of known MDVs corresponds to those MDVs from carbon source metabolites. For EMU sizes greater than one carbon, the vector of known MDVs contains MDVs from carbon source EMUs as well as MDVs for EMUs formed through condensation reactions [4]. Convolution of two MDVs is performed to generate MDVs for EMUs formed through condensation reactions in the metabolic network. After solving for size one EMUs MDVs for size two EMUs are solved, with the size two $\boldsymbol{Y}$ matrix comprised of MDVs from source metabolites and MDVs formed through condensation reactions. This process is repeated recursively for the next largest EMU size until all unknown MDVs in the systems have been calculated.

**Confidence Interval Estimation**

Confidence intervals for reaction fluxes were estimated according to a 95% confidence interval chi-square test (maximum change in SSR=3.84) in order to characterize reaction flux uncertainty [9]. In calculating confidence intervals, we first perform flux coupling analysis to identify the minimum number of reactions for which confidence intervals must be estimated. Then, for a single reaction in the network, we fix reaction flux at a non-optimal value in the increasing direction (finding upper limit) or decreasing direction (finding lower limit) from the optimal value, re-evaluate the remaining unconstrained flux distribution, and evaluate the resulting SSR, continuing to increase (or decrease) the fixed flux value until we hit the threshold for statistical acceptability (defined by the chi-square test statistical significance for one degree of freedom [9]). We define the resulting range of values for that reaction as the confidence interval and repeat the process for each uncoupled reaction in the network. Coupled reaction flux ranges that were not evaluated via the method described are determined using flux variability analysis with the identified confidence interval for uncoupled reactions as bounds.

**K-FIT Kinetic Parameterization Least-squares NLP**

The gradient-based K-FIT kinetic parameterization algorithm was used to parameterize k-ecoli74 and is summarized below. To help in summarizing K-FIT parameterization procedures, we define the following sets and variables:

**Sets**

Set of metabolites $I=\{i|i=1,\ldots,n_{N}\}$

Set of reactions $J=\{j|j=1,\ldots,n_{R}\}$

Set of elementary steps $L=\{l|l=1,\ldots,n_{L}\}$

Set of enzyme complexes $P=\{p|p=1,\ldots,n_{P}\}$

Set of genetic perturbation conditions $C=\{c|c=1,\ldots,n_{C}\}$

$WT \subseteq C$: wild-type genetic condition

$L_{jb} \subseteq L$: is the set of backward elementary reaction fluxes in reaction $j\in J$ in the metabolic network, $\forall j\in J$

$L_{jf} \subseteq L$: is the set of forward elementary reaction fluxes in reaction $j\in J$ in the metabolic network, $\forall j\in J$

**Parameters**

$R_{jp}= \left\{ \begin{aligned} 1 if enzyme complex p\in P is generated in reaction j\in J \\ 0 otherwise \end{aligned} \right.$ $\forall j\in J, p\in P$

$Q_{pl}= \left\{ \begin{aligned} 1 if enzyme complex p\in P is consumed in elementary step l\in L \\ 0 otherwise \end{aligned} \right.$ $\forall j\in J, l\in L$

$V_{jc}^{meas}$: ^13^C-MFA-determined net flux through reaction $j\in J$ under condition $c\in C$

$V_{j}^{WT}$: ^13^C-MFA-determined net flux through reaction $j\in J$ under wild-type condition

$\sigma_{jc}$: standard deviation of reaction flux through reaction $j$, $j\in J$ under condition $c\in C$

**Variables**

$\phi$ : Variance weighted sum of square residual error

$e_{p}^{c}$: fractional abundance of enzyme complex $p\in P$ under condition $c\in C$

$v_{l}^{c}$: flux through elementary step $l\in L$ under condition $c\in C$

$k_{l}$: elementary kinetic parameter for elementary step $l\in L$

$s_{i}^{c}$: fold change in concentration from wild-type strain concentration of metabolite $i\in I$ under condition $c\in C$

$V_{j}^{c}$: net flux through reaction $j\in J$ under condition $c\in C$

K-FIT defines the problem of kinetic parameterization in the form of a weighted least-squares optimization, whereby the SSR of predicted flux distributions across a set of genetic knockout conditions and ^13^C-MFA-generated flux distribution for the same mutant strains is minimized. Predicted mutant flux distributions are obtained using a set of kinetic parameters estimated using the wild-type ^13^C-MFA generated flux distribution. A set of key assumptions allows for computation of key variables ($e_{p}^{c}$, enzyme complex fractional abundance and $s_{i}^{c}$, metabolite concentration fold-change from wild-type concentration) required for estimating a flux distribution across conditions not used to generate kinetic parameters and the formulation of the optimization problem that can be used to find an optimal set of kinetic parameters.

**Kinetic Parameterization Assumptions**

1. In accordance with the Briggs and Haldane approach [10], fractional abundance of each enzyme complex in any reaction in the network is at steady state ($\frac{de_{p}^{c}}{dt}=0$, $\forall p\in P$, $c\in C$).
2. Total enzyme concentration for any enzyme in the reaction is assumed to be constant across conditions.
3. Cells are at metabolic steady state across all conditions ($\frac{ds_{i}^{c}}{dt}=0$, $\forall i\in I$, $c\in C$).

Assumptions 1 and 2 allows for a set of algebraic equations to be established expressing enzyme complex fractional abundance as a function of metabolite concentration fold change from wild type concentration and elementary kinetic parameters. Assumption 3 allows for metabolite concentration to be expressed as a function of elementary kinetic parameters and enzyme complex fractional abundance. Assumption 2 also allows for enzyme complex concentration under any condition to be normalized by total enzyme concentration and expressed as fractional abundance with the property that the summation of all normalized enzyme complex concentration for a given enzyme under a given condition must equal one, expressed in Equation 18.

|  | $\sum_{p=1}^{n_{P}} R_{jp}*e_{pc}=1, \forall j\in J, c \in C$ | (18) |
| --- | --- | --- |

Assumptions 1, 2 and 3 also allow for the integration of metabolite concentration with respect to time, a key step in kinetic parameterization methods (*e.g.* EM method [11]) that is also a computational bottleneck. To minimize computational expense, a combination of a fixed-point iterative method, Newton’s method, and a semi-implicit integration method are used to estimate mutant strain metabolite pool size and enzyme complex fractional abundance. For a complete description of the integration methods used for determining metabolite concentration fold change from wild-type concentration and enzyme complex fractional abundance under genetically perturbed conditions, refer to Gopalakrishnan et al. [12].

**Kinetic Parameterization Optimization Formulation**

The least-square fitting problem used in K-FIT made possible by the previously defined assumptions is summarized below.

|  | $\min_{k} \phi= \sum_{c=1}^{C} \sum_{j\in J_{C}^{meas}} \left( \frac{{V(\boldsymbol{k})}_{j}^{c}-V_{jc}^{meas}}{\sigma_{jc}} \right)^{2}$ | (19) |
| --- | --- | --- |
|  | Subject to: |  |
|  | $v_{b}^{WT}>0, \forall b\in L_{jb},\forall j\in J$ | (20) |
|  | $-v_{b}^{WT}< V_{j}^{WT}, \forall b \in L_{jb},\forall j\in J$ | (21) |
|  | $v_{f}^{c}= V_{j}^{c}+v_{b}^{c}, \forall f \in L_{jf}, b\in L_{jb},j\in J, c\in C$ | (22) |
|  | $0 < e_{p}^{c} < 1, \forall p \in P, c \in C$ | (23) |
|  | $\sum_{p=1}^{n_{P}} R_{jp}*e_{p}^{c}=1, \forall j\in J, c \in C$ | (24) |
|  | $k_{l} = \frac{v_{l}^{WT}}{\sum_{p=1}^{n_{p}} Q_{pl}*e_{p}^{WT}}, \forall l \in L$ | (25) |

The objective function (Equation 19) minimizes the difference between net reaction flux under genetically perturbed conditions and ^13^C-MFA flux values. Constraints 20, 21, and 22 ensure that the elementary step flux is always positive. Constraints 23 and 24 ensure that the enzyme complex fractional abundance is always positive, and also that enzyme complex fractional abundances for any reaction in the network always sum to unity. Combined, constraints 20, 21, 22, and 23 also ensure elementary kinetic parameters always assume positive values. Constraint 25 is used to defines elementary kinetic parameters as a function wild-type elementary reaction fluxes and wild-type enzyme complex fractional abundances. Predicted mutant strain net flux is a function of those elementary kinetic parameters. The method for deriving net reaction flux from a set of elementary kinetic parameters is described in Gopalakrishnan et al. [12]. The decision variables in the formulation are wild-type backward elementary reaction flux ($v_{b}^{WT}$) and wild-type enzyme complex fractional abundance ($e_{p}^{WT}$), from which all other variables are calculated. Wild-type enzyme complex fractional contribution and backward elementary reaction flux vectors are updated at each iteration of K-FIT according to approximated gradient and Hessian matrices using the Levenberg-Marquardt algorithm [8].

**Gradient-based Kinetic Parameterization Algorithm**

The mathematical properties of the kinetic rate expressions for the production and consumption of all metabolites and enzyme complexes in the metabolic network resulting from Assumptions 1, 2, and 3 stated above allow for the construction of a nested algorithm within K-FIT to solve the defined optimization problem. The nested K-FIT algorithm is as follows:

**Step 0**: Randomly initialize feasible set of enzyme fractions and backwards elementary fluxes satisfying wild- type flux distribution.

**Step 1**: Evaluate $\boldsymbol{k}$ based on $\boldsymbol{e}^{\boldsymbol{WT}}$ and $\boldsymbol{v}_{\boldsymbol{b}}^{\boldsymbol{WT}}$.

**Step 2**: Set $s_{ic}^{(0)}=1 \forall i \in I, c \in C$

**Step 3**: Compute $\boldsymbol{e}$ given $\boldsymbol{s}^{\boldsymbol{(0)}}$ and $\boldsymbol{k}$ ($\forall c \in C$).

**Step 4**: Compute $\boldsymbol{s}$ given $\boldsymbol{e}$ computed in Step 3 and $\boldsymbol{k}$ ($\forall c \in C$)

**Step 5**: If $\left\| \boldsymbol{s}-\boldsymbol{s}^{\boldsymbol{(0)}} \right\|>tol$, set $\boldsymbol{s}^{\boldsymbol{(0)}}=\boldsymbol{s}$ and go to Step 3. Else, go to Step 6 ($\forall c \in C$).

**Step 6**: Report $\boldsymbol{e}$ and $\boldsymbol{s}$ as steady state enzyme complex fractional contribution and substrate concentration fold-change relative to wild-type, respectively ($\forall c \in C$).

**Step 7**: Infer flux distribution from $\boldsymbol{e}$, $\boldsymbol{s}$, and $\boldsymbol{k}$ ($\forall c \in C$).

**Step 8**: Evaluate residual error across metabolic network, calculate SSR and approximate gradient, Hessian matrices.

**Step 9**: If $max(gradient)>tol$ set $\boldsymbol{x}^{\boldsymbol{(0)}}=\boldsymbol{x}$ and go to Step 1. Else, optimal solution has been found, terminate.

1. Gopalakrishnan S, Maranas CD. 13C metabolic flux analysis at a genome-scale. Metab Eng. 2015;32:12-22.

2. Leighty RW, Antoniewicz MR. Parallel labeling experiments with [U-13C]glucose validate E. coli metabolic network model for 13C metabolic flux analysis. Metab Eng. 2012;14(5):533-41.

3. Schaub J, Mauch K, Reuss M. Metabolic flux analysis in Escherichia coli by integrating isotopic dynamic and isotopic stationary 13C labeling data. Biotechnol Bioeng. 2008;99(5):1170-85.

4. Antoniewicz MR, Kelleher JK, Stephanopoulos G. Elementary metabolite units (EMU): a novel framework for modeling isotopic distributions. Metab Eng. 2007;9(1):68-86.

5. Friedberg S, Insel A, Spence L. Linear Algebra. 4 ed: Pearson Education Limited; 2014.

6. Wiechert WS, C., de Graaf AA, Marx A. Bidirectional Reaction Steps in Metabolic Networks: II. Flux Estimation and Statistical Analysis. Biotechnology and Bioengineering. 1997;55(1):118-35.

7. Gopalakrishnan S, Pakrasi HB, Maranas CD. Elucidation of photoautotrophic carbon flux topology in Synechocystis PCC 6803 using genome-scale carbon mapping models. Metab Eng. 2018;47:190-9.

8. More JJ. The Levenberg-Marquardt Algorithm: Implementation and Theory. Numerical Analysis. 1978;630.

9. Antoniewicz MR, Kelleher JK, Stephanopoulos G. Determination of confidence intervals of metabolic fluxes estimated from stable isotope measurements. Metab Eng. 2006;8(4):324-37.

10. Briggs GE, Haldane JBS. A note on the kinetics of enzyme action. Biochem J. 1925;19(2):338-9.

11. Tran LM, Rizk ML, Liao JC. Ensemble modeling of metabolic networks. Biophys J. 2008;95(12):5606-17.

12. Gopalakrishnan S, Dash S, Maranas CD. K-FIT: An accelerated kinetic parameterization algorithm using stead-state fluxomic data. bioRxiv. 2019.
