# Supplementary material for: From Escherichia coli mutant 13C labeling data to a core kinetic model: A kinetic model parameterization pipeline
Source: PLoS Comput Biol. 2019 Sep 10;15(9):e1007319. doi: 10.1371/journal.pcbi.1007319 (PMC6759195; doi:10.1371/journal.pcbi.1007319)
Supplement: S2 File — (DOCX) [file pcbi.1007319.s002.docx]

**S2 File: Supplementary Results, ^13^C-MFA, sensitivity analysis and range comparison of elementary kinetic parameters and Michaelis-Menten kinetic parameters**

All reaction and metabolite abbreviations are defined in S4 File

**^13^C-MFA**

Fluxes were elucidated for wild-type and seven single gene deletion mutant strains of *E. coli*. The weighted sum of squared residuals (SSR) values and degrees of freedom (DOF) for each parameterization are reported in Table S1. DOF were determiend according to the statistical definition as the difference between the number of datapoints and the number of parameters fitted [1]. Variation in DOF was due to differences in data availability between strains [2]. Each reported SSR fell within the statistical acceptable threshold for the specified DOF according to a chi-squared distribution test. Experimental metabolite fragment mass isotopomer distributions and uptake/excretion fluxes used for each flux elucidation are listed in the S8 File.

**Table S1. SSR value and degrees of freedom from ^13^C-MFA flux elucidation for wild-type and 7 single gene deletion mutant strains.**

| **Strain** | **DOF** | **SSR** |
| --- | --- | --- |
| Wild-type | 34 | 13.4 |
| *Δpgi* | 36 | 12.5 |
| *Δrpe* | 21 | 20.7 |
| *Δeda* | 22 | 28.8 |
| *Δedd* | 35 | 17.1 |
| *Δzwf* | 23 | 23.9 |
| *Δfbp* | 35 | 20.7 |
| *Δgnd* | 35 | 27.5 |

Due to the dispensability of the FBP, EDA, and EDD reactions in the wild-type strain, flux ranges relative to glucose uptake were similar to the wild-type strain in *Δfbp*, *Δeda*, and *Δedd*. Confidence ranges (95% intervals) for these strains were either completely subsumed within or showed significant overlap with the wild-type flux distribution. *Δeda* glucose uptake rate was similar to wild-type glucose uptake rate, with its mean value increased by only 2% relative to the mean wild-type glucose uptake rate. A 9% decrease in *Δedd* mean glucose uptake rate and a 10% decrease in *Δfbp* mean glucose uptake rate relative to the mean wild-type glucose uptake rate were deduced. Flux ranges across central carbon metabolism decreased or increased in proportion to glucose uptake rate when compared to the wild-type flux distribution. Experimental glucose uptake rates, standard deviations, and the statistical significance of differences between mutant glucose uptake rate and wild-type glucose uptake rate are provided in S8 File. *Δgnd*, *Δzwf*, *Δrpe*, and *Δpgi* strains each underwent carbon flux redirection from the wild-type strain. Fig A in S3 File compares the central carbon metabolism flux distributions for *Δgnd*, *Δzwf*, *Δrpe*, *Δpgi*, *Δeda*, *Δedd*, and *Δfbp* with the wild-type strain.

The *Δpgi* strain flux distribution (Fig A (A) in S3 File) deviated significantly from wild-type flux across all major pathways except the pentose phosphate (PP) and Entner-Doudoroff (ED) pathways. Because the PGI enzyme was knocked out in *Δpgi*, all carbon entering the cell is redirected through the oxidative pentose phosphate (OPP) pathway. The HEX1 flux was reduced to 25% of the wild-type value, and as a result OPP pathway flux decreased to 92% of the wild-type. Lower glycolytic flux was reduced to 15% of the wild-type strain flux. Acetate production was the lowest out of all mutant strains considered in this study, with a 95% confidence interval upper bound of 0.75 mmol/100 mmol wild-type glucose uptake. The decrease in acetate excretion was accompanied by an increase in carbon entering the tricarboxylic acid (TCA) cycle relative to glucose uptake. Compared to the wild-type strain, *Δpgi* exhibited 37 mmol/100 mmol glucose uptake of CS flux, while wild-type was characterized by only 17 mmol/100 mmol glucose uptake of CS flux. Relative to glucose uptake, increased flux was also directed through GLUDy and ASPTA reactions. GLUDy and ASPTA fluxes were 68 and 19 mmol/100 mmol glucose uptake, respectively, compared to 50 and 14 mmol/100 mmol glucose uptake in the wild-type strain. This indicates the re-direction of flux from acetate excretion to the TCA cycle caused increased biomass synthesis efficiency in *Δpgi*. These flux results are consistent with the results reported recently by Long et al. [3].

Glucose uptake in the *Δrpe* strain was approximately half that of the wild-type strain. Upper glycolysis carried the majority of carbon flux entering the central carbon metabolism, and OPP pathway activity made up less than 10% of carbon flux consuming g6p, compared to 26% in the wild-type strain. ED pathway flux was also reduced by 69% compared to the wild-type strain. OPP pathway flux was directed through RPI to generate r5p for nucleotide biosyntheisis, while nonoxidative PP pathway flux was reversed compared to the wild-type strain to generate e4p for amino acid biosynthesis. While glycolytic and PP pathway reactions carried less flux than the wild-type strain, flux directed into the TCA cycle via CS was similar to the wild-type flux distribution. Despite similar carbon flow into the TCA cycle, flux towards amino acids produced from cataplerotic pathways was decreased in *Δrpe* compared to the wild type strain. This combined with increased ATP sink flux points to increased demand for energy and cofactors to compensate for the reduction in NADPH production resulting from decreased OPP pathway flux as a reason for increased TCA cycle flux relative to glucose uptake.

*Δzwf* glucose uptake flux was increased by 4% compared to the wild-type strain, while OPP and ED pathways carried no carbon flux. As a result of the increased glucose uptake, glycolytic and TCA cycle reaction fluxes were also increased compared to the wild-type strain. To compensate for the elimination of the OPP pathway, nonoxidative PP pathway directionality was reversed to generate e4p and r5p for amino acid and nucleotide biosynthesis, respectively. The magnitude of increase in reaction flux was consistent across the metabolic network, fluxes were increased between 5% and 16% across central carbon metabolism compared to the wild-type strain.

*Δgnd* strain glucose uptake rate was 5% lower than in the wild-type strain. Glycolytic flux was also reduced. Flux through G6PDH2r was increased by 9% compared to the wild-type strain. However, as GND was removed from metabolism, all flux directed through G6PDH2r was directed through the ED pathway, and a 22-fold increase in ED pathway flux was observed compared to the wild-type strain. Acetate excretion was increased by 14% compared to the wild-type strain. GLUDy flux was decreased by 18% compared to wild-type, and the increased carbon flux through TCA cycle reactions downstream of akg was alleviated by PPCK, which was active in *Δgnd* but not in the wild-type strain.

**Kinetic Parameter Sensitivity Analysis**

**Local Sensitivity of Elementary Kinetic Parameters**

Local sensitivity of elementary kinetic parameters was determined by approximating the standard deviation of each elementary kinetic parameter. A coefficient of variation (CV) value of p% indicates that a p% change in the parameter is associated with one standard deviation. Therefore, small values of CV indicate very low uncertainty in the corresponding inferred parameter. The non-biomass reactions with elementary parameter CVs greater than 1% and their corresponding maximum CVs are presented in Table S2. The CV was less than 1% for 742 out of 974 elementary kinetic parameters, indicating that even a slight perturbation in the value of the majority of elementary kinetic parameters relative to their mean values would significantly affect the SSR. Elementary parameters with CVs greater than 100% belonged primarily to reactions in central carbon metabolism due to the inclusion of substrate-level inhibitions on reactions from those pathways (the majority of elementary kinetic parameters with coefficients of variation greater than 100% were inhibition constants). SSR was the least sensitive to elementary parameters from glycolysis, as 20 of the 58 elementary parameters with CVs greater than 100% belonged to glycolytic reactions (PGI, PFK, FBP, FBA, GAPD/PGK, PYK). The remaining reactions with large CVs were primarily PP pathway (G6PDH2r, GND, RPI, TKT), TCA cycle (ACONT, ICDHyr, SUCOAS, SUCDi, FUM) and ED (EDD) pathway inhibition constants. The majority of inhibition constants belonging to ME1, PPC, and PPCK also had CVs greater than 100%. All glycolytic reactions with large CV values, G6PDH2r, ACONT, SUCOAS, ME1, PPC, and PPCK had multiple inhibitors, indicating that the function of multiple inhibitors on those reactions was redundant. The results indicate that for those reactions, a single inhibition mechanism regulated carbon flow, the other inhibition mechanisms had little effect on carbon flow, and many inhibition mechanisms within the same pathway were redundant due to similarities in inhibitor concentrations.

**Table S2. Non-uptake, non-lumped, non-biomass reactions with elementary parameter CVs greater than 1%**

| **Reaction** | **Maximum CV (%)** | **Parameter Type with CV>1% (i = inhibitory, e = elementary step)** |
| --- | --- | --- |
| PGI | 44.2 | i,e |
| PFK | 283 | i |
| FBP | 5.07x10^7^ | i,e |
| FBA | 1.73x10^6^ | i,e |
| TPI | 1.0 | e |
| PYK | 1.95x10^5^ | i |
| G6PDH2r | 9.43x10^4^ | i,e |
| GND | 253 | i |
| RPE | 10.3 | e |
| RPI | 1.27x10^7^ | i,e |
| TKT | 158 | e |
| TALA | 9.35 | i |
| EDD | 1.87x10^5^ | i, e |
| EDA | 2.7 | i |
| PDH | 4.14 | e |
| CS | 5.25 | e |
| ACONT | 474 | e |
| ICDHyr | 3250 | i |
| AKGDH | 61.8 | e |
| SUCOAS | 7.9x10^4^ | i,e |
| SUCDi | 715 | e |
| FUM | 237 | e |
| MDH | 7.21 | e |
| ICL | 81.5 | i,e |
| MALS | 1.27 | e |
| ME1 | 348 | i,e |
| ME2 | 17.6 | e |
| PPC | 5.28x10^4^ | i,e |
| PPCK | 1220 | i |
| GLUDy | 88.8 | e |
| ASPTA | 702 | e |
| GLYCL | 3370 | e |
| SERD-L | 3.79 | e |

$\boldsymbol{K}_{\boldsymbol{m}}$ **and** $\boldsymbol{Vmax}$ **sensitivity**

The sensitivity of the quality of fit with respect to $K_{m}$ and $Vmax$ parameters was assessed by finding their spanned values in all models whose optimal SSR value was within 10% of the best model. Expressions for $K_{m}$ and $Vmax$ parameters in terms of elementary kinetic parameters for central carbon metabolism reactions are provided in S5 File and S6 File, respectively. Michaelis-Menten kinetic rate expressions for central carbon metabolism reactions derived using the King-Altmann method [4] are provided in S7 File. Two additional models out of 500 yielded SSR values within 10% of the best model. Despite having significantly different elementary kinetic parameter values, predicted flux ranges of fitted reaction fluxes were similar between the three models. A total of 74% of predicted central carbon reaction fluxes from the two additional models varied by less than 10% from the corresponding best model predictions. Table S3 summarizes the results, indicating the number of $K_{m}$ and $Vmax$ parameters per pathway, the number of unresolved parameters per pathway, and the percent of unresolved parameters belonging to that pathway. Overall, 29% of $K_{m}$ and 15% of $Vmax$ ranges assessed were expanded by more than 10 times the mean value of the best model (i.e. were unresolved).

**Table S3. Summary of** $\boldsymbol{K}_{\boldsymbol{m}}$ **and** $\boldsymbol{Vmax}$ **parameter range expansion results**

| **Pathway** | **Number of Parameters** | | **Number of Unresolved Parameters** | | **Percentage of Unresolved Parameters Belonging to Pathway** | |
| --- | --- | --- | --- | --- | --- | --- |
|  | $\boldsymbol{Km}$ | $\boldsymbol{Vmax}$ | $\boldsymbol{Km}$ | $\boldsymbol{Vmax}$ | $\boldsymbol{Km}$ | $\boldsymbol{Vmax}$ |
| Glycolysis | 83 | 16 | 6 | 2 | 1.6 | 13 |
| PP Pathway | 69 | 13 | 32 | 0 | 8.9 | 0 |
| TCA Cycle | 73 | 18 | 31 | 2 | 8.1 | 13 |
| ED Pathway | 9 | 2 | 4 | 0 | 1.1 | 0 |
| Glyoxylate Shunt | 18 | 2 | 7 | 0 | 1.9 | 0 |
| Anaplerotic/Cataplerotic | 83 | 8 | 5 | 2 | 1.4 | 13 |
| Amino Acid Reactions | 857 | 31 | 255 | 7 | 71 | 47 |
| Energy Metabolism | 49 | 4 | 11 | 2 | 3.1 | 13 |
| Uptake/Export Reactions | 5 | 3 | 2 | 0 | 0.6 | 0 |

Fig B (A) in S3 File shows the average $Km$ range expansion per reaction across carbon pathways in the model. The majority of unresolvable $K_{m}$ parameters belonged to the PP pathway (GND, RPE, TKT, TALA), TCA cycle reactions (CS, ACONT, ICDHyr, AKGDH, SUCOAS, FUM), and reactions peripheral to central carbon metabolism (i.e. lumped amino acid synthesis reactions). The poor resolvability of $K_{m}$ ranges for those reactions is reflected in the average $K_{m}$ range expansion per reaction. Large $K_{m}$ ranges were associated with small $K_{m}$ lower values (i.e. <10^-7^ M) for 163 of 1246 $K_{m}$ parameters compared (60% of unresolved $K_{m}$ parameters). A total of 258 of 1246 parameters whose range was not significantly expanded had negligible lower bounds (i.e. < 10^-7^ M). Thus $K_{m}$ parameters with significant range expansion were likely to have small lower bounds, but many parameters had ranges spanning several orders of magnitude in the best model, indicating a general unresolvability. PP pathway, TCA cycle, glyoxylate shunt, and ED pathway reactions were most likely to have unresolved $K_{m}$ ranges. A total of 32 of 69 PP pathway $K_{m}$ parameters, 31 of 73 TCA cycle $K_{m}$ parameters, four of nine ED pathway $K_{m}$ parameters, and seven of 18 glyoxylate shunt pathway $K_{m}$ parameters were unresolved. These results indicate that several reactions from each pathway in central carbon metabolism may be operating at $Vmax$.

Expansion of $Vmax$ parameters was less frequent than expansion of $K_{m}$ parameters, and the average $Vmax$ range expansion per reaction is shown in Fig B (B) in S3 File. Unresolvable $Vmax$ parameters belonged primarily to the reactions peripheral to central carbon metabolism. Glycolytic reactions PYK and GAPD/ENO each had a single unresolved $Vmax$ parameter, while FUM and MDH each had a single unresolved $Vmax$ parameter. No PP pathway, ED pathway, or glyoxylate shunt $Vmax$ parameters were unresolved. Range expansions were most prevalent in reactions carrying little flux across strains, as nine of 40 anaplerotic/cataplerotic or amino acid synthesis $Vmax$ ranges were unresolved. $Vmax$ parameters with unresolvable ranges were correlated to the reactions with significant $K_{m}$ range expansions for only three of 15 (20%) reactions with unresolvable $Vmax$ parameters. A total of 32 of 36 (89%) reactions with unresolved $K_{m}$ ranges had well-resolved $Vmax$ parameters. Therefore, a negative correlation between the resolvability of $K_{m}$ and $Vmax$ parameters for any given reaction exists in k-ecoli74. The existence of a significant number of $K_{m}$ parameters with negligible lower bounds and well resolved $Vmax$ parameters also indicates that enzyme saturation kinetics may play a role in controlling flux through the metabolic network (i.e. $Vmax$ controls reaction flux) for many reactions.

$\boldsymbol{K}_{\boldsymbol{m}}$ **Range Comparisons**

$K_{m}$ ranges were compared to both experimental data and to parameters estimated from a previously constructed core kinetic model (EM core, constructed using the EM method and parameterized using fluxomics data generated under chemostat conditions with a growth rate fixed at 0.2h^-1^ across all conditions) [5]. In the case of the EM core model [5], elementary kinetic parameter scaling was performed using metabolomics data that was generated under identical conditions to the ^13^C-labeling data [6]. For both models, only $K_{m}$ parameters with all metabolomics data required for elementary parameter scaling were considered for comparison. A total of 53 $K_{m}$ ranges from k-ecoli74 were compared to experimental ranges. Overlapping ranges were identified for 17% of $K_{m}$ parameters. A total of 45 $K_{m}$ ranges were compared with those calculated using the EM core model [5]. Overlapping ranges between the two models were identified for only 7% of $K_{m}$ parameters compared. $K_{m}$ ranges determined using the elementary parameters from the EM-parameterized model were also compared to experimental ranges. A total of 26 EM core $K_{m}$ parameters had sufficient metabolomics data for comparison with experimental ranges. Out of those, only 19% of $K_{m}$ ranges showed overlap with the experimental ranges. Fig C in S3 File provides a comparison of EM core, k-ecoli74, and experimental ranges for those 26 parameters.

The results indicate that while the regressed elementary kinetic parameters recapitulate training data well and are able to reasonably predict metabolite yield under conditions not used for parameterization, the $K_{m}$ parameters generated using elementary decomposition kinetics and a core metabolic network are not always consistent with experimentally determined values. For 46% of k-ecoli74 and 54% of EM core $K_{m}$ parameters compared in Fig C in S3 File, and 63% of k-ecoli74 $K_{m}$ parameters compared with experimental ranges overall, the k-ecoli74 parameter range was lower than the experimental range. Furthermore, the values k-ecoli74 and EM core [5] lumped kinetic parameters are inconsistent. Thus, the set lumped kinetic parameters capable of predicting metabolic phenotype is not unique.

1. Ott LR, Longnecker M. An Introduction to Statistical Methods and Data Analysis. 6 ed: Cengage Learning; 2010.

2. Long CP, Antoniewicz MR. Systems-level analysis of metabolic flux responses to deletion of 20 core enzymes reveals flexibility and limits of E. coli metabolism. submitted.

3. Long CP, Gonzalez JE, Feist AM, Palsson BO, Antoniewicz MR. Dissecting the genetic and metabolic mechanisms of adaptation to the knockout of a major metabolic enzyme in Escherichia coli. Proc Natl Acad Sci U S A. 2018;115(1):222-7.

4. King EL, Altman C. A schematic method of driving the rate laws for enzyme catalyzed reactions. J Phys Chem. 1956;60(10):1375-8.

5. Khodayari A, Zomorrodi AR, Liao JC, Maranas CD. A kinetic model of Escherichia coli core metabolism satisfying multiple sets of mutant flux data. Metab Eng. 2014;25:50-62.

6. Ishii N, Nakahigashi K, Baba T, Robert M, Soga T, Kanai A, et al. Multiple high-throughput analyses monitor the response of E. coli to perturbations. Science. 2007;316(5824):593-7.
