# Supplementary material for: From Escherichia coli mutant 13C labeling data to a core kinetic model: A kinetic model parameterization pipeline
Source: PLoS Comput Biol. 2019 Sep 10;15(9):e1007319. doi: 10.1371/journal.pcbi.1007319 (PMC6759195; doi:10.1371/journal.pcbi.1007319)

**S3 File: Supplementary Figures**

**Fig A. Comparison of mutant strain central carbon metabolism flux distributions with wild-type flux distribution (units: mmol/100 mmol wild-type glucose uptake).**

(A) *Δpgi* and *Δrpe* flux distributions (B) *Δzwf* and *Δgnd* flux distributions (C) *Δeda* and *Δedd* flux distributions and (D) *Δfbp* flux distributions, reaction and metabolite abbreviations provided in Supplementary File S4.

**
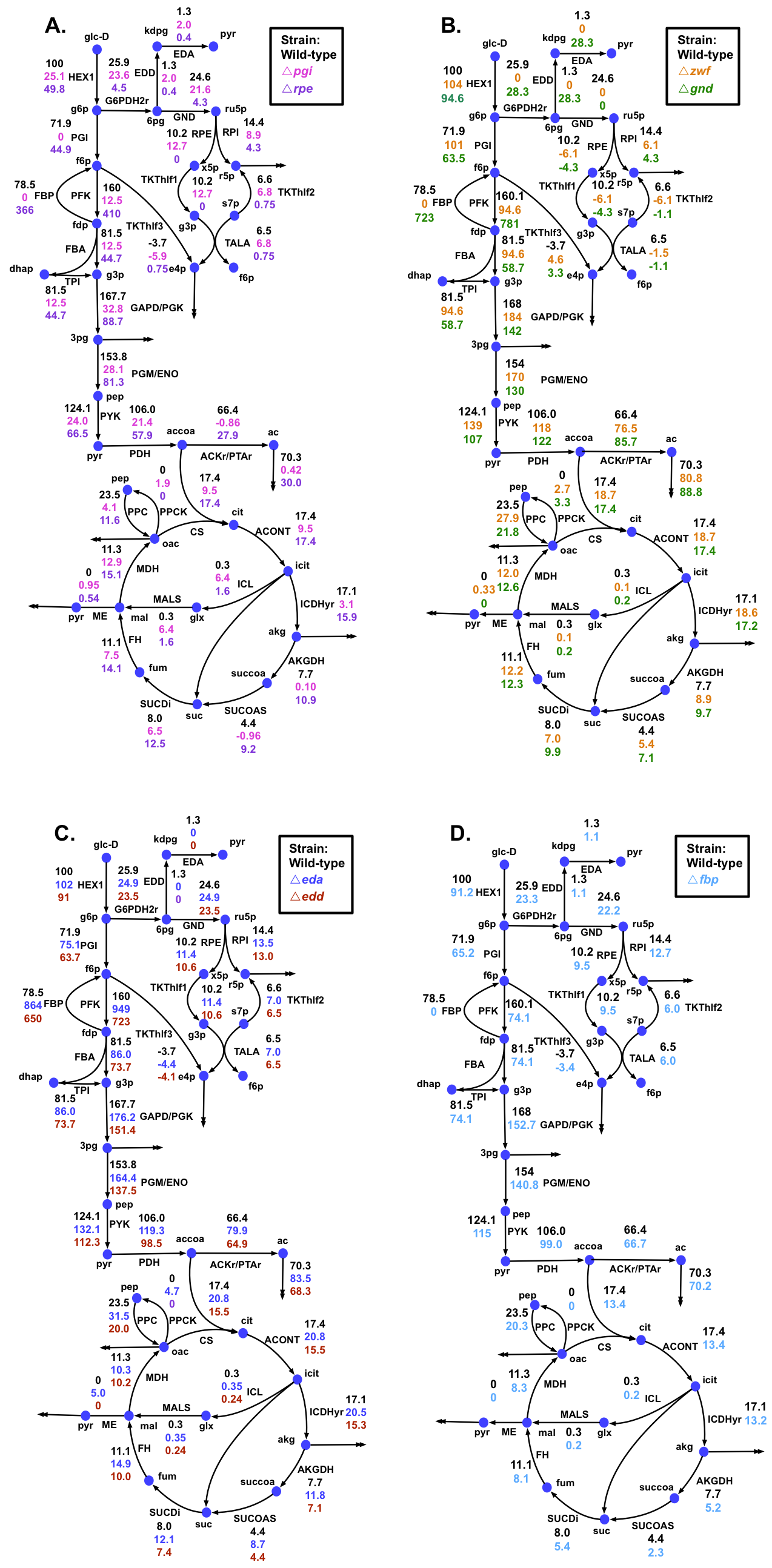
**

**Fig B. Average summed upper and lower bound range expansion.**

(A) $K_{m}$ range expansions when all models with SSR within 10% of optimal value are considered in range estimation compared to the optimal model (B) *Vmax* range expansions when all models with SSR within 10% of optimal value are considered in range estimation compared to the optimal model


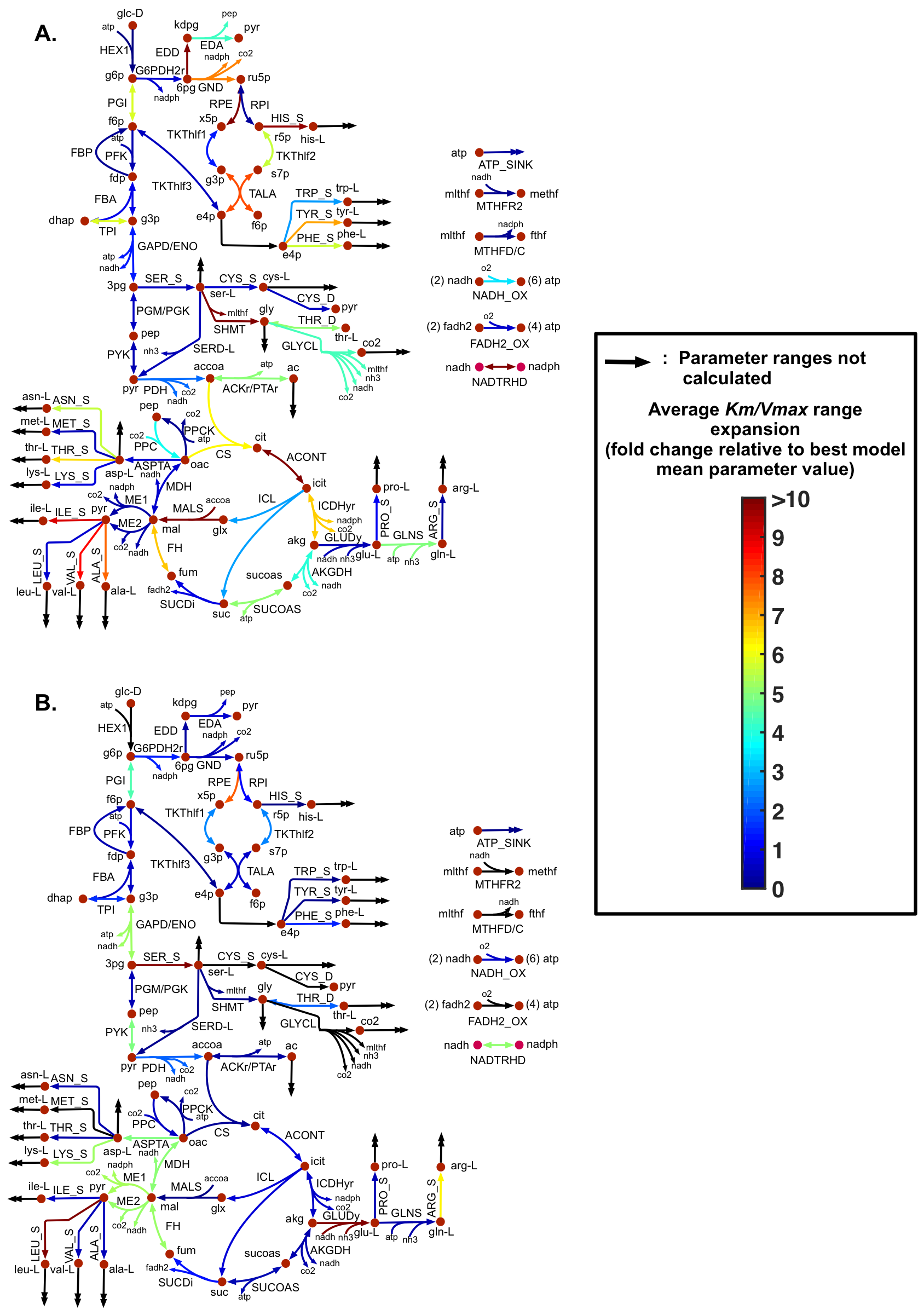


**Fig C. Comparison of k-ecoli74 and EM core Km ranges with experimentally determined ranges for 26 parameters with metabolomics data require for scaling both k-ecoli457 and k-ecoli74 parameters. Maximum scaled parameter value in image was truncated to 15, see supplementary results for full ranges.**


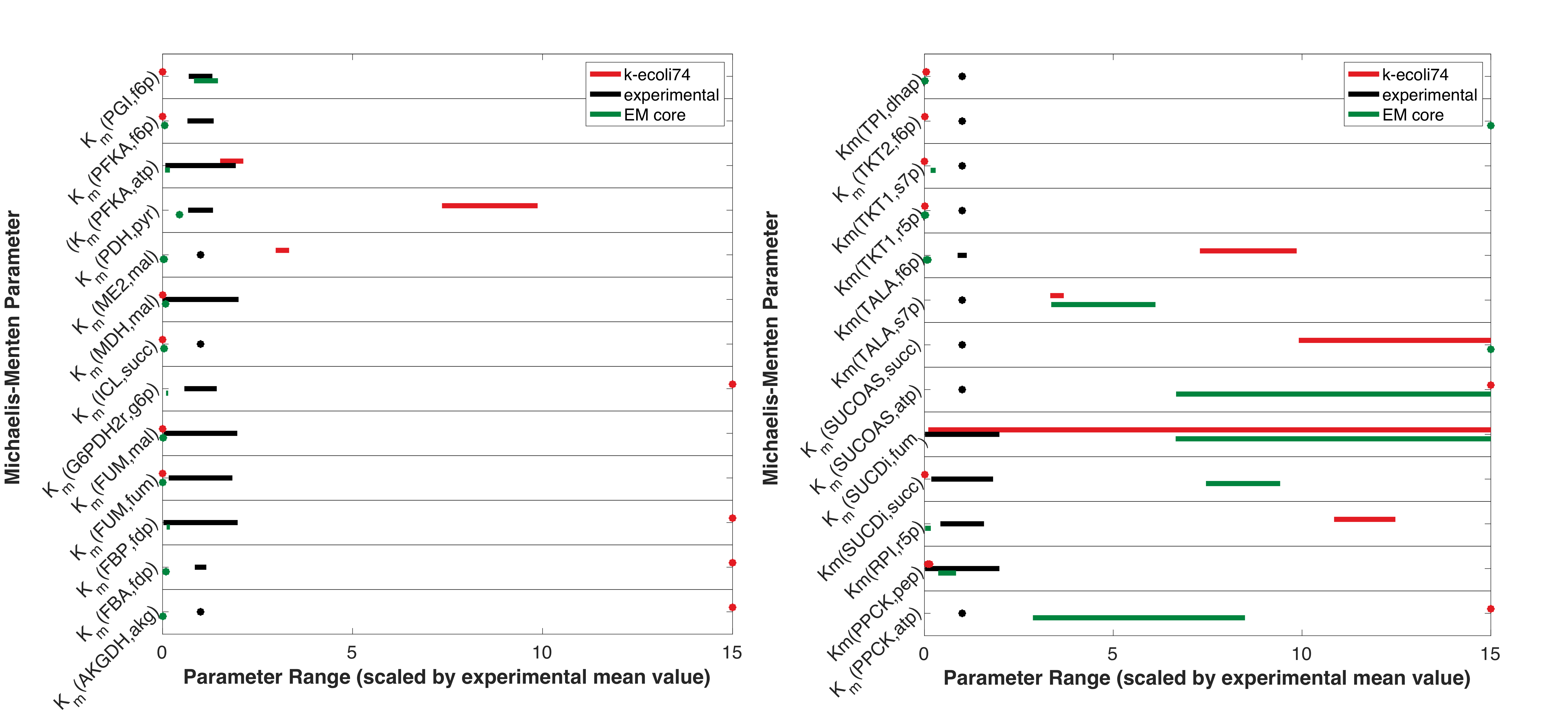


**Fig D. Genetic perturbation strategies for engineered overproducing strains.**

(A) 0.3x PTAr, 10x PPC (B) 0.1x RPI (C) 0.1x THRD-L (D) ΔSUCOAS, 0.1x FUM (E) ΔMDH, 0.1x SUCOAS (F) 0.1x ACKr (G) ΔACKr (H) 5x PYK (I) 5x NADTHRD


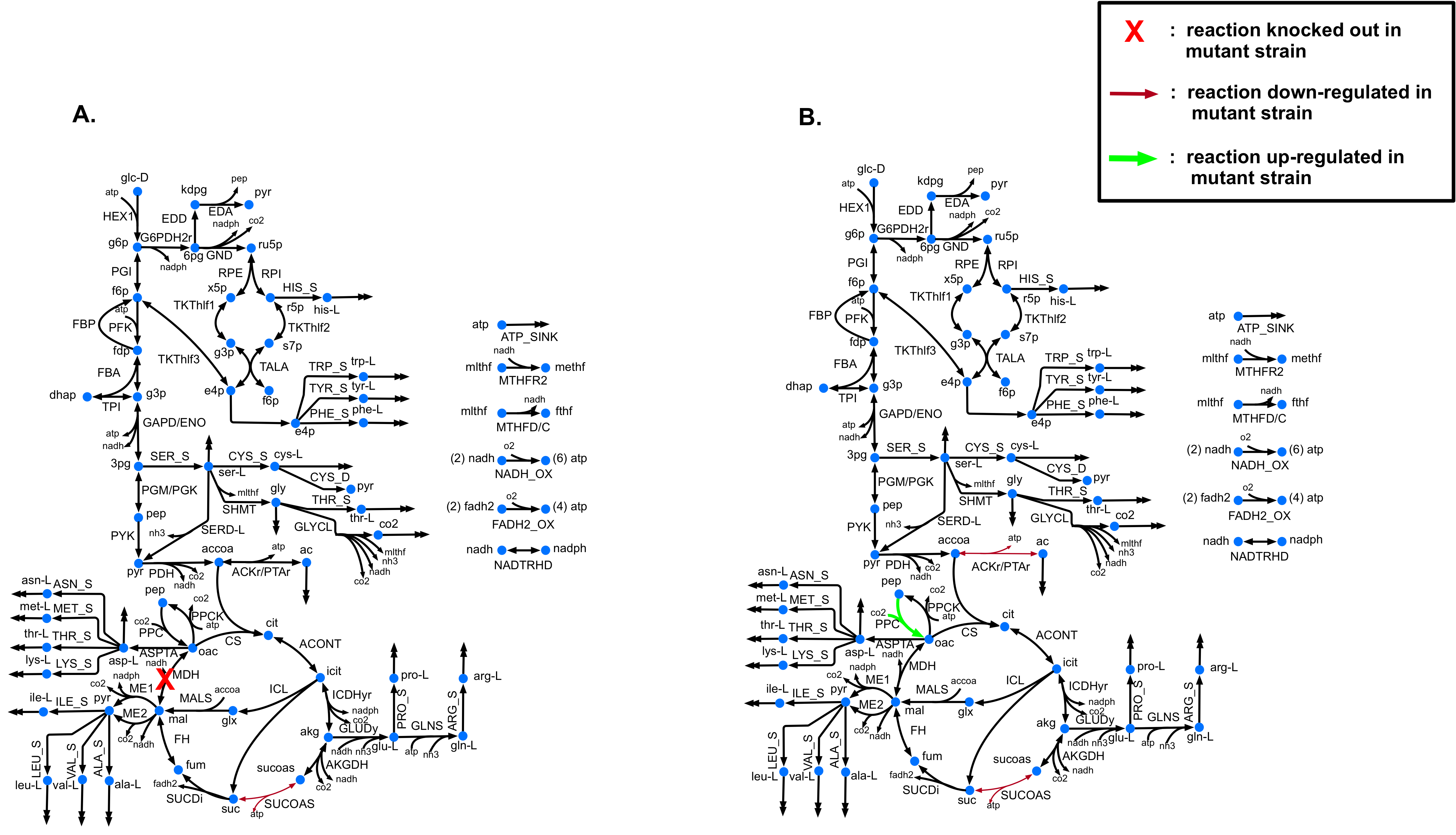


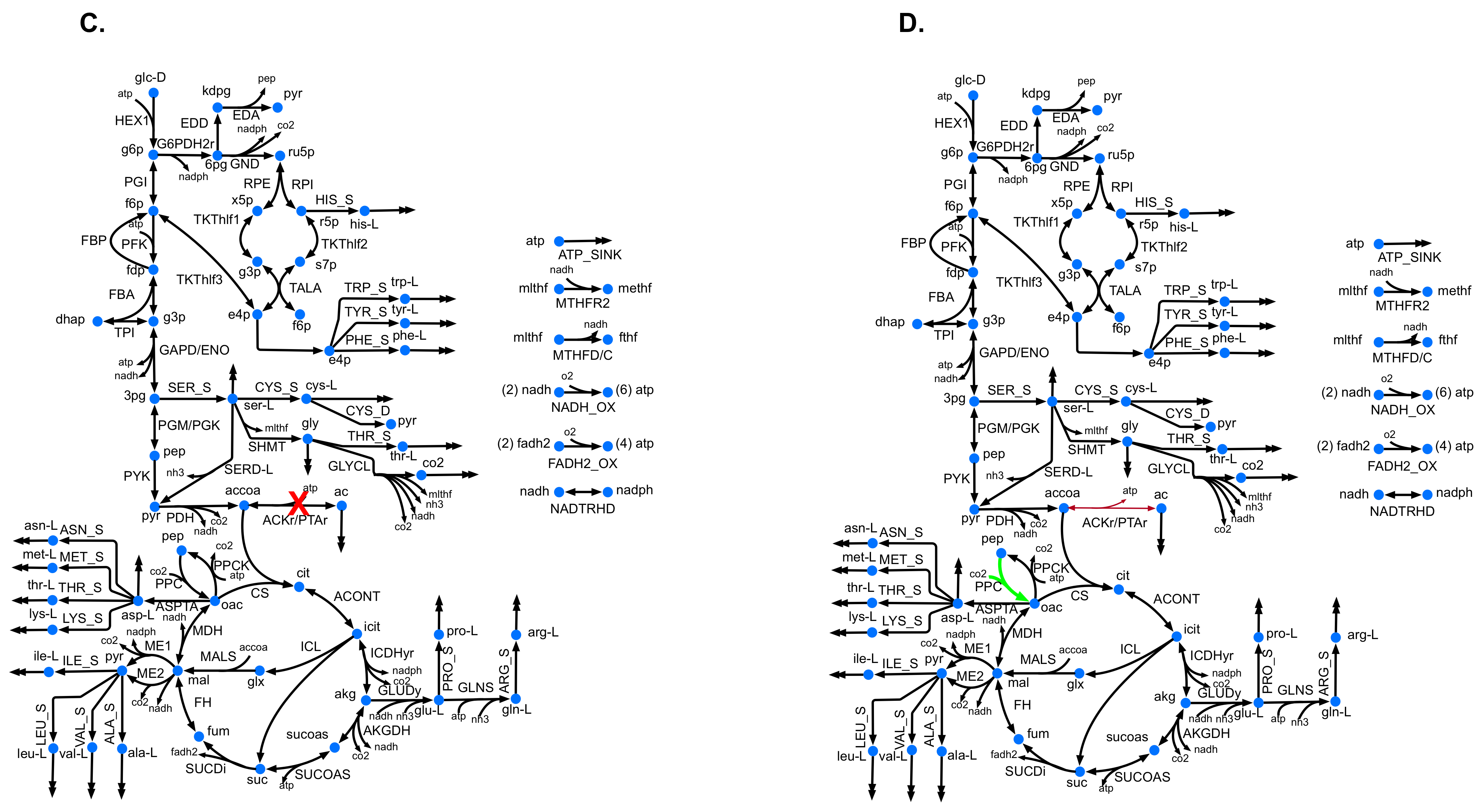


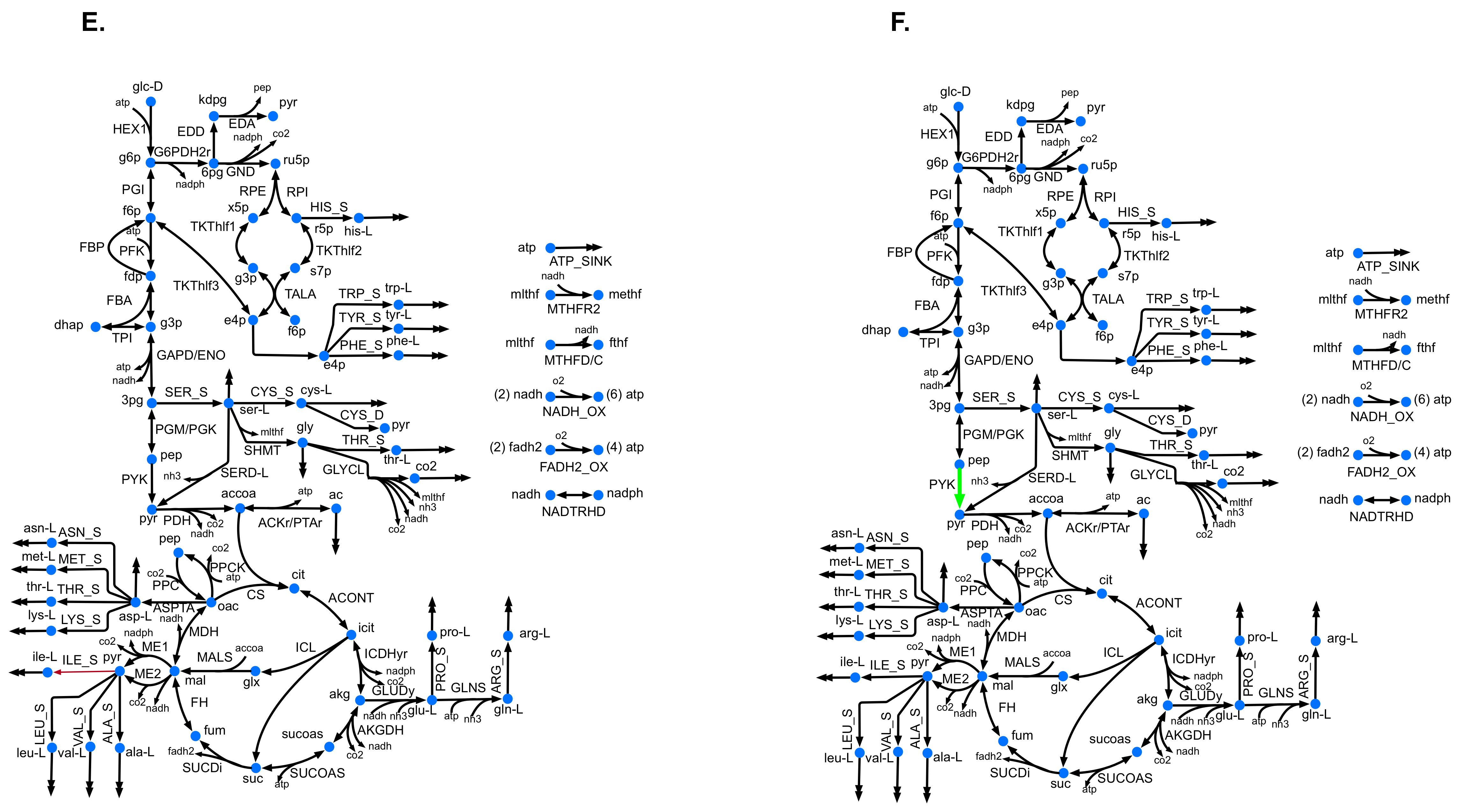


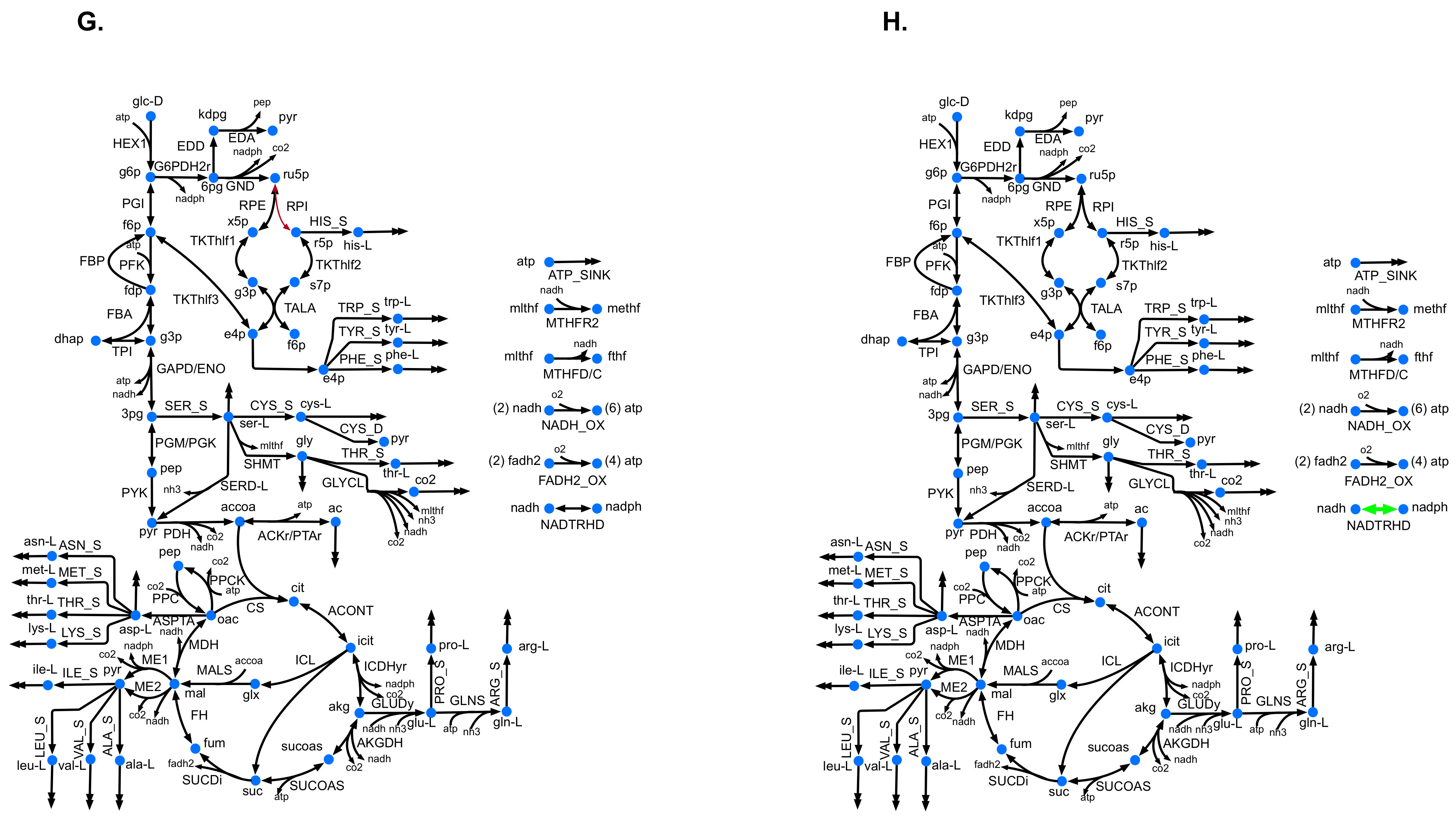


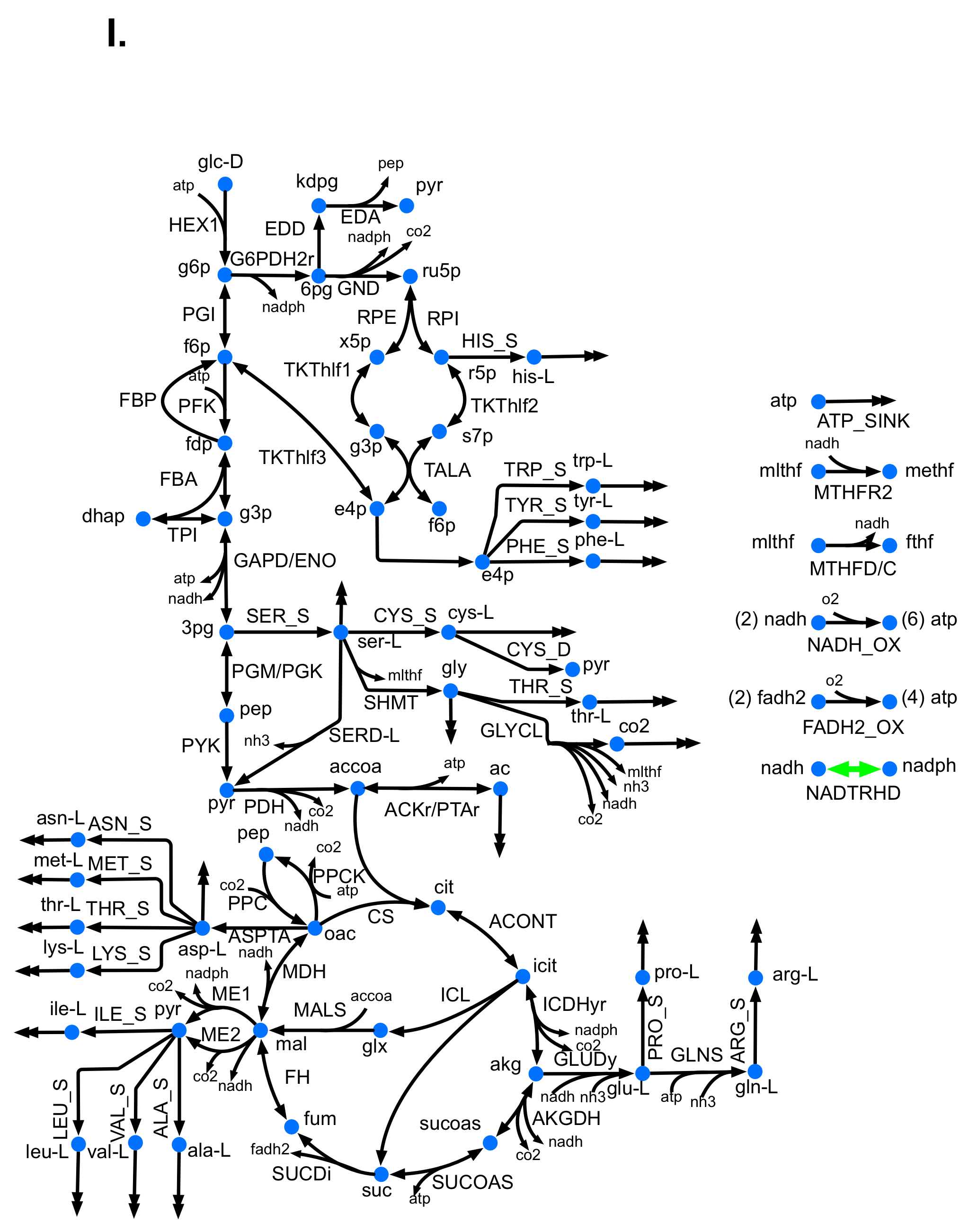


**Fig E. Relative contribution of weighted square residual of each reaction in each strain to sum of square variance weighted residual error**

(A) *Δpgi* (B) *Δrpe* (C) *Δedd* (D) *Δeda* (E) *Δfbp* (F) *Δzwf* (G) *Δgnd*





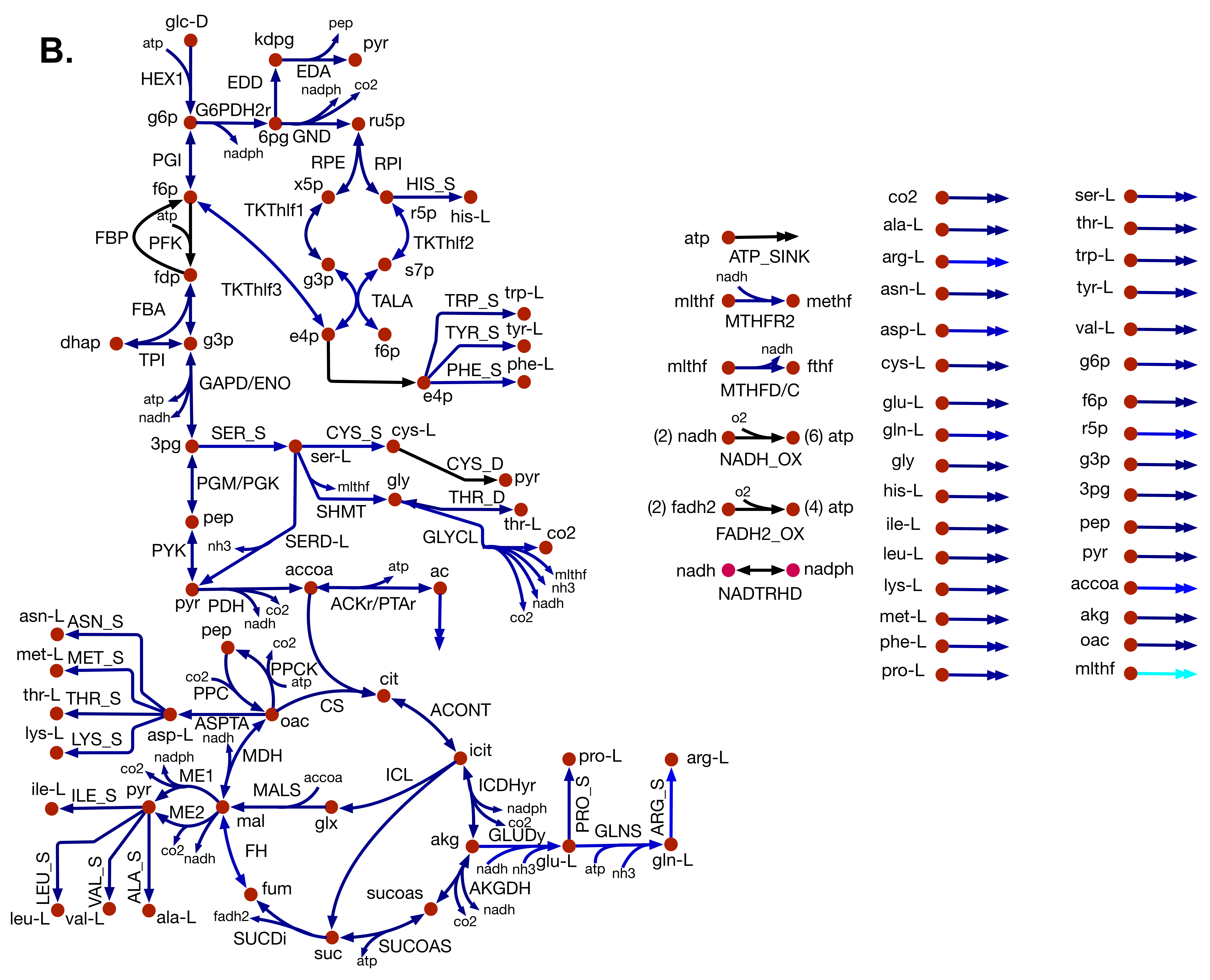


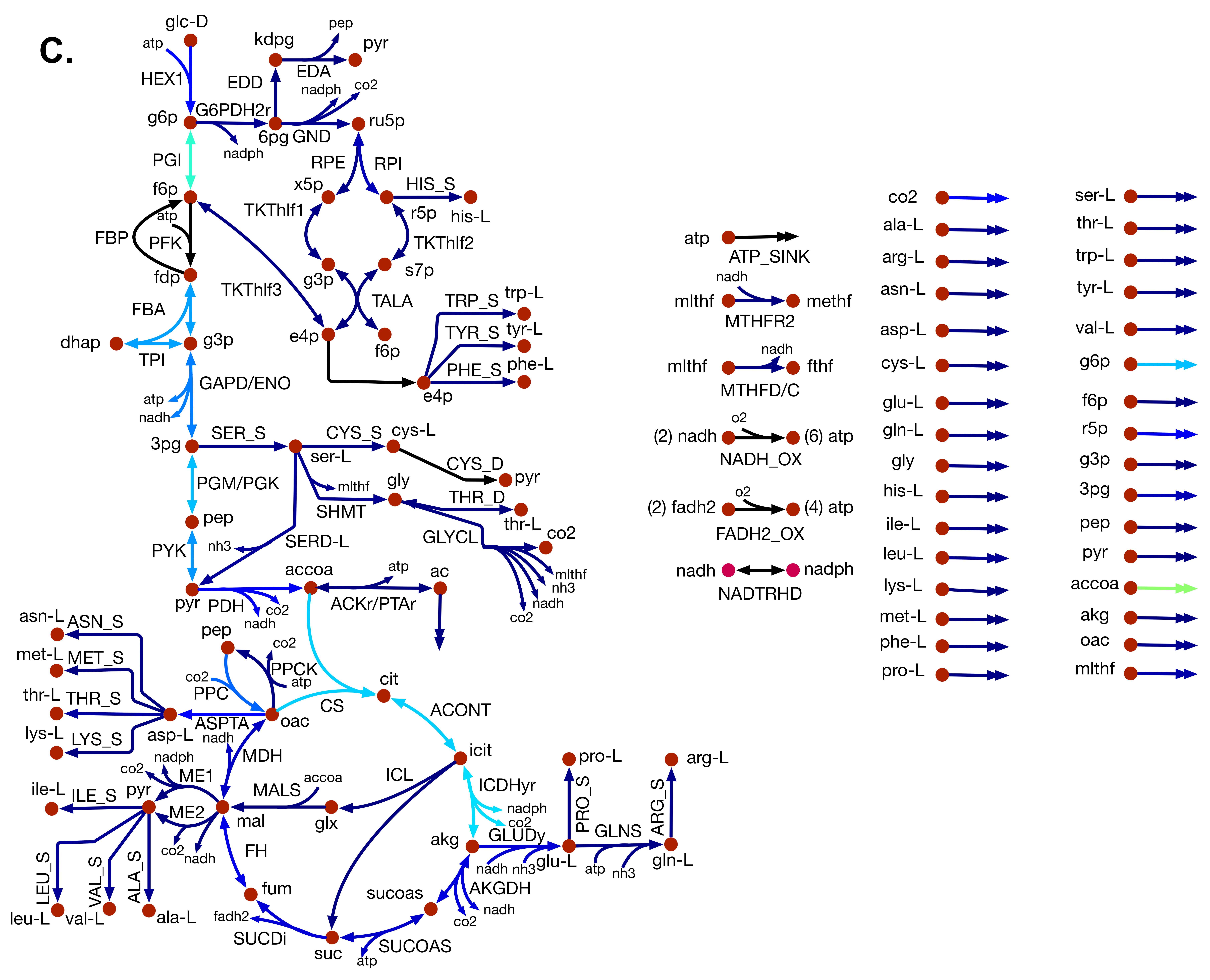


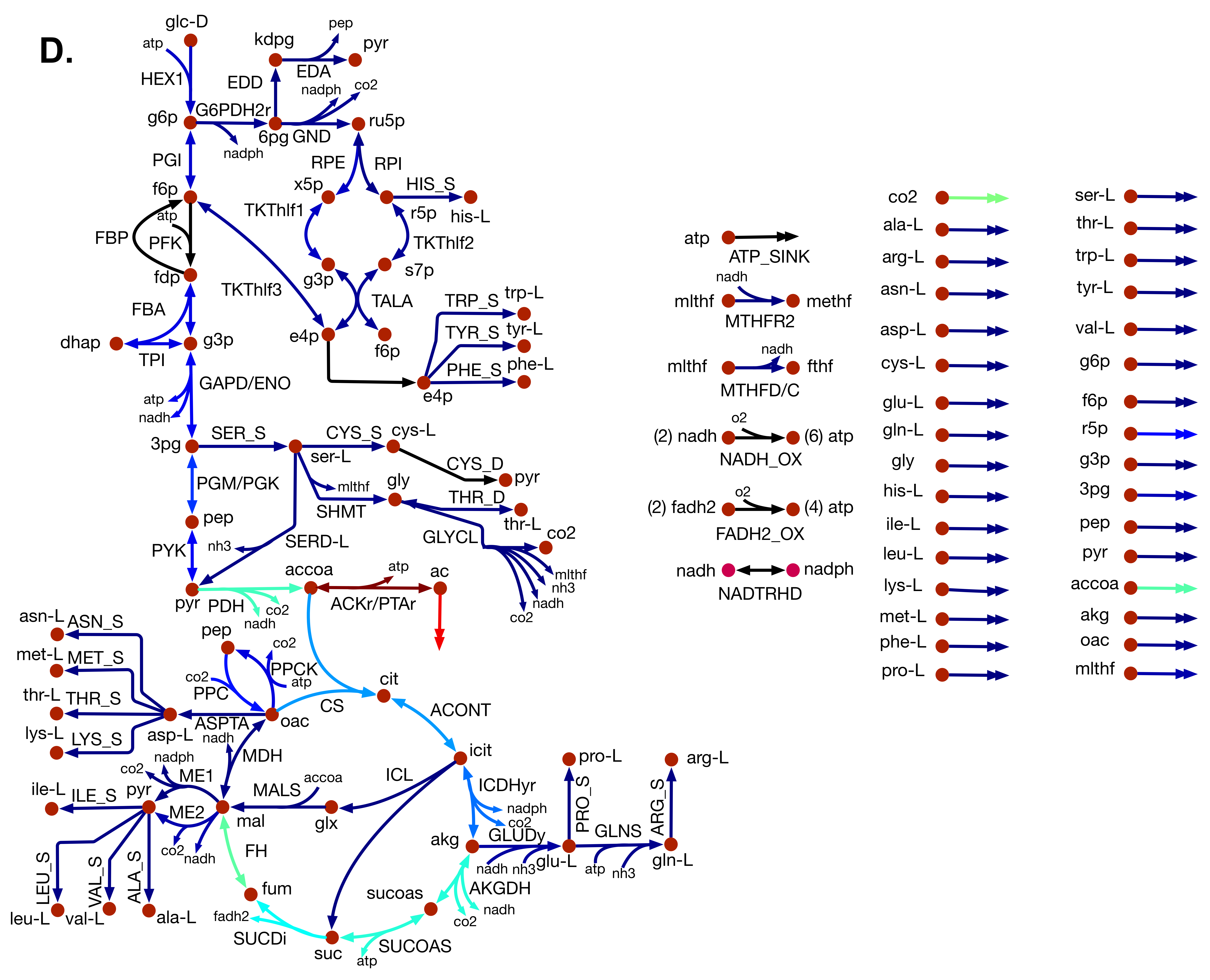


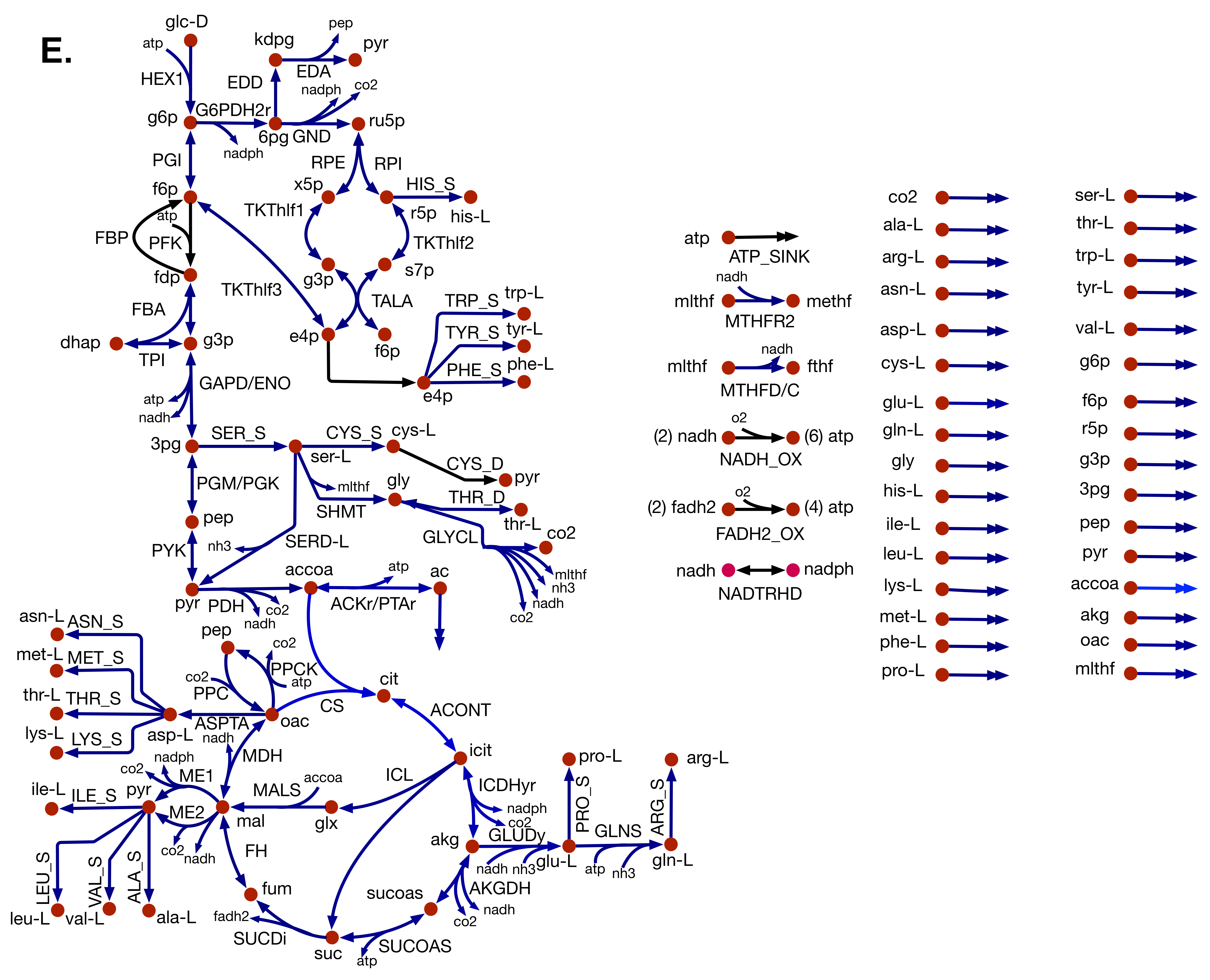


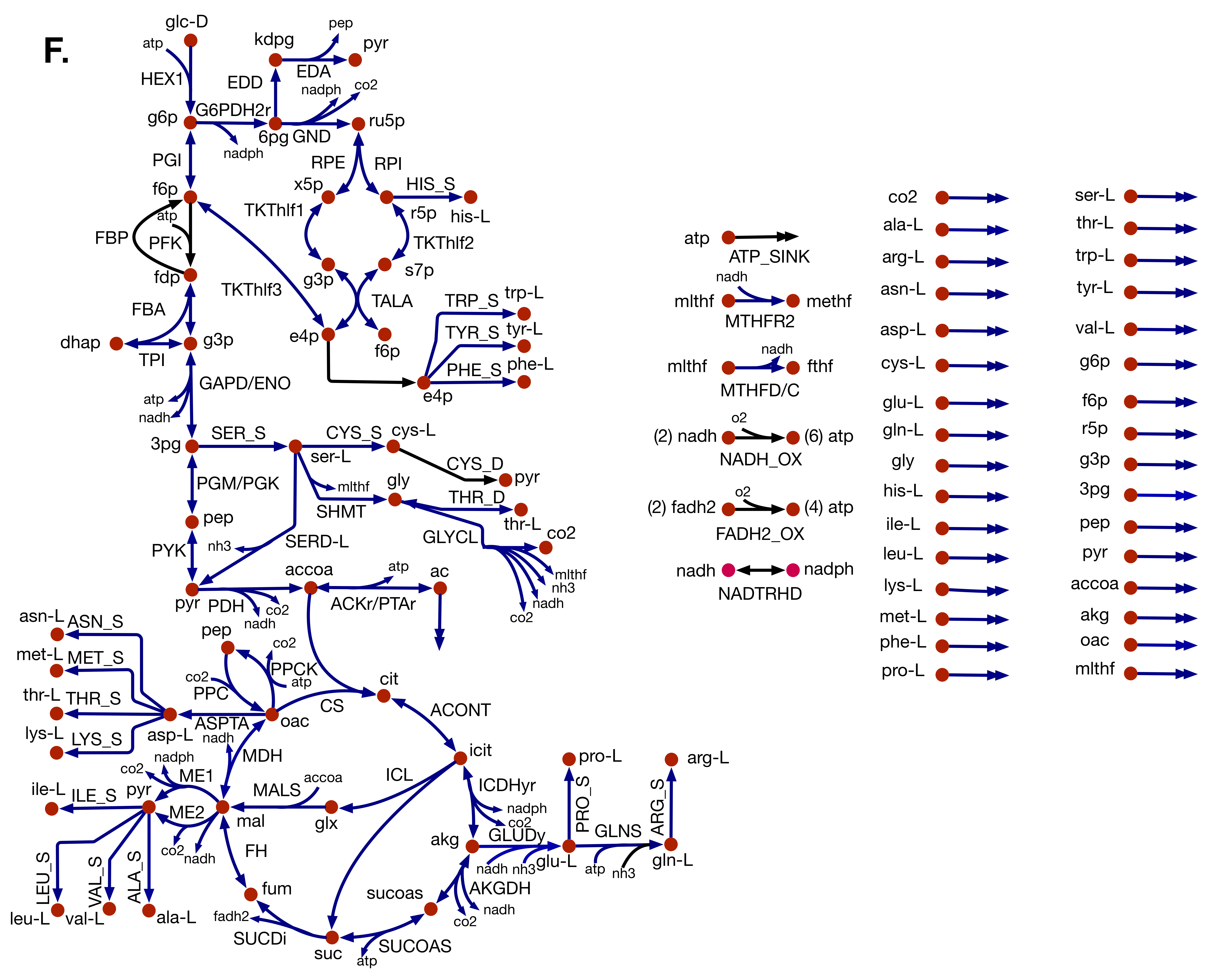


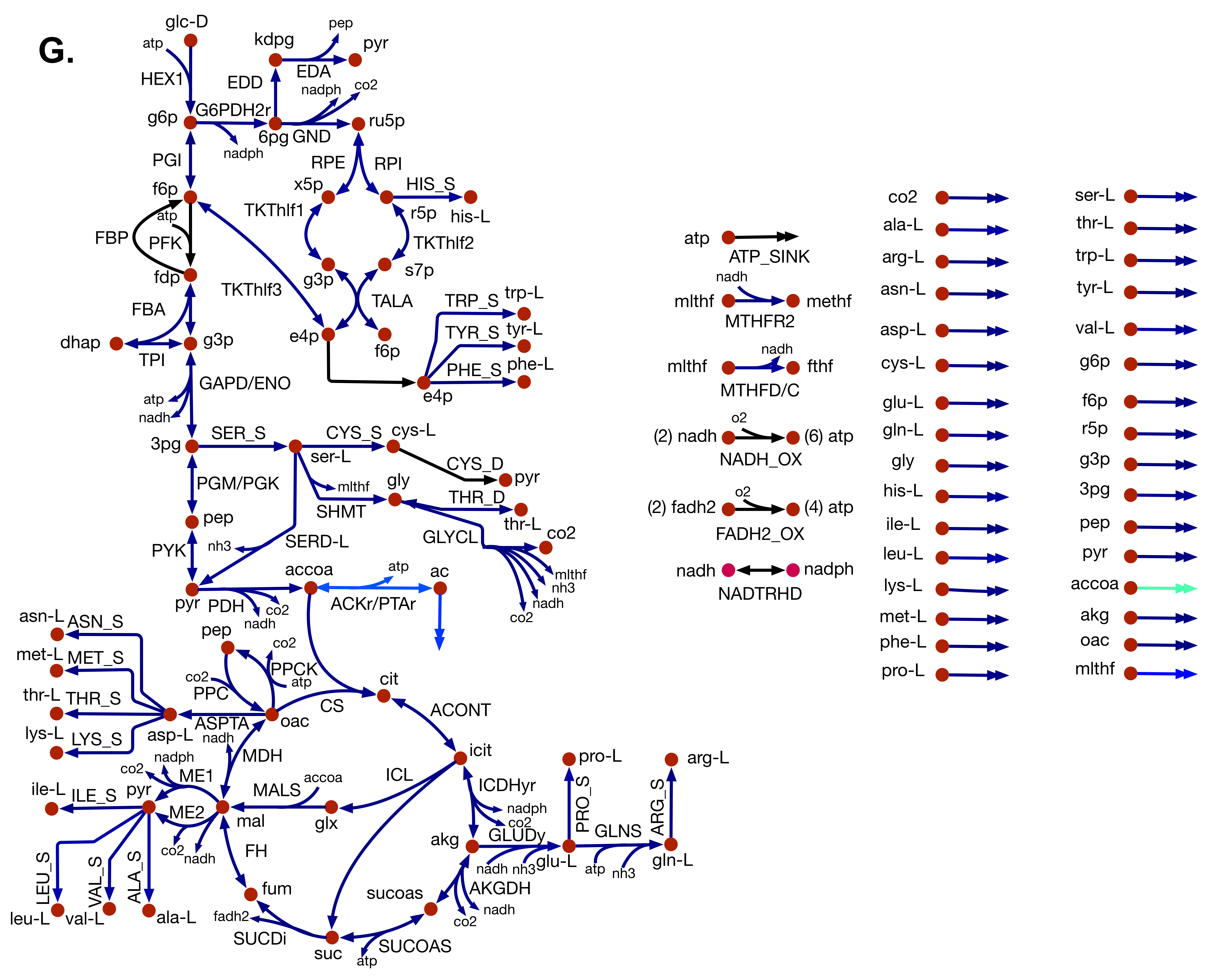

Supplement: S3 File — (DOCX) [file pcbi.1007319.s003.docx]
