# Supplementary material for: From Escherichia coli mutant 13C labeling data to a core kinetic model: A kinetic model parameterization pipeline
Source: PLoS Comput Biol. 2019 Sep 10;15(9):e1007319. doi: 10.1371/journal.pcbi.1007319 (PMC6759195; doi:10.1371/journal.pcbi.1007319)
Supplement: S5 File — (DOCX) [file pcbi.1007319.s005.docx]

**S5 File:** $\boldsymbol{K}_{\boldsymbol{m}}$ **rate constants expressed in terms of elementary kinetic parameters for central carbon method for central carbon reactions in k-ecoli74**

| Reaction | Parameter |
| --- | --- |
| PGI | $K_{m}\left( g6p \right)= \frac{k_{2}k_{4}+k_{2}k_{5}+k_{3}k_{5}}{k_{1}k_{3}+k_{1}k_{4}+k_{1}k_{5}}$ |
|  | $K_{m}\left( f6p \right)= \frac{k_{2}k_{4}+k_{2}k_{5}+k_{3}k_{5}}{k_{2}k_{6}+k_{3}k_{6}+k_{4}k_{6}}$ |
|  | $kI\left( pep \right)= {kI}_{1}$ |
|  | $kI\left( 6pg \right)= {kI}_{2}$ |
|  | $K_{m}\left( f6p,6pg \right)= \frac{{{kI}_{3}k}_{2}k_{4}+k_{2}k_{5}+k_{3}k_{5}}{k_{4}k_{6}}$ |
|  | $K_{m}\left( g6p,6pg \right)= \frac{{{kI}_{3}k}_{2}k_{4}+k_{2}k_{5}+k_{3}k_{5}}{k_{1}k_{4}+k_{1}k_{5}}$ |
| PFK | $K_{m}\left( f6p \right)= \frac{k_{2}k_{4}k_{6}+k_{2}k_{4}k_{7}+k_{2}k_{5}k_{7}}{k_{1}k_{4}k_{6}+k_{1}k_{4}k_{7}+k_{1}k_{5}k_{7}}$ |
|  | $K_{m}\left( atp \right)= \frac{k_{2}k_{4}k_{6}+k_{2}k_{4}k_{7}+k_{2}k_{5}k_{7}}{k_{3}k_{5}k_{7}}$ |
|  | $K_{m}\left( fdp \right)= \frac{k_{2}k_{4}k_{6}+k_{2}k_{4}k_{7}+k_{2}k_{5}k_{7}}{k_{2}k_{4}k_{8}+k_{2}k_{5}k_{8}+k_{2}k_{6}k_{8}+k_{4}k_{6}k_{8}}$ |
|  | $kI\left( atp \right)= {kI}_{1}$ |
|  | $kI\left( pep \right)= {kI}_{2}$ |
|  | $kI\left( f6p \right)= {kI}_{4}$ |
|  | $K_{m}\left( f6p,atp \right)= \frac{k_{2}k_{4}k_{6}+k_{2}k_{4}k_{7}+k_{2}k_{5}k_{7}}{k_{1}k_{3}k_{5}+k_{1}k_{3}k_{6}+k_{1}k_{3}k_{7}}$ |
|  | $K_{m}\left( f6p,pep \right)= \frac{{kI}_{3}(k_{2}k_{4}k_{6}+k_{2}k_{4}k_{7}+k_{2}k_{5}k_{7})}{k_{1}k_{4}k_{6}+k_{1}k_{4}k_{7}+k_{1}k_{5}k_{7}}$ |
|  | $K_{m}\left( f6p,f6p \right)= \frac{{kI}_{5}(k_{2}k_{4}k_{6}+k_{2}k_{4}k_{7}+k_{2}k_{5}k_{7})}{k_{1}k_{4}k_{6}+k_{1}k_{4}k_{7}+k_{1}k_{5}k_{7}}$ |
|  | $K_{m}\left( atp,fdp \right)= \frac{k_{2}k_{4}k_{6}+k_{2}k_{4}k_{7}+k_{2}k_{5}k_{7}}{k_{3}k_{5}k_{8}+k_{3}k_{6}k_{8}}$ |
|  | $K_{m}\left( atp,atp \right)= \frac{{kI}_{1}(k_{2}k_{4}k_{6}+k_{2}k_{4}k_{7}+k_{2}k_{5}k_{7})}{k_{3}k_{5}k_{7}}$ |
|  | $K_{m}\left( atp,pep \right)= \frac{{kI}_{2}(k_{2}k_{4}k_{6}+k_{2}k_{4}k_{7}+k_{2}k_{5}k_{7})}{k_{3}k_{5}k_{7}}$ |
|  | $K_{m}\left( atp,f6p \right)= \frac{{kI}_{4}(k_{2}k_{4}k_{6}+k_{2}k_{4}k_{7}+k_{2}k_{5}k_{7})}{k_{3}k_{5}k_{7}}$ |
|  | $K_{m}\left( fdp,pep \right)= \frac{{kI}_{3}(k_{2}k_{4}k_{6}+k_{2}k_{4}k_{7}+k_{2}k_{5}k_{7})}{k_{4}k_{6}k_{8}}$ |
|  | $K_{m}\left( fdp,f6p \right)= \frac{{kI}_{5}(k_{2}k_{4}k_{6}+k_{2}k_{4}k_{7}+k_{2}k_{5}k_{7})}{k_{4}k_{6}k_{8}}$ |
| FBP | $K_{m}\left( fdp \right)= \frac{k_{2}k_{4}+k_{2}k_{5}+k_{3}k_{5}}{k_{1}k_{3}+k_{1}k_{4}+k_{1}k_{5}}$ |
|  | $K_{m}\left( f6p \right)= \frac{k_{2}k_{4}+k_{2}k_{5}+k_{3}k_{5}}{k_{2}k_{6}+k_{3}k_{6}+k_{4}k_{6}}$ |
|  | $kI\left( fdp \right)= {kI}_{1}$ |
|  | $kI\left( pep \right)= {kI}_{2}$ |
|  | $kI\left( g6p \right)= {kI}_{4}$ |
|  | $K_{m}\left( f6p,g6p \right)= \frac{{kI}_{5}(k_{2}k_{4}+k_{2}k_{5}+k_{3}k_{5})}{k_{4}k_{6}}$ |
|  | $K_{m}\left( f6p,pep \right)= \frac{{kI}_{3}(k_{2}k_{4}+k_{2}k_{5}+k_{3}k_{5})}{k_{4}k_{6}}$ |
|  | $K_{m}\left( fdp,g6p \right)= \frac{{kI}_{5}(k_{2}k_{4}+k_{2}k_{5}+k_{3}k_{5})}{k_{1}k_{4}+k_{1}k_{5}}$ |
|  | $K_{m}\left( fdp,pep \right)= \frac{{kI}_{3}(k_{2}k_{4}+k_{2}k_{5}+k_{3}k_{5})}{k_{1}k_{4}+k_{1}k_{5}}$ |
| FBA | $K_{m}\left( fdp \right)= \frac{k_{2}k_{4}k_{7}+k_{2}k_{5}k_{7}+k_{3}k_{5}k_{7}}{k_{1}k_{3}k_{5}+k_{1}k_{3}k_{7}+k_{1}k_{4}k_{7}+k_{1}k_{5}k_{7}}$ |
|  | $K_{m}\left( g3p \right)= \frac{k_{2}k_{4}k_{7}+k_{2}k_{5}k_{7}+k_{3}k_{5}k_{7}}{k_{2}k_{4}k_{8}+k_{2}k_{5}k_{8}+k_{3}k_{5}k_{8}}$ |
|  | $K_{m}\left( dhap \right)= \frac{k_{2}k_{4}k_{7}+k_{2}k_{5}k_{7}+k_{3}k_{5}k_{7}}{k_{2}k_{4}k_{6}}$ |
|  | $kI\left( 3pg \right)= {kI}_{1}$ |
|  | $kI\left( cys \right)= {kI}_{2}$ |
|  | $kI\left( dhap \right)= {kI}_{3}$ |
|  | $kI\left( g3p \right)= {kI}_{5}$ |
|  | $K_{m}^{1}\left( fdp,dhap \right)= \frac{k_{2}k_{4}k_{7}+k_{2}k_{5}k_{7}+k_{3}k_{5}k_{7}}{k_{1}k_{3}k_{6}+k_{1}k_{4}k_{6}}$ |
|  | $K_{m}^{2}\left( fdp,dhap \right)= \frac{{kI}_{4}(k_{2}k_{4}+k_{2}k_{5}+k_{3}k_{5})}{k_{1}k_{4}+k_{1}k_{5}}$ |
|  | $K_{m}\left( fdp,g3p \right)= \frac{{kI}_{6}(k_{2}k_{4}+k_{2}k_{5}+k_{3}k_{5})}{k_{1}k_{4}+k_{1}k_{5}}$ |
|  | $K_{m}\left( g3p,dhap \right)= \frac{k_{2}k_{4}k_{7}+k_{2}k_{5}k_{7}+k_{3}k_{5}k_{7}}{k_{2}k_{6}k_{8}+k_{3}k_{6}k_{8}+k_{4}k_{6}k_{8}}$ |
|  | $K_{m}\left( dhap,3pg \right)= \frac{{kI}_{1}(k_{2}k_{4}k_{7}+k_{2}k_{5}k_{7}+k_{3}k_{5}k_{7})}{k_{2}k_{4}k_{6}}$ |
|  | $K_{m}\left( dhap,cys \right)= \frac{{kI}_{2}(k_{2}k_{4}k_{7}+k_{2}k_{5}k_{7}+k_{3}k_{5}k_{7})}{k_{2}k_{4}k_{6}}$ |
|  | $K_{m}\left( dhap,dhap \right)= \frac{{kI}_{3}(k_{2}k_{4}k_{7}+k_{2}k_{5}k_{7}+k_{3}k_{5}k_{7})}{k_{2}k_{4}k_{6}}$ |
|  | $K_{m}\left( dhap,g3p \right)= \frac{{kI}_{5}(k_{2}k_{4}k_{7}+k_{2}k_{5}k_{7}+k_{3}k_{5}k_{7})}{k_{2}k_{4}k_{6}}$ |
|  | $K_{m}\left( fdp,dhap,dhap \right)= \frac{{kI}_{4}(k_{2}k_{4}k_{7}+k_{2}k_{5}k_{7}+k_{3}k_{5}k_{7})}{k_{1}k_{4}k_{6}}$ |
|  | $K_{m}\left( fdp,dhap,g3p \right)= \frac{{kI}_{6}(k_{2}k_{4}k_{7}+k_{2}k_{5}k_{7}+k_{3}k_{5}k_{7})}{k_{1}k_{4}k_{6}}$ |
|  | $K_{m}\left( g3p,dhap,g3p \right)= \frac{{kI}_{6}(k_{2}k_{4}k_{7}+k_{2}k_{5}k_{7}+k_{3}k_{5}k_{7})}{k_{4}k_{6}k_{8}}$ |
|  | $K_{m}\left( g3p,dhap,dhap \right)= \frac{{kI}_{4}(k_{2}k_{4}k_{7}+k_{2}k_{5}k_{7}+k_{3}k_{5}k_{7})}{k_{4}k_{6}k_{8}}$ |
| TPI | $K_{m}\left( dhap \right)= \frac{k_{2}k_{4}+k_{2}k_{5}+k_{3}k_{5}}{k_{1}k_{3}+k_{1}k_{4}+k_{1}k_{5}}$ |
|  | $K_{m}\left( g3p \right)= \frac{k_{2}k_{4}+k_{2}k_{5}+k_{3}k_{5}}{k_{2}k_{6}+k_{3}k_{6}+k_{4}k_{6}}$ |
| GAPD/PGK | $K_{m}\left( g3p \right)= \frac{k_{2}k_{4}k_{7}k_{9}+k_{2}k_{5}k_{7}k_{9}+k_{3}k_{5}k_{7}k_{9}}{k_{1}k_{3}k_{5}k_{7}+k_{1}k_{3}k_{5}k_{9}+k_{1}k_{3}k_{7}k_{9}+k_{1}k_{4}k_{7}k_{9}+k_{1}k_{5}k_{7}k_{9}}$ |
|  | $K_{m}\left( nadh \right)= \frac{k_{2}k_{4}k_{9}+k_{2}k_{5}k_{9}+k_{3}k_{5}k_{9}}{k_{2}k_{4}k_{10}+k_{2}k_{5}k_{10}+k_{3}k_{5}k_{10}}$ |
|  | $K_{m}\left( atp \right)= \frac{k_{2}k_{4}k_{7}+k_{2}k_{5}k_{7}+k_{3}k_{5}k_{7}}{k_{2}k_{4}k_{6}}$ |
|  | $kI\left( atp \right)= {kI}_{1}$ |
|  | $K_{m}\left( atp,atp \right)= \frac{{kI}_{1}(k_{2}k_{4}k_{7}+k_{2}k_{5}k_{7}+k_{3}k_{5}k_{7})}{k_{2}k_{4}k_{6}}$ |
|  | $K_{m}\left( 3pg,atp \right)= \frac{k_{2}k_{4}k_{7}k_{9}+k_{2}k_{5}k_{7}k_{9}+k_{3}k_{5}k_{7}k_{9}}{k_{2}k_{4}k_{6}k_{8}}$ |
|  | $K_{m}\left( nadh,atp \right)= \frac{k_{2}k_{4}k_{7}k_{9}+k_{2}k_{5}k_{7}k_{9}+k_{3}k_{5}k_{7}k_{9}}{k_{2}k_{4}k_{6}k_{10}}$ |
|  | $K_{m}\left( nadh,3pg \right)= \frac{k_{2}k_{4}k_{7}k_{9}+k_{2}k_{5}k_{7}k_{9}+k_{3}k_{5}k_{7}k_{9}}{k_{2}k_{4}k_{8}k_{10}+k_{2}k_{5}k_{8}k_{10}+k_{3}k_{5}k_{8}k_{10}}$ |
|  | $K_{m}\left( g3p,atp \right)= \frac{{kI}_{2}(k_{2}k_{4}+k_{2}k_{5}+k_{3}k_{5})}{k_{1}k_{4}+k_{1}k_{5}}$ |
|  | $K_{m}\left( g3p,atp \right)= \frac{k_{2}k_{4}k_{7}+k_{2}k_{5}k_{7}+k_{3}k_{5}k_{7}}{k_{1}k_{3}k_{6}+k_{1}k_{4}k_{6}}$ |
|  | $K_{m}\left( g3p,atp \right)= \frac{k_{2}k_{4}k_{7}+k_{2}k_{5}k_{7}+k_{3}k_{5}k_{7}}{k_{1}k_{3}k_{6}+k_{1}k_{4}k_{6}}$ |
|  | $K_{m}\left( 3pg,atp,atp \right)= \frac{{kI}_{1}(k_{2}k_{4}k_{7}k_{9}+k_{2}k_{5}k_{7}k_{9}+k_{3}k_{5}k_{7}k_{9})}{k_{2}k_{4}k_{6}k_{8}}$ |
|  | $K_{m}\left( 3pg,nadh,atp \right)= \frac{k_{2}k_{4}k_{7}k_{9}+k_{2}k_{5}k_{7}k_{9}+k_{3}k_{5}k_{7}k_{9}}{k_{2}k_{6}k_{8}k_{10}+k_{3}k_{6}k_{8}k_{10}+k_{4}k_{6}k_{8}k_{10}}$ |
|  | $K_{m}\left( g3p,atp,atp \right)= \frac{{kI}_{2}(k_{2}k_{4}k_{7}+k_{2}k_{5}k_{7}+k_{3}k_{5}k_{7})}{k_{1}k_{4}k_{6}}$ |
|  | $K_{m}\left( g3p,3pg,atp \right)= \frac{k_{2}k_{4}k_{7}k_{9}+k_{2}k_{5}k_{7}k_{9}+k_{3}k_{5}k_{7}k_{9}}{k_{1}k_{3}k_{6}k_{8}+k_{1}k_{4}k_{6}k_{8}}$ |
|  | $K_{m}\left( g3p,3pg,atp,atp \right)= \frac{{kI}_{2}(k_{2}k_{4}k_{7}k_{9}+k_{2}k_{5}k_{7}k_{9}+k_{3}k_{5}k_{7}k_{9})}{k_{1}k_{4}k_{6}k_{8}}$ |
|  | $K_{m}\left( nadh,3pg,atp,atp \right)= \frac{{kI}_{2}(k_{2}k_{4}k_{7}k_{9}+k_{2}k_{5}k_{7}k_{9}+k_{3}k_{5}k_{7}k_{9})}{k_{4}k_{6}k_{8}k_{10}}$ |
| PGM/ENO | $K_{m}\left( 3pg \right)= \frac{k_{2}k_{4}+k_{2}k_{5}+k_{3}k_{5}}{k_{1}k_{3}+k_{1}k_{4}+k_{1}k_{5}}$ |
|  | $K_{m}\left( pep \right)= \frac{k_{2}k_{4}+k_{2}k_{5}+k_{3}k_{5}}{k_{2}k_{6}+k_{3}k_{6}+k_{4}k_{6}}$ |
| PYK | $K_{m}\left( pep \right)= \frac{k_{2}k_{4}k_{7}+k_{2}k_{5}k_{7}+k_{3}k_{5}k_{7}}{k_{1}k_{3}k_{5}+k_{1}k_{3}k_{7}+k_{1}k_{4}k_{7}+k_{1}k_{5}k_{7}}$ |
|  | $K_{m}\left( pyr \right)= \frac{k_{2}k_{4}k_{7}+k_{2}k_{5}k_{7}+k_{3}k_{5}k_{7}}{k_{2}k_{4}k_{8}+k_{2}k_{5}k_{8}+k_{3}k_{5}k_{8}}$ |
|  | $K_{m}\left( atp \right)= \frac{k_{2}k_{4}k_{7}+k_{2}k_{5}k_{7}+k_{3}k_{5}k_{7}}{k_{2}k_{4}k_{6}}$ |
|  | $kI\left( atp \right)= {kI}_{1}$ |
|  | $kI\left( succoa \right)= {kI}_{3}$ |
|  | $K_{m}\left( pyr,succoa \right)= \frac{{kI}_{3}(k_{2}k_{4}k_{7}+k_{2}k_{5}k_{7}+k_{3}k_{5}k_{7})}{k_{2}k_{4}k_{6}}$ |
|  | $K_{m}^{1}\left( pyr,atp \right)= \frac{{kI}_{1}(k_{2}k_{4}k_{7}+k_{2}k_{5}k_{7}+k_{3}k_{5}k_{7})}{k_{2}k_{4}k_{6}}$ |
|  | $K_{m}^{2}\left( pyr,atp \right)= \frac{k_{2}k_{4}k_{7}+k_{2}k_{5}k_{7}+k_{3}k_{5}k_{7}}{k_{2}k_{4}k_{6}+k_{3}k_{6}k_{8}+k_{4}k_{6}k_{8}}$ |
|  | $K_{m}\left( pep,succoa \right)= \frac{{kI}_{4}(k_{2}k_{4}+k_{2}k_{5}+k_{3}k_{5})}{k_{1}k_{4}+k_{1}k_{5}}$ |
|  | $K_{m}\left( pep,atp \right)= \frac{{kI}_{2}(k_{2}k_{4}+k_{2}k_{5}+k_{3}k_{5})}{k_{1}k_{4}+k_{1}k_{5}}$ |
|  | $K_{m}\left( pep,atp \right)= \frac{k_{2}k_{4}k_{7}+k_{2}k_{5}k_{7}+k_{3}k_{5}k_{7}}{k_{1}k_{3}k_{6}+k_{1}k_{4}k_{6}}$ |
|  | $K_{m}\left( pyr,atp,succoa \right)= \frac{{kI}_{4}(k_{2}k_{4}k_{7}+k_{2}k_{5}k_{7}+k_{3}k_{5}k_{7})}{k_{4}k_{6}k_{8}}$ |
|  | $K_{m}\left( pyr,atp,atp \right)= \frac{{kI}_{2}(k_{2}k_{4}k_{7}+k_{2}k_{5}k_{7}+k_{3}k_{5}k_{7})}{k_{4}k_{6}k_{8}}$ |
|  | $K_{m}\left( pyr,atp,atp \right)= \frac{{kI}_{2}(k_{2}k_{4}k_{7}+k_{2}k_{5}k_{7}+k_{3}k_{5}k_{7})}{k_{4}k_{6}k_{8}}$ |
|  | $K_{m}\left( pep,atp,atp \right)= \frac{{kI}_{2}(k_{2}k_{4}k_{7}+k_{2}k_{5}k_{7}+k_{3}k_{5}k_{7})}{k_{1}k_{4}k_{6}}$ |
| G6PDH2r | $K_{m}\left( g6p \right)= \frac{k_{2}k_{4}k_{7}+k_{2}k_{5}k_{7}+k_{3}k_{5}k_{7}}{k_{1}k_{3}k_{5}+k_{1}k_{3}k_{7}+k_{1}k_{4}k_{7}+k_{1}k_{5}k_{7}}$ |
|  | $K_{m}\left( nadph \right)= \frac{k_{2}k_{4}k_{7}+k_{2}k_{5}k_{7}+k_{3}k_{5}k_{7}}{k_{2}k_{4}k_{8}+k_{2}k_{5}k_{8}+k_{3}k_{5}k_{8}}$ |
|  | $K_{m}\left( 6pg \right)= \frac{k_{2}k_{4}k_{7}+k_{2}k_{5}k_{7}+k_{3}k_{5}k_{7}}{k_{2}k_{4}k_{6}}$ |
|  | $kI\left( nadh \right)= {kI}_{1}$ |
|  | $kI\left( atp \right)= {kI}_{2}$ |
|  | $kI\left( nadph \right)= {kI}_{3}$ |
|  | $K_{m}\left( g6p,6pg \right)= \frac{k_{2}k_{4}k_{7}+k_{2}k_{5}k_{7}+k_{3}k_{5}k_{7}}{k_{1}k_{3}k_{6}+k_{1}k_{4}k_{6}}$ |
|  | $K_{m}\left( g6p,nadph \right)= \frac{{kI}_{4}(k_{2}k_{4}+k_{2}k_{5}+k_{3}k_{5})}{k_{1}k_{4}+k_{1}k_{5}}$ |
|  | $K_{m}\left( nadph,6pg \right)= \frac{k_{2}k_{4}k_{7}+k_{2}k_{5}k_{7}+k_{3}k_{5}k_{7}}{k_{2}k_{4}k_{6}+k_{3}k_{6}k_{8}+k_{4}k_{6}k_{8}}$ |
|  | $K_{m}\left( 6pg,nadh \right)= \frac{{kI}_{1}{(k}_{2}k_{4}k_{7}+k_{2}k_{5}k_{7}+k_{3}k_{5}k_{7})}{k_{2}k_{4}k_{6}}$ |
|  | $K_{m}\left( 6pg,atp \right)= \frac{{kI}_{2}{(k}_{2}k_{4}k_{7}+k_{2}k_{5}k_{7}+k_{3}k_{5}k_{7})}{k_{2}k_{4}k_{6}}$ |
|  | $K_{m}\left( 6pg,nadph \right)= \frac{{kI}_{3}{(k}_{2}k_{4}k_{7}+k_{2}k_{5}k_{7}+k_{3}k_{5}k_{7})}{k_{2}k_{4}k_{6}}$ |
|  | $K_{m}\left( nadph,6pg,nadph \right)= \frac{{kI}_{4}{(k}_{2}k_{4}k_{7}+k_{2}k_{5}k_{7}+k_{3}k_{5}k_{7})}{k_{4}k_{6}k_{8}}$ |
|  | $K_{m}\left( g6p,6pg,nadph \right)= \frac{{kI}_{4}{(k}_{2}k_{4}k_{7}+k_{2}k_{5}k_{7}+k_{3}k_{5}k_{7})}{k_{1}k_{4}k_{6}}$ |
| GND | $K_{m}\left( 6pg \right)= \frac{k_{2}k_{4}k_{7}k_{9}+k_{2}k_{5}k_{7}k_{9}+k_{3}k_{5}k_{7}k_{9}}{k_{1}k_{3}k_{5}k_{7}+k_{1}k_{3}k_{5}k_{9}+k_{1}k_{3}k_{7}k_{9}+k_{1}k_{4}k_{7}k_{9}+k_{1}k_{5}k_{7}k_{9}}$ |
|  | $K_{m}\left( co2 \right)= \frac{k_{2}k_{4}k_{9}+k_{2}k_{5}k_{9}+k_{3}k_{5}k_{9}}{k_{2}k_{4}k_{10}+k_{2}k_{5}k_{10}+k_{3}k_{5}k_{10}}$ |
|  | $K_{m}\left( nadph \right)= \frac{k_{2}k_{4}k_{7}+k_{2}k_{5}k_{7}+k_{3}k_{5}k_{7}}{k_{2}k_{4}k_{6}}$ |
|  | $kI\left( atp \right)= {kI}_{1}$ |
|  | $kI\left( nadph \right)= {kI}_{2}$ |
|  | $kI\left( fdp \right)= {kI}_{3}$ |
|  | $kI\left( ru5p \right)= {kI}_{5}$ |
|  | $K_{m}\left( 6pg,ru5p \right)= \frac{k_{2}k_{4}k_{7}k_{9}+k_{2}k_{5}k_{7}k_{9}+k_{3}k_{5}k_{7}k_{9}}{k_{1}k_{3}k_{5}k_{8}}$ |
|  | $K_{m}\left( 6pg,nadph \right)= \frac{k_{2}k_{4}k_{7}+k_{2}k_{5}k_{7}+k_{3}k_{5}k_{7}}{k_{1}k_{3}k_{6}+k_{1}k_{4}k_{6}}$ |
|  | $K_{m}\left( 6pg,fdp \right)= \frac{{kI}_{4}(k_{2}k_{4}+k_{2}k_{5}+k_{3}k_{5})}{k_{1}k_{4}+k_{1}k_{5}}$ |
|  | $K_{m}\left( 6pg,ru5p \right)= \frac{{kI}_{6}(k_{2}k_{4}+k_{2}k_{5}+k_{3}k_{5})}{k_{1}k_{4}+k_{1}k_{5}}$ |
|  | $K_{m}\left( co2,ru5p \right)= \frac{k_{2}k_{4}k_{7}k_{9}+k_{2}k_{5}k_{7}k_{9}+k_{3}k_{5}k_{7}k_{9}}{k_{2}k_{4}k_{8}k_{10}+k_{2}k_{5}k_{8}k_{10}+k_{3}k_{5}k_{8}k_{10}}$ |
|  | $K_{m}\left( co2,nadph \right)= \frac{k_{2}k_{4}k_{7}k_{9}+k_{2}k_{5}k_{7}k_{9}+k_{3}k_{5}k_{7}k_{9}}{k_{2}k_{4}k_{6}k_{10}}$ |
|  | $K_{m}\left( ru5p,nadph \right)= \frac{k_{2}k_{4}k_{7}k_{9}+k_{2}k_{5}k_{7}k_{9}+k_{3}k_{5}k_{7}k_{9}}{k_{2}k_{4}k_{6}k_{8}}$ |
|  | $K_{m}\left( nadph,atp \right)= \frac{{kI}_{1}(k_{2}k_{4}k_{7}+k_{2}k_{5}k_{7}+k_{3}k_{5}k_{7})}{k_{2}k_{4}k_{6}}$ |
|  | $K_{m}\left( nadph,atp \right)= \frac{{kI}_{1}\left( k_{2}k_{4}k_{7}+k_{2}k_{5}k_{7}+k_{3}k_{5}k_{7} \right)}{k_{2}k_{4}k_{6}}$ |
|  | $K_{m}\left( nadph,nadph \right)= \frac{{kI}_{2}(k_{2}k_{4}k_{7}+k_{2}k_{5}k_{7}+k_{3}k_{5}k_{7})}{k_{2}k_{4}k_{6}}$ |
|  | $K_{m}\left( nadph,fdp \right)= \frac{{kI}_{3}(k_{2}k_{4}k_{7}+k_{2}k_{5}k_{7}+k_{3}k_{5}k_{7})}{k_{2}k_{4}k_{6}}$ |
|  | $K_{m}\left( nadph,ru5p \right)= \frac{{kI}_{5}(k_{2}k_{4}k_{7}+k_{2}k_{5}k_{7}+k_{3}k_{5}k_{7})}{k_{2}k_{4}k_{6}}$ |
|  | $K_{m}\left( 6pg,ru5p,nadph \right)= \frac{k_{2}k_{4}k_{7}k_{9}+k_{2}k_{5}k_{7}k_{9}+k_{3}k_{5}k_{7}k_{9}}{k_{1}k_{3}k_{6}k_{8}+k_{1}k_{4}k_{6}k_{8}}$ |
|  | $K_{m}\left( 6pg,nadph,fdp \right)= \frac{{kI}_{4}(k_{2}k_{4}k_{7}+k_{2}k_{5}k_{7}+k_{3}k_{5}k_{7})}{k_{1}k_{4}k_{6}}$ |
|  | $K_{m}\left( 6pg,nadph,ru5p \right)= \frac{{kI}_{6}(k_{2}k_{4}k_{7}+k_{2}k_{5}k_{7}+k_{3}k_{5}k_{7})}{k_{1}k_{4}k_{6}}$ |
|  | $K_{m}\left( co2,ru5p,nadph \right)= \frac{k_{2}k_{4}k_{7}k_{9}+k_{2}k_{5}k_{7}k_{9}+k_{3}k_{5}k_{7}k_{9}}{k_{2}k_{6}k_{8}k_{10}+k_{3}k_{6}k_{8}k_{10}+k_{4}k_{6}k_{8}k_{10}}$ |
|  | $K_{m}\left( ru5p,nadph,ru5p \right)= \frac{{kI}_{5}(k_{2}k_{4}k_{7}k_{9}+k_{2}k_{5}k_{7}k_{9}+k_{3}k_{5}k_{7}k_{9})}{k_{2}k_{4}k_{6}k_{8}}$ |
|  | $K_{m}\left( ru5p,nadph,fdp \right)= \frac{{kI}_{3}(k_{2}k_{4}k_{7}k_{9}+k_{2}k_{5}k_{7}k_{9}+k_{3}k_{5}k_{7}k_{9})}{k_{2}k_{4}k_{6}k_{8}}$ |
|  | $K_{m}\left( ru5p,nadph,nadph \right)= \frac{{kI}_{2}(k_{2}k_{4}k_{7}k_{9}+k_{2}k_{5}k_{7}k_{9}+k_{3}k_{5}k_{7}k_{9})}{k_{2}k_{4}k_{6}k_{8}}$ |
|  | $K_{m}\left( ru5p,nadph,atp \right)= \frac{{kI}_{1}(k_{2}k_{4}k_{7}k_{9}+k_{2}k_{5}k_{7}k_{9}+k_{3}k_{5}k_{7}k_{9})}{k_{2}k_{4}k_{6}k_{8}}$ |
|  | $K_{m}\left( co2,ru5p,nadph,ru5p \right)= \frac{{kI}_{6}(k_{2}k_{4}k_{7}k_{9}+k_{2}k_{5}k_{7}k_{9}+k_{3}k_{5}k_{7}k_{9})}{k_{4}k_{6}k_{8}k_{10}}$ |
|  | $K_{m}\left( co2,ru5p,nadph,fdp \right)= \frac{{kI}_{4}(k_{2}k_{4}k_{7}k_{9}+k_{2}k_{5}k_{7}k_{9}+k_{3}k_{5}k_{7}k_{9})}{k_{4}k_{6}k_{8}k_{10}}$ |
|  | $K_{m}\left( 6pg,ru5p,nadph,ru5p \right)= \frac{{kI}_{6}(k_{2}k_{4}k_{7}k_{9}+k_{2}k_{5}k_{7}k_{9}+k_{3}k_{5}k_{7}k_{9})}{k_{1}k_{4}k_{6}k_{8}}$ |
|  | $K_{m}\left( 6pg,ru5p,nadph,fdp \right)= \frac{{kI}_{4}(k_{2}k_{4}k_{7}k_{9}+k_{2}k_{5}k_{7}k_{9}+k_{3}k_{5}k_{7}k_{9})}{k_{1}k_{4}k_{6}k_{8}}$ |
| RPE | $K_{m}\left( ru5p \right)= \frac{k_{2}k_{4}+k_{2}k_{5}+k_{3}k_{5}}{k_{1}k_{3}+k_{1}k_{4}+k_{1}k_{5}}$ |
|  | $K_{m}\left( x5p \right)= \frac{k_{2}k_{4}+k_{2}k_{5}+k_{3}k_{5}}{k_{2}k_{6}+k_{3}k_{6}+k_{4}k_{6}}$ |
| RPI | $K_{m}\left( ru5p \right)= \frac{k_{2}k_{4}+k_{2}k_{5}+k_{3}k_{5}}{k_{1}k_{3}+k_{1}k_{4}+k_{1}k_{5}}$ |
|  | $K_{m}\left( r5p \right)= \frac{k_{2}k_{4}+k_{2}k_{5}+k_{3}k_{5}}{k_{2}k_{6}+k_{3}k_{6}+k_{4}k_{6}}$ |
|  | $kI\left( 6pg \right)= {kI}_{1}$ |
|  | $kI\left( f6p \right)= {kI}_{2}$ |
| TKThlf1 | $K_{m}\left( x5p \right)= \frac{k_{2}k_{4}k_{7}+k_{2}k_{5}k_{7}+k_{3}k_{5}k_{7}}{k_{1}k_{3}k_{5}+k_{1}k_{3}k_{7}+k_{1}k_{4}k_{7}+k_{1}k_{5}k_{7}}$ |
|  | $K_{m}\left( g3p \right)= \frac{k_{2}k_{4}k_{7}+k_{2}k_{5}k_{7}+k_{3}k_{5}k_{7}}{k_{2}k_{4}k_{8}+k_{2}k_{5}k_{8}+k_{3}k_{5}k_{8}}$ |
|  | $K_{m}\left( ec2 \right)= \frac{k_{2}k_{4}k_{7}+k_{2}k_{5}k_{7}+k_{3}k_{5}k_{7}}{k_{2}k_{4}k_{6}}$ |
|  | $K_{m}\left( x5p,ec2 \right)= \frac{k_{2}k_{4}k_{7}+k_{2}k_{5}k_{7}+k_{3}k_{5}k_{7}}{k_{1}k_{3}k_{6}+k_{1}k_{4}k_{6}}$ |
|  | $K_{m}\left( g3p,ec2 \right)= \frac{k_{2}k_{4}k_{7}+k_{2}k_{5}k_{7}+k_{3}k_{5}k_{7}}{k_{2}k_{6}k_{8}+k_{3}k_{6}k_{8}+k_{4}k_{6}k_{8}}$ |
| TKThlf2 | $K_{m}\left( s7p \right)= \frac{k_{2}k_{4}k_{7}+k_{2}k_{5}k_{7}+k_{3}k_{5}k_{7}}{k_{1}k_{3}k_{5}+k_{1}k_{3}k_{7}+k_{1}k_{4}k_{7}+k_{1}k_{5}k_{7}}$ |
|  | $K_{m}\left( r5p \right)= \frac{k_{2}k_{4}k_{7}+k_{2}k_{5}k_{7}+k_{3}k_{5}k_{7}}{k_{2}k_{4}k_{8}+k_{2}k_{5}k_{8}+k_{3}k_{5}k_{8}}$ |
|  | $K_{m}\left( ec2 \right)= \frac{k_{2}k_{4}k_{7}+k_{2}k_{5}k_{7}+k_{3}k_{5}k_{7}}{k_{2}k_{4}k_{6}}$ |
|  | $K_{m}\left( s7p,ec2 \right)= \frac{k_{2}k_{4}k_{7}+k_{2}k_{5}k_{7}+k_{3}k_{5}k_{7}}{k_{1}k_{3}k_{6}+k_{1}k_{4}k_{6}}$ |
|  | $K_{m}\left( r5p,ec2 \right)= \frac{k_{2}k_{4}k_{7}+k_{2}k_{5}k_{7}+k_{3}k_{5}k_{7}}{k_{2}k_{6}k_{8}+k_{3}k_{6}k_{8}+k_{4}k_{6}k_{8}}$ |
| TKThlf3 | $K_{m}\left( f6p \right)= \frac{k_{2}k_{4}k_{7}+k_{2}k_{5}k_{7}+k_{3}k_{5}k_{7}}{k_{1}k_{3}k_{5}+k_{1}k_{3}k_{7}+k_{1}k_{4}k_{7}+k_{1}k_{5}k_{7}}$ |
|  | $K_{m}\left( e4p \right)= \frac{k_{2}k_{4}k_{7}+k_{2}k_{5}k_{7}+k_{3}k_{5}k_{7}}{k_{2}k_{4}k_{8}+k_{2}k_{5}k_{8}+k_{3}k_{5}k_{8}}$ |
|  | $K_{m}\left( ec2 \right)= \frac{k_{2}k_{4}k_{7}+k_{2}k_{5}k_{7}+k_{3}k_{5}k_{7}}{k_{2}k_{4}k_{6}}$ |
|  | $K_{m}\left( f6p,ec2 \right)= \frac{k_{2}k_{4}k_{7}+k_{2}k_{5}k_{7}+k_{3}k_{5}k_{7}}{k_{1}k_{3}k_{6}+k_{1}k_{4}k_{6}}$ |
|  | $K_{m}\left( e4p,ec2 \right)= \frac{k_{2}k_{4}k_{7}+k_{2}k_{5}k_{7}+k_{3}k_{5}k_{7}}{k_{2}k_{6}k_{8}+k_{3}k_{6}k_{8}+k_{4}k_{6}k_{8}}$ |
| TALA | $K_{m}\left( g3p \right)= \frac{k_{2}k_{4}k_{6}+k_{2}k_{4}k_{7}+k_{2}k_{5}k_{7}}{k_{1}k_{4}k_{6}+k_{1}k_{4}k_{7}+k_{1}k_{5}k_{7}}$ |
|  | $K_{m}\left( s7p \right)= \frac{k_{2}k_{4}k_{6}+k_{2}k_{4}k_{7}+k_{2}k_{5}k_{7}}{k_{3}k_{5}k_{7}}$ |
|  | $K_{m}\left( e4p \right)= \frac{k_{2}k_{4}k_{6}k_{9}+k_{2}k_{4}k_{7}k_{9}+k_{2}k_{5}k_{7}k_{9}}{k_{2}k_{4}k_{6}k_{10}+k_{2}k_{4}k_{7}k_{10}+k_{2}k_{5}k_{7}k_{10}}$ |
|  | $K_{m}\left( f6p \right)= \frac{k_{2}k_{4}k_{6}k_{9}+k_{2}k_{4}k_{7}k_{9}+k_{2}k_{5}k_{7}k_{9}}{k_{2}k_{4}k_{6}k_{8}}$ |
|  | $kI\left( so4 \right)= {kI}_{1}$ |
|  | $K_{m}\left( e4p,f6p \right)= \frac{k_{2}k_{4}k_{6}k_{9}+k_{2}k_{4}k_{7}k_{9}+k_{2}k_{5}k_{7}k_{9}}{k_{2}k_{4}k_{8}k_{10}+k_{2}k_{5}k_{8}k_{10}+k_{2}k_{6}k_{8}k_{10}+k_{4}k_{6}k_{8}k_{10}}$ |
|  | $K_{m}\left( s7p,so4 \right)= \frac{{kI}_{1}(k_{2}k_{4}k_{6}+k_{2}k_{4}k_{7}+k_{2}k_{5}k_{7})}{k_{3}k_{5}k_{7}}$ |
|  | $K_{m}\left( s7p,e4p \right)= \frac{k_{2}k_{4}k_{6}k_{9}+k_{2}k_{4}k_{7}k_{9}+k_{2}k_{5}k_{7}k_{9}}{k_{3}k_{5}k_{7}k_{10}}$ |
|  | $K_{m}\left( g3p,f6p \right)= \frac{k_{2}k_{4}k_{6}k_{9}+k_{2}k_{4}k_{7}k_{9}+k_{2}k_{5}k_{7}k_{9}}{k_{1}k_{4}k_{6}k_{8}}$ |
|  | $K_{m}\left( g3p,s7p \right)= \frac{k_{2}k_{4}k_{6}k_{9}+k_{2}k_{4}k_{7}k_{9}+k_{2}k_{5}k_{7}k_{9}}{k_{1}k_{3}k_{5}k_{7}+k_{1}k_{3}k_{5}k_{9}+k_{1}k_{3}k_{6}k_{9}+k_{1}k_{3}k_{7}k_{9}}$ |
|  | $K_{m}\left( s7p,e4p,f6p \right)= \frac{k_{2}k_{4}k_{6}k_{9}+k_{2}k_{4}k_{7}k_{9}+k_{2}k_{5}k_{7}k_{9}}{k_{3}k_{5}k_{8}k_{10}+k_{3}k_{6}k_{8}k_{10}}$ |
|  | $K_{m}\left( g3p,s7p,f6p \right)= \frac{k_{2}k_{4}k_{6}k_{9}+k_{2}k_{4}k_{7}k_{9}+k_{2}k_{5}k_{7}k_{9}}{k_{1}k_{3}k_{5}k_{8}+k_{1}k_{3}k_{6}k_{8}}$ |
| EDD | $K_{m}\left( 6pg \right)= \frac{k_{2}k_{4}+k_{2}k_{5}+k_{3}k_{5}}{k_{1}k_{3}+k_{1}k_{4}+k_{1}k_{5}}$ |
|  | $K_{m}\left( kdpg \right)= \frac{k_{2}k_{4}+k_{2}k_{5}+k_{3}k_{5}}{k_{2}k_{6}+k_{3}k_{6}+k_{4}k_{6}}$ |
|  | $kI\left( o2 \right)= {kI}_{1}$ |
| EDA | $K_{m}\left( kdpg \right)= \frac{k_{2}k_{4}k_{7}+k_{2}k_{5}k_{7}+k_{3}k_{5}k_{7}}{k_{1}k_{3}k_{5}+k_{1}k_{3}k_{7}+k_{1}k_{4}k_{7}+k_{1}k_{5}k_{7}}$ |
|  | $K_{m}\left( pyr \right)= \frac{k_{2}k_{4}k_{7}+k_{2}k_{5}k_{7}+k_{3}k_{5}k_{7}}{k_{2}k_{4}k_{8}+k_{2}k_{5}k_{8}+k_{3}k_{5}k_{8}}$ |
|  | $K_{m}\left( g3p \right)= \frac{k_{2}k_{4}k_{7}+k_{2}k_{5}k_{7}+k_{3}k_{5}k_{7}}{k_{2}k_{4}k_{6}}$ |
|  | $kI\left( 6pg \right)= {kI}_{1}$ |
|  | $kI\left( g3p \right)= {kI}_{2}$ |
|  | $K_{m}\left( g3p,g3p \right)= \frac{{kI}_{2}\left( k_{2}k_{4}k_{7}+k_{2}k_{5}k_{7}+k_{3}k_{5}k_{7} \right)}{k_{2}k_{4}k_{6}}$ |
|  | $K_{m}\left( g3p,6pg \right)= \frac{{kI}_{1}(k_{2}k_{4}k_{7}+k_{2}k_{5}k_{7}+k_{3}k_{5}k_{7})}{k_{2}k_{4}k_{6}}$ |
|  | $K_{m}\left( pyr,g3p \right)= \frac{k_{2}k_{4}k_{7}+k_{2}k_{5}k_{7}+k_{3}k_{5}k_{7}}{k_{2}k_{6}k_{8}+k_{3}k_{6}k_{8}+k_{4}k_{6}k_{8}}$ |
|  | $K_{m}\left( kdpg,g3p \right)= \frac{{kI}_{3}(k_{2}k_{4}+k_{2}k_{5}+k_{3}k_{5})}{k_{1}k_{4}+k_{1}k_{5}}$ |
|  | $K_{m}\left( kdpg,g3p \right)= \frac{k_{2}k_{4}k_{7}+k_{2}k_{5}k_{7}+k_{3}k_{5}k_{7}}{k_{1}k_{3}k_{6}+k_{1}k_{4}k_{6}}$ |
|  | $K_{m}\left( pyr,g3p,g3p \right)= \frac{{kI}_{3}(k_{2}k_{4}k_{7}+k_{2}k_{5}k_{7}+k_{3}k_{5}k_{7})}{k_{4}k_{6}k_{8}}$ |
|  | $K_{m}\left( kdpg,g3p,g3p \right)= \frac{{kI}_{3}(k_{2}k_{4}k_{7}+k_{2}k_{5}k_{7}+k_{3}k_{5}k_{7})}{k_{1}k_{4}k_{6}}$ |
| PDH | $K_{m}\left( pyr \right)= \frac{k_{2}k_{4}k_{7}k_{9}+k_{2}k_{5}k_{7}k_{9}+k_{3}k_{5}k_{7}k_{9}}{k_{1}k_{3}k_{5}k_{7}+k_{1}k_{3}k_{5}k_{9}+k_{1}k_{3}k_{7}k_{9}+k_{1}k_{4}k_{7}k_{9}+k_{1}k_{5}k_{7}k_{9}}$ |
|  | $K_{m}\left( accoa \right)= \frac{k_{2}k_{4}k_{9}+k_{2}k_{5}k_{9}+k_{3}k_{5}k_{9}}{k_{2}k_{4}k_{10}+k_{2}k_{5}k_{10}+k_{3}k_{5}k_{10}}$ |
|  | $K_{m}\left( nadh \right)= \frac{k_{2}k_{4}k_{7}+k_{2}k_{5}k_{7}+k_{3}k_{5}k_{7}}{k_{2}k_{4}k_{6}}$ |
|  | $K_{m}\left( co2,nadh \right)= \frac{k_{2}k_{4}k_{7}k_{9}+k_{2}k_{5}k_{7}k_{9}+k_{3}k_{5}k_{7}k_{9}}{k_{2}k_{4}k_{6}k_{8}}$ |
|  | $K_{m}\left( accoa,nadh \right)= \frac{k_{2}k_{4}k_{7}k_{9}+k_{2}k_{5}k_{7}k_{9}+k_{3}k_{5}k_{7}k_{9}}{k_{2}k_{4}k_{6}k_{10}}$ |
|  | $K_{m}\left( accoa,co2 \right)= \frac{k_{2}k_{4}k_{7}k_{9}+k_{2}k_{5}k_{7}k_{9}+k_{3}k_{5}k_{7}k_{9}}{k_{2}k_{4}k_{8}k_{10}+k_{2}k_{5}k_{8}k_{10}+k_{3}k_{5}k_{8}k_{10}}$ |
|  | $K_{m}\left( pyr,nadh \right)= \frac{k_{2}k_{4}k_{7}+k_{2}k_{5}k_{7}+k_{3}k_{5}k_{7}}{k_{1}k_{3}k_{6}+k_{1}k_{4}k_{6}}$ |
|  | $K_{m}\left( pyr,co2 \right)= \frac{k_{2}k_{4}k_{7}k_{9}+k_{2}k_{5}k_{7}k_{9}+k_{3}k_{5}k_{7}k_{9}}{k_{1}k_{3}k_{5}k_{8}}$ |
|  | $K_{m}\left( pyr,co2,nadh \right)= \frac{k_{2}k_{4}k_{7}k_{9}+k_{2}k_{5}k_{7}k_{9}+k_{3}k_{5}k_{7}k_{9}}{k_{1}k_{3}k_{6}k_{8}+k_{1}k_{4}k_{6}k_{8}}$ |
|  | $K_{m}\left( accoa,co2,nadh \right)= \frac{k_{2}k_{4}k_{7}k_{9}+k_{2}k_{5}k_{7}k_{9}+k_{3}k_{5}k_{7}k_{9}}{k_{2}k_{6}k_{8}k_{10}+k_{3}k_{6}k_{8}k_{10}+k_{4}k_{6}k_{8}k_{10}}$ |
| CS | $K_{m}\left( accoa \right)= \frac{k_{2}k_{4}k_{6}+k_{2}k_{4}k_{7}+k_{2}k_{5}k_{7}}{k_{1}k_{4}k_{6}+k_{1}k_{4}k_{7}+k_{1}k_{5}k_{7}}$ |
|  | $K_{m}\left( oac \right)= \frac{k_{2}k_{4}k_{6}+k_{2}k_{4}k_{7}+k_{2}k_{5}k_{7}}{k_{3}k_{5}k_{7}}$ |
|  | $K_{m}\left( cit \right)= \frac{k_{2}k_{4}k_{6}+k_{2}k_{4}k_{7}+k_{2}k_{5}k_{7}}{k_{2}k_{4}k_{8}+k_{2}k_{5}k_{8}+k_{2}k_{6}k_{8}+k_{4}k_{6}k_{8}}$ |
|  | $K_{m}\left( accoa,oac \right)= \frac{k_{2}k_{4}k_{6}+k_{2}k_{4}k_{7}+k_{2}k_{5}k_{7}}{k_{1}k_{3}k_{5}+k_{1}k_{3}k_{6}+k_{1}k_{3}k_{7}}$ |
|  | $K_{m}\left( oac,cit \right)= \frac{k_{2}k_{4}k_{6}+k_{2}k_{4}k_{7}+k_{2}k_{5}k_{7}}{k_{3}k_{5}k_{8}+k_{3}k_{6}k_{8}}$ |
| ACONT | $K_{m}\left( cit \right)= \frac{k_{2}k_{4}+k_{2}k_{5}+k_{3}k_{5}}{k_{1}k_{3}+k_{1}k_{4}+k_{1}k_{5}}$ |
|  | $K_{m}\left( icit \right)= \frac{k_{2}k_{4}+k_{2}k_{5}+k_{3}k_{5}}{k_{2}k_{6}+k_{3}k_{6}+k_{4}k_{6}}$ |
| ICDHyr | $K_{m}\left( icit \right)= \frac{k_{2}k_{4}k_{7}k_{9}+k_{2}k_{5}k_{7}k_{9}+k_{3}k_{5}k_{7}k_{9}}{k_{1}k_{3}k_{5}k_{7}+k_{1}k_{3}k_{5}k_{9}+k_{1}k_{3}k_{7}k_{9}+k_{1}k_{4}k_{7}k_{9}+k_{1}k_{5}k_{7}k_{9}}$ |
|  | $K_{m}\left( akg \right)= \frac{k_{2}k_{4}k_{9}+k_{2}k_{5}k_{9}+k_{3}k_{5}k_{9}}{k_{2}k_{4}k_{10}+k_{2}k_{5}k_{10}+k_{3}k_{5}k_{10}}$ |
|  | $K_{m}\left( nadph \right)= \frac{k_{2}k_{4}k_{7}+k_{2}k_{5}k_{7}+k_{3}k_{5}k_{7}}{k_{2}k_{4}k_{6}}$ |
|  | $kI\left( glx \right)= {kI}_{1}$ |
|  | $kI\left( oac \right)= {kI}_{2}$ |
|  | $kI\left( pep \right)= {kI}_{3}$ |
|  | $K_{m}\left( icit,co2 \right)= \frac{k_{2}k_{4}k_{7}k_{9}+k_{2}k_{5}k_{7}k_{9}+k_{3}k_{5}k_{7}k_{9}}{k_{1}k_{3}k_{5}k_{8}}$ |
|  | $K_{m}\left( icit,nadph \right)= \frac{k_{2}k_{4}k_{7}+k_{2}k_{5}k_{7}+k_{3}k_{5}k_{7}}{k_{1}k_{3}k_{6}+k_{1}k_{4}k_{6}}$ |
|  | $K_{m}\left( icit,pep \right)= \frac{{kI}_{4}(k_{2}k_{4}+k_{2}k_{5}+k_{3}k_{5})}{k_{1}k_{4}+k_{1}k_{5}}$ |
|  | $K_{m}\left( akg,co2 \right)= \frac{k_{2}k_{4}k_{7}k_{9}+k_{2}k_{5}k_{7}k_{9}+k_{3}k_{5}k_{7}k_{9}}{k_{2}k_{4}k_{8}k_{10}+k_{2}k_{5}k_{8}k_{10}+k_{3}k_{5}k_{8}k_{10}}$ |
|  | $K_{m}\left( akg,nadph \right)= \frac{k_{2}k_{4}k_{7}k_{9}+k_{2}k_{5}k_{7}k_{9}+k_{3}k_{5}k_{7}k_{9}}{k_{2}k_{4}k_{6}k_{10}}$ |
|  | $K_{m}\left( co2,nadph \right)= \frac{k_{2}k_{4}k_{7}k_{9}+k_{2}k_{5}k_{7}k_{9}+k_{3}k_{5}k_{7}k_{9}}{k_{2}k_{4}k_{6}k_{8}}$ |
|  | $K_{m}\left( nadph,glx \right)= \frac{{kI}_{1}\left( k_{2}k_{4}k_{7}+k_{2}k_{5}k_{7}+k_{3}k_{5}k_{7} \right)}{k_{2}k_{4}k_{6}}$ |
|  | $K_{m}\left( nadph,oac \right)= \frac{{kI}_{2}(k_{2}k_{4}k_{7}+k_{2}k_{5}k_{7}+k_{3}k_{5}k_{7})}{k_{2}k_{4}k_{6}}$ |
|  | $K_{m}\left( nadph,pep \right)= \frac{{kI}_{3}(k_{2}k_{4}k_{7}+k_{2}k_{5}k_{7}+k_{3}k_{5}k_{7})}{k_{2}k_{4}k_{6}}$ |
|  | $K_{m}\left( icit,co2,nadph \right)= \frac{k_{2}k_{4}k_{7}k_{9}+k_{2}k_{5}k_{7}k_{9}+k_{3}k_{5}k_{7}k_{9}}{k_{1}k_{3}k_{6}k_{8}+k_{1}k_{4}k_{6}k_{8}}$ |
|  | $K_{m}\left( icit,nadph,pep \right)= \frac{{kI}_{4}(k_{2}k_{4}k_{7}+k_{2}k_{5}k_{7}+k_{3}k_{5}k_{7})}{k_{1}k_{4}k_{6}}$ |
|  | $K_{m}\left( akg,co2,nadph \right)= \frac{k_{2}k_{4}k_{7}k_{9}+k_{2}k_{5}k_{7}k_{9}+k_{3}k_{5}k_{7}k_{9}}{k_{2}k_{6}k_{8}k_{10}+k_{3}k_{6}k_{8}k_{10}+k_{4}k_{6}k_{8}k_{10}}$ |
|  | $K_{m}\left( co2,nadph,pep \right)= \frac{{kI}_{3}(k_{2}k_{4}k_{7}k_{9}+k_{2}k_{5}k_{7}k_{9}+k_{3}k_{5}k_{7}k_{9})}{k_{2}k_{4}k_{6}k_{8}}$ |
|  | $K_{m}\left( co2,nadph,oac \right)= \frac{{kI}_{2}(k_{2}k_{4}k_{7}k_{9}+k_{2}k_{5}k_{7}k_{9}+k_{3}k_{5}k_{7}k_{9})}{k_{2}k_{4}k_{6}k_{8}}$ |
|  | $K_{m}\left( co2,nadph,glx \right)= \frac{{kI}_{1}(k_{2}k_{4}k_{7}k_{9}+k_{2}k_{5}k_{7}k_{9}+k_{3}k_{5}k_{7}k_{9})}{k_{2}k_{4}k_{6}k_{8}}$ |
|  | $K_{m}\left( akg,co2,nadph,pep \right)= \frac{{kI}_{4}(k_{2}k_{4}k_{7}k_{9}+k_{2}k_{5}k_{7}k_{9}+k_{3}k_{5}k_{7}k_{9})}{k_{4}k_{6}k_{8}k_{10}}$ |
|  | $K_{m}\left( icit,co2,nadph,pep \right)= \frac{{kI}_{4}(k_{2}k_{4}k_{7}k_{9}+k_{2}k_{5}k_{7}k_{9}+k_{3}k_{5}k_{7}k_{9})}{k_{1}k_{4}k_{6}k_{8}}$ |
| AKGDH | $K_{m}\left( akg \right)= \frac{k_{2}k_{4}k_{7}k_{9}+k_{2}k_{5}k_{7}k_{9}+k_{3}k_{5}k_{7}k_{9}}{k_{1}k_{3}k_{5}k_{7}+k_{1}k_{3}k_{5}k_{9}+k_{1}k_{3}k_{7}k_{9}+k_{1}k_{4}k_{7}k_{9}+k_{1}k_{5}k_{7}k_{9}}$ |
|  | $K_{m}\left( succoa \right)= \frac{k_{2}k_{4}k_{9}+k_{2}k_{5}k_{9}+k_{3}k_{5}k_{9}}{k_{2}k_{4}k_{10}+k_{2}k_{5}k_{10}+k_{3}k_{5}k_{10}}$ |
|  | $K_{m}\left( nadh \right)= \frac{k_{2}k_{4}k_{7}+k_{2}k_{5}k_{7}+k_{3}k_{5}k_{7}}{k_{2}k_{4}k_{6}}$ |
|  | $K_{m}\left( co2,nadh \right)= \frac{k_{2}k_{4}k_{7}k_{9}+k_{2}k_{5}k_{7}k_{9}+k_{3}k_{5}k_{7}k_{9}}{k_{2}k_{4}k_{6}k_{8}}$ |
|  | $K_{m}\left( succoa,nadh \right)= \frac{k_{2}k_{4}k_{7}k_{9}+k_{2}k_{5}k_{7}k_{9}+k_{3}k_{5}k_{7}k_{9}}{k_{2}k_{4}k_{6}k_{10}}$ |
|  | $K_{m}\left( succoa,co2 \right)= \frac{k_{2}k_{4}k_{7}k_{9}+k_{2}k_{5}k_{7}k_{9}+k_{3}k_{5}k_{7}k_{9}}{k_{2}k_{4}k_{8}k_{10}+k_{2}k_{5}k_{8}k_{10}+k_{3}k_{5}k_{8}k_{10}}$ |
|  | $K_{m}\left( akg,nadh \right)= \frac{k_{2}k_{4}k_{7}+k_{2}k_{5}k_{7}+k_{3}k_{5}k_{7}}{k_{1}k_{3}k_{6}+k_{1}k_{4}k_{6}}$ |
|  | $K_{m}\left( akg,co2 \right)= \frac{k_{2}k_{4}k_{7}k_{9}+k_{2}k_{5}k_{7}k_{9}+k_{3}k_{5}k_{7}k_{9}}{k_{1}k_{3}k_{5}k_{8}}$ |
|  | $K_{m}\left( akg,co2,nadh \right)= \frac{k_{2}k_{4}k_{7}k_{9}+k_{2}k_{5}k_{7}k_{9}+k_{3}k_{5}k_{7}k_{9}}{k_{1}k_{3}k_{6}k_{8}+k_{1}k_{4}k_{6}k_{8}}$ |
|  | $K_{m}\left( succoa,co2,nadh \right)= \frac{k_{2}k_{4}k_{7}k_{9}+k_{2}k_{5}k_{7}k_{9}+k_{3}k_{5}k_{7}k_{9}}{k_{2}k_{6}k_{8}k_{10}+k_{3}k_{6}k_{8}k_{10}+k_{4}k_{6}k_{8}k_{10}}$ |
| SUCOAS | $K_{m}\left( succoa \right)= \frac{k_{2}k_{4}k_{7}+k_{2}k_{5}k_{7}+k_{3}k_{5}k_{7}}{k_{1}k_{3}k_{5}+k_{1}k_{3}k_{7}+k_{1}k_{4}k_{7}+k_{1}k_{5}k_{7}}$ |
|  | $K_{m}\left( suc \right)= \frac{k_{2}k_{4}k_{7}+k_{2}k_{5}k_{7}+k_{3}k_{5}k_{7}}{k_{2}k_{4}k_{8}+k_{2}k_{5}k_{8}+k_{3}k_{5}k_{8}}$ |
|  | $K_{m}\left( atp \right)= \frac{k_{2}k_{4}k_{7}+k_{2}k_{5}k_{7}+k_{3}k_{5}k_{7}}{k_{2}k_{4}k_{6}}$ |
|  | $kI\left( atp \right)= {kI}_{1}$ |
|  | $kI\left( nadh \right)= {kI}_{2}$ |
|  | $kI\left( akg \right)= {kI}_{3}$ |
|  | $kI\left( suc \right)= {kI}_{4}$ |
|  | $K_{m}\left( succoa,atp \right)= \frac{k_{2}k_{4}k_{7}+k_{2}k_{5}k_{7}+k_{3}k_{5}k_{7}}{k_{1}k_{3}k_{6}+k_{1}k_{4}k_{6}}$ |
|  | $K_{m}\left( succoa,suc \right)= \frac{{kI}_{5}(k_{2}k_{4}+k_{2}k_{5}+k_{3}k_{5})}{k_{1}k_{4}+k_{1}k_{5}}$ |
|  | $K_{m}\left( suc,atp \right)= \frac{k_{2}k_{4}k_{7}+k_{2}k_{5}k_{7}+k_{3}k_{5}k_{7}}{k_{2}k_{6}k_{8}+k_{3}k_{6}k_{8}+k_{4}k_{6}k_{8}}$ |
|  | $K_{m}\left( atp,atp \right)= \frac{{kI}_{1}(k_{2}k_{4}k_{7}+k_{2}k_{5}k_{7}+k_{3}k_{5}k_{7})}{k_{2}k_{4}k_{6}}$ |
|  | $K_{m}\left( atp,nadh \right)= \frac{{kI}_{2}(k_{2}k_{4}k_{7}+k_{2}k_{5}k_{7}+k_{3}k_{5}k_{7})}{k_{2}k_{4}k_{6}}$ |
|  | $K_{m}\left( atp,akg \right)= \frac{{kI}_{3}(k_{2}k_{4}k_{7}+k_{2}k_{5}k_{7}+k_{3}k_{5}k_{7})}{k_{2}k_{4}k_{6}}$ |
|  | $K_{m}\left( atp,suc \right)= \frac{{kI}_{4}(k_{2}k_{4}k_{7}+k_{2}k_{5}k_{7}+k_{3}k_{5}k_{7})}{k_{2}k_{4}k_{6}}$ |
|  | $K_{m}\left( succoa,atp,suc \right)= \frac{{kI}_{5}(k_{2}k_{4}k_{7}+k_{2}k_{5}k_{7}+k_{3}k_{5}k_{7})}{k_{1}k_{4}k_{6}}$ |
|  | $K_{m}\left( suc,atp,suc \right)= \frac{{kI}_{5}(k_{2}k_{4}k_{7}+k_{2}k_{5}k_{7}+k_{3}k_{5}k_{7})}{k_{4}k_{6}k_{8}}$ |
| SUCDi | $K_{m}\left( suc \right)= \frac{k_{2}k_{4}k_{7}+k_{2}k_{5}k_{7}+k_{3}k_{5}k_{7}}{k_{1}k_{3}k_{5}+k_{1}k_{3}k_{7}+k_{1}k_{4}k_{7}+k_{1}k_{5}k_{7}}$ |
|  | $K_{m}\left( fadh2 \right)= \frac{k_{2}k_{4}k_{7}+k_{2}k_{5}k_{7}+k_{3}k_{5}k_{7}}{k_{2}k_{4}k_{8}+k_{2}k_{5}k_{8}+k_{3}k_{5}k_{8}}$ |
|  | $K_{m}\left( fum \right)= \frac{k_{2}k_{4}k_{7}+k_{2}k_{5}k_{7}+k_{3}k_{5}k_{7}}{k_{2}k_{4}k_{6}}$ |
|  | $K_{m}\left( suc,fum \right)= \frac{k_{2}k_{4}k_{7}+k_{2}k_{5}k_{7}+k_{3}k_{5}k_{7}}{k_{1}k_{3}k_{6}+k_{1}k_{4}k_{6}}$ |
|  | $K_{m}\left( fadh2,fum \right)= \frac{k_{2}k_{4}k_{7}+k_{2}k_{5}k_{7}+k_{3}k_{5}k_{7}}{k_{2}k_{6}k_{8}+k_{3}k_{6}k_{8}+k_{4}k_{6}k_{8}}$ |
| FUM | $K_{m}\left( fum \right)= \frac{k_{2}k_{4}+k_{2}k_{5}+k_{3}k_{5}}{k_{1}k_{3}+k_{1}k_{4}+k_{1}k_{5}}$ |
|  | $K_{m}\left( mal \right)= \frac{k_{2}k_{4}+k_{2}k_{5}+k_{3}k_{5}}{k_{2}k_{6}+k_{3}k_{6}+k_{4}k_{6}}$ |
|  | $kI\left( cit \right)= {kI}_{1}$ |
| MDH | $K_{m}\left( mal \right)= \frac{k_{2}k_{4}k_{7}+k_{2}k_{5}k_{7}+k_{3}k_{5}k_{7}}{k_{1}k_{3}k_{5}+k_{1}k_{3}k_{7}+k_{1}k_{4}k_{7}+k_{1}k_{5}k_{7}}$ |
|  | $K_{m}\left( oac \right)= \frac{k_{2}k_{4}k_{7}+k_{2}k_{5}k_{7}+k_{3}k_{5}k_{7}}{k_{2}k_{4}k_{8}+k_{2}k_{5}k_{8}+k_{3}k_{5}k_{8}}$ |
|  | $K_{m}\left( nadh \right)= \frac{k_{2}k_{4}k_{7}+k_{2}k_{5}k_{7}+k_{3}k_{5}k_{7}}{k_{2}k_{4}k_{6}}$ |
|  | $K_{m}\left( mal,nadh \right)= \frac{k_{2}k_{4}k_{7}+k_{2}k_{5}k_{7}+k_{3}k_{5}k_{7}}{k_{1}k_{3}k_{6}+k_{1}k_{4}k_{6}}$ |
|  | $K_{m}\left( oac,nadh \right)= \frac{k_{2}k_{4}k_{7}+k_{2}k_{5}k_{7}+k_{3}k_{5}k_{7}}{k_{2}k_{6}k_{8}+k_{3}k_{6}k_{8}+k_{4}k_{6}k_{8}}$ |
| ICL | $K_{m}\left( icit \right)= \frac{k_{2}k_{4}k_{7}+k_{2}k_{5}k_{7}+k_{3}k_{5}k_{7}}{k_{1}k_{3}k_{5}+k_{1}k_{3}k_{7}+k_{1}k_{4}k_{7}+k_{1}k_{5}k_{7}}$ |
|  | $K_{m}\left( suc \right)= \frac{k_{2}k_{4}k_{7}+k_{2}k_{5}k_{7}+k_{3}k_{5}k_{7}}{k_{2}k_{4}k_{8}+k_{2}k_{5}k_{8}+k_{3}k_{5}k_{8}}$ |
|  | $K_{m}\left( glx \right)= \frac{k_{2}k_{4}k_{7}+k_{2}k_{5}k_{7}+k_{3}k_{5}k_{7}}{k_{2}k_{4}k_{6}}$ |
|  | $kI\left( glx \right)= {kI}_{1}$ |
|  | $kI\left( 3pg \right)= {kI}_{2}$ |
|  | $kI\left( so4 \right)= {kI}_{3}$ |
|  | $kI\left( akg \right)= {kI}_{5}$ |
|  | $kI\left( suc \right)= {kI}_{7}$ |
|  | $K_{m}\left( icit,glx \right)= \frac{k_{2}k_{4}k_{7}+k_{2}k_{5}k_{7}+k_{3}k_{5}k_{7}}{k_{1}k_{3}k_{6}+k_{1}k_{4}k_{6}}$ |
|  | $K_{m}\left( icit,pep \right)= \frac{{kI}_{4}(k_{2}k_{4}+k_{2}k_{5}+k_{3}k_{5})}{k_{1}k_{4}+k_{1}k_{5}}$ |
|  | $K_{m}\left( icit,akg \right)= \frac{{kI}_{6}(k_{2}k_{4}+k_{2}k_{5}+k_{3}k_{5})}{k_{1}k_{4}+k_{1}k_{5}}$ |
|  | $K_{m}\left( icit,suc \right)= \frac{{kI}_{8}(k_{2}k_{4}+k_{2}k_{5}+k_{3}k_{5})}{k_{1}k_{4}+k_{1}k_{5}}$ |
|  | $K_{m}\left( suc,glx \right)= \frac{k_{2}k_{4}k_{7}+k_{2}k_{5}k_{7}+k_{3}k_{5}k_{7}}{k_{2}k_{6}k_{8}+k_{3}k_{6}k_{8}+k_{4}k_{6}k_{8}}$ |
|  | $K_{m}\left( glx,glx \right)= \frac{{kI}_{1}(k_{2}k_{4}k_{7}+k_{2}k_{5}k_{7}+k_{3}k_{5}k_{7})}{k_{2}k_{4}k_{6}}$ |
|  | $K_{m}\left( glx,3pg \right)= \frac{{kI}_{2}(k_{2}k_{4}k_{7}+k_{2}k_{5}k_{7}+k_{3}k_{5}k_{7})}{k_{2}k_{4}k_{6}}$ |
|  | $K_{m}\left( glx,so4 \right)= \frac{{kI}_{3}(k_{2}k_{4}k_{7}+k_{2}k_{5}k_{7}+k_{3}k_{5}k_{7})}{k_{2}k_{4}k_{6}}$ |
|  | $K_{m}\left( glx,akg \right)= \frac{{kI}_{5}(k_{2}k_{4}k_{7}+k_{2}k_{5}k_{7}+k_{3}k_{5}k_{7})}{k_{2}k_{4}k_{6}}$ |
|  | $K_{m}\left( glx,suc \right)= \frac{{kI}_{7}(k_{2}k_{4}k_{7}+k_{2}k_{5}k_{7}+k_{3}k_{5}k_{7})}{k_{2}k_{4}k_{6}}$ |
|  | $K_{m}\left( icit,glx,pep \right)= \frac{{kI}_{4}(k_{2}k_{4}k_{7}+k_{2}k_{5}k_{7}+k_{3}k_{5}k_{7})}{k_{1}k_{4}k_{6}}$ |
|  | $K_{m}\left( icit,glx,akg \right)= \frac{{kI}_{6}(k_{2}k_{4}k_{7}+k_{2}k_{5}k_{7}+k_{3}k_{5}k_{7})}{k_{1}k_{4}k_{6}}$ |
|  | $K_{m}\left( icit,glx,suc \right)= \frac{{kI}_{8}(k_{2}k_{4}k_{7}+k_{2}k_{5}k_{7}+k_{3}k_{5}k_{7})}{k_{1}k_{4}k_{6}}$ |
|  | $K_{m}\left( suc,glx,pep \right)= \frac{{kI}_{4}(k_{2}k_{4}k_{7}+k_{2}k_{5}k_{7}+k_{3}k_{5}k_{7})}{k_{4}k_{6}k_{8}}$ |
|  | $K_{m}\left( suc,glx,akg \right)= \frac{{kI}_{6}(k_{2}k_{4}k_{7}+k_{2}k_{5}k_{7}+k_{3}k_{5}k_{7})}{k_{4}k_{6}k_{8}}$ |
|  | $K_{m}\left( suc,glx,suc \right)= \frac{{kI}_{8}(k_{2}k_{4}k_{7}+k_{2}k_{5}k_{7}+k_{3}k_{5}k_{7})}{k_{4}k_{6}k_{8}}$ |
| MALS | $K_{m}\left( glx \right)= \frac{k_{2}k_{4}k_{6}+k_{2}k_{4}k_{7}+k_{2}k_{5}k_{7}}{k_{1}k_{4}k_{6}+k_{1}k_{4}k_{7}+k_{1}k_{5}k_{7}}$ |
|  | $K_{m}\left( accoa \right)= \frac{k_{2}k_{4}k_{6}+k_{2}k_{4}k_{7}+k_{2}k_{5}k_{7}}{k_{3}k_{5}k_{7}}$ |
|  | $K_{m}\left( mal \right)= \frac{k_{2}k_{4}k_{6}+k_{2}k_{4}k_{7}+k_{2}k_{5}k_{7}}{k_{2}k_{4}k_{8}+k_{2}k_{5}k_{8}+k_{2}k_{6}k_{8}+k_{4}k_{6}k_{8}}$ |
|  | $K_{m}\left( glx,accoa \right)= \frac{k_{2}k_{4}k_{6}+k_{2}k_{4}k_{7}+k_{2}k_{5}k_{7}}{k_{1}k_{3}k_{5}+k_{1}k_{3}k_{6}+k_{1}k_{3}k_{7}}$ |
|  | $K_{m}\left( accoa,mal \right)= \frac{k_{2}k_{4}k_{6}+k_{2}k_{4}k_{7}+k_{2}k_{5}k_{7}}{k_{3}k_{5}k_{8}+k_{3}k_{6}k_{8}}$ |
| ME2 | $K_{m}\left( mal \right)= \frac{k_{2}k_{4}k_{7}k_{9}+k_{2}k_{5}k_{7}k_{9}+k_{3}k_{5}k_{7}k_{9}}{k_{1}k_{3}k_{5}k_{7}+k_{1}k_{3}k_{5}k_{9}+k_{1}k_{3}k_{7}k_{9}+k_{1}k_{4}k_{7}k_{9}+k_{1}k_{5}k_{7}k_{9}}$ |
|  | $K_{m}\left( co2 \right)= \frac{k_{2}k_{4}k_{9}+k_{2}k_{5}k_{9}+k_{3}k_{5}k_{9}}{k_{2}k_{4}k_{10}+k_{2}k_{5}k_{10}+k_{3}k_{5}k_{10}}$ |
|  | $K_{m}\left( pyr \right)= \frac{k_{2}k_{4}k_{7}+k_{2}k_{5}k_{7}+k_{3}k_{5}k_{7}}{k_{2}k_{4}k_{6}}$ |
|  | $kI\left( atp \right)= {kI}_{1}$ |
|  | $kI\left( accoa \right)= {kI}_{3}$ |
|  | $K_{m}\left( mal,nadph \right)= \frac{k_{2}k_{4}k_{7}k_{9}+k_{2}k_{5}k_{7}k_{9}+k_{3}k_{5}k_{7}k_{9}}{k_{1}k_{3}k_{5}k_{8}}$ |
|  | $K_{m}\left( mal,pyr \right)= \frac{k_{2}k_{4}k_{7}+k_{2}k_{5}k_{7}+k_{3}k_{5}k_{7}}{k_{1}k_{3}k_{6}+k_{1}k_{4}k_{6}}$ |
|  | $K_{m}\left( mal,atp \right)= \frac{{kI}_{2}(k_{2}k_{4}+k_{2}k_{5}+k_{3}k_{5})}{k_{1}k_{4}+k_{1}k_{5}}$ |
|  | $K_{m}\left( mal,accoa \right)= \frac{{kI}_{4}(k_{2}k_{4}+k_{2}k_{5}+k_{3}k_{5})}{k_{1}k_{4}+k_{1}k_{5}}$ |
|  | $K_{m}\left( co2,nadph \right)= \frac{k_{2}k_{4}k_{7}k_{9}+k_{2}k_{5}k_{7}k_{9}+k_{3}k_{5}k_{7}k_{9}}{k_{2}k_{4}k_{8}k_{10}+k_{2}k_{5}k_{8}k_{10}+k_{3}k_{5}k_{8}k_{10}}$ |
|  | $K_{m}\left( co2,pyr \right)= \frac{k_{2}k_{4}k_{7}k_{9}+k_{2}k_{5}k_{7}k_{9}+k_{3}k_{5}k_{7}k_{9}}{k_{2}k_{4}k_{6}k_{10}}$ |
|  | $K_{m}\left( nadph,pyr \right)= \frac{k_{2}k_{4}k_{7}k_{9}+k_{2}k_{5}k_{7}k_{9}+k_{3}k_{5}k_{7}k_{9}}{k_{2}k_{4}k_{6}k_{8}}$ |
|  | $K_{m}\left( pyr,atp \right)= \frac{{kI}_{1}(k_{2}k_{4}k_{7}+k_{2}k_{5}k_{7}+k_{3}k_{5}k_{7})}{k_{2}k_{4}k_{6}}$ |
|  | $K_{m}\left( pyr,accoa \right)= \frac{{kI}_{3}(k_{2}k_{4}k_{7}+k_{2}k_{5}k_{7}+k_{3}k_{5}k_{7})}{k_{2}k_{4}k_{6}}$ |
|  | $K_{m}\left( nadph,pyr,accoa \right)= \frac{{kI}_{3}(k_{2}k_{4}k_{7}k_{9}+k_{2}k_{5}k_{7}k_{9}+k_{3}k_{5}k_{7}k_{9})}{k_{2}k_{4}k_{6}k_{8}}$ |
|  | $K_{m}\left( nadph,pyr,atp \right)= \frac{{kI}_{1}(k_{2}k_{4}k_{7}k_{9}+k_{2}k_{5}k_{7}k_{9}+k_{3}k_{5}k_{7}k_{9})}{k_{2}k_{4}k_{6}k_{8}}$ |
|  | $K_{m}\left( co2,nadph,pyr \right)= \frac{k_{2}k_{4}k_{7}k_{9}+k_{2}k_{5}k_{7}k_{9}+k_{3}k_{5}k_{7}k_{9}}{k_{2}k_{6}k_{8}k_{10}+k_{3}k_{6}k_{8}k_{10}+k_{4}k_{6}k_{8}k_{10}}$ |
|  | $K_{m}\left( mal,pyr,accoa \right)= \frac{{kI}_{4}(k_{2}k_{4}k_{7}+k_{2}k_{5}k_{7}+k_{3}k_{5}k_{7})}{k_{1}k_{4}k_{6}}$ |
|  | $K_{m}\left( mal,pyr,atp \right)= \frac{{kI}_{2}(k_{2}k_{4}k_{7}+k_{2}k_{5}k_{7}+k_{3}k_{5}k_{7})}{k_{1}k_{4}k_{6}}$ |
|  | $K_{m}\left( co2,nadph,pyr,accoa \right)= \frac{{kI}_{4}(k_{2}k_{4}k_{7}k_{9}+k_{2}k_{5}k_{7}k_{9}+k_{3}k_{5}k_{7}k_{9})}{k_{4}k_{6}k_{8}k_{10}}$ |
|  | $K_{m}\left( co2,nadph,pyr,atp \right)= \frac{{kI}_{2}(k_{2}k_{4}k_{7}k_{9}+k_{2}k_{5}k_{7}k_{9}+k_{3}k_{5}k_{7}k_{9})}{k_{4}k_{6}k_{8}k_{10}}$ |
|  | $K_{m}\left( mal,nadph,pyr,accoa \right)= \frac{{kI}_{4}(k_{2}k_{4}k_{7}k_{9}+k_{2}k_{5}k_{7}k_{9}+k_{3}k_{5}k_{7}k_{9})}{k_{1}k_{4}k_{6}k_{8}}$ |
|  | $K_{m}\left( mal,nadph,pyr,atp \right)= \frac{{kI}_{2}(k_{2}k_{4}k_{7}k_{9}+k_{2}k_{5}k_{7}k_{9}+k_{3}k_{5}k_{7}k_{9})}{k_{1}k_{4}k_{6}k_{8}}$ |
| ME1 | $K_{m}\left( mal \right)= \frac{k_{2}k_{4}k_{7}k_{9}+k_{2}k_{5}k_{7}k_{9}+k_{3}k_{5}k_{7}k_{9}}{k_{1}k_{3}k_{5}k_{7}+k_{1}k_{3}k_{5}k_{9}+k_{1}k_{3}k_{7}k_{9}+k_{1}k_{4}k_{7}k_{9}+k_{1}k_{5}k_{7}k_{9}}$ |
|  | $K_{m}\left( co2 \right)= \frac{k_{2}k_{4}k_{9}+k_{2}k_{5}k_{9}+k_{3}k_{5}k_{9}}{k_{2}k_{4}k_{10}+k_{2}k_{5}k_{10}+k_{3}k_{5}k_{10}}$ |
|  | $K_{m}\left( nadh \right)= \frac{k_{2}k_{4}k_{7}+k_{2}k_{5}k_{7}+k_{3}k_{5}k_{7}}{k_{2}k_{4}k_{6}}$ |
|  | $K_{m}\left( pyr,nadh \right)= \frac{k_{2}k_{4}k_{7}k_{9}+k_{2}k_{5}k_{7}k_{9}+k_{3}k_{5}k_{7}k_{9}}{k_{2}k_{4}k_{6}k_{8}}$ |
|  | $K_{m}\left( co2,nadh \right)= \frac{k_{2}k_{4}k_{7}k_{9}+k_{2}k_{5}k_{7}k_{9}+k_{3}k_{5}k_{7}k_{9}}{k_{2}k_{4}k_{6}k_{10}}$ |
|  | $K_{m}\left( co2,pyr \right)= \frac{k_{2}k_{4}k_{7}k_{9}+k_{2}k_{5}k_{7}k_{9}+k_{3}k_{5}k_{7}k_{9}}{k_{2}k_{4}k_{8}k_{10}+k_{2}k_{5}k_{8}k_{10}+k_{3}k_{5}k_{8}k_{10}}$ |
|  | $K_{m}\left( mal,nadh \right)= \frac{k_{2}k_{4}k_{7}+k_{2}k_{5}k_{7}+k_{3}k_{5}k_{7}}{k_{1}k_{3}k_{6}+k_{1}k_{4}k_{6}}$ |
|  | $K_{m}\left( mal,pyr \right)= \frac{k_{2}k_{4}k_{7}k_{9}+k_{2}k_{5}k_{7}k_{9}+k_{3}k_{5}k_{7}k_{9}}{k_{1}k_{3}k_{5}k_{8}}$ |
|  | $K_{m}\left( mal,pyr,nadh \right)= \frac{k_{2}k_{4}k_{7}k_{9}+k_{2}k_{5}k_{7}k_{9}+k_{3}k_{5}k_{7}k_{9}}{k_{1}k_{3}k_{6}k_{8}+k_{1}k_{4}k_{6}k_{8}}$ |
|  | $K_{m}\left( co2,pyr,nadh \right)= \frac{k_{2}k_{4}k_{7}k_{9}+k_{2}k_{5}k_{7}k_{9}+k_{3}k_{5}k_{7}k_{9}}{k_{2}k_{6}k_{8}k_{10}+k_{3}k_{6}k_{8}k_{10}+k_{4}k_{6}k_{8}k_{10}}$ |
| PPC | $K_{m}\left( pep \right)= \frac{k_{2}k_{4}k_{6}+k_{2}k_{4}k_{7}+k_{2}k_{5}k_{7}}{k_{1}k_{4}k_{6}+k_{1}k_{4}k_{7}+k_{1}k_{5}k_{7}}$ |
|  | $K_{m}\left( co2 \right)= \frac{k_{2}k_{4}k_{6}+k_{2}k_{4}k_{7}+k_{2}k_{5}k_{7}}{k_{3}k_{5}k_{7}}$ |
|  | $K_{m}\left( oac \right)= \frac{k_{2}k_{4}k_{6}+k_{2}k_{4}k_{7}+k_{2}k_{5}k_{7}}{k_{2}k_{4}k_{8}+k_{2}k_{5}k_{8}+k_{2}k_{6}k_{8}+k_{4}k_{6}k_{8}}$ |
|  | $kI\left( suc \right)= {kI}_{1}$ |
|  | $kI\left( cit \right)= {kI}_{2}$ |
|  | $kI\left( fum \right)= {kI}_{3}$ |
|  | $kI\left( cys \right)= {kI}_{4}$ |
|  | $kI\left( mal \right)= {kI}_{5}$ |
|  | $K_{m}\left( pep,co2 \right)= \frac{k_{2}k_{4}k_{6}+k_{2}k_{4}k_{7}+k_{2}k_{5}k_{7}}{k_{1}k_{3}k_{5}+k_{1}k_{3}k_{6}+k_{1}k_{3}k_{7}}$ |
|  | $K_{m}\left( pep,mal \right)= \frac{{kI}_{6}(k_{2}k_{4}k_{6}+k_{2}k_{4}k_{7}+k_{2}k_{5}k_{7})}{k_{1}k_{4}k_{6}+k_{1}k_{4}k_{7}+k_{1}k_{5}k_{7}}$ |
|  | $K_{m}\left( co2,oac \right)= \frac{k_{2}k_{4}k_{6}+k_{2}k_{4}k_{7}+k_{2}k_{5}k_{7}}{k_{3}k_{5}k_{8}+k_{3}k_{6}k_{8}}$ |
|  | $K_{m}\left( co2,suc \right)= \frac{{kI}_{1}(k_{2}k_{4}k_{6}+k_{2}k_{4}k_{7}+k_{2}k_{5}k_{7})}{k_{3}k_{5}k_{7}}$ |
|  | $K_{m}\left( co2,cit \right)= \frac{{kI}_{2}(k_{2}k_{4}k_{6}+k_{2}k_{4}k_{7}+k_{2}k_{5}k_{7})}{k_{3}k_{5}k_{7}}$ |
|  | $K_{m}\left( co2,fum \right)= \frac{{kI}_{3}(k_{2}k_{4}k_{6}+k_{2}k_{4}k_{7}+k_{2}k_{5}k_{7})}{k_{3}k_{5}k_{7}}$ |
|  | $K_{m}\left( co2,cys \right)= \frac{{kI}_{4}(k_{2}k_{4}k_{6}+k_{2}k_{4}k_{7}+k_{2}k_{5}k_{7})}{k_{3}k_{5}k_{7}}$ |
|  | $K_{m}\left( co2,mal \right)= \frac{{kI}_{5}(k_{2}k_{4}k_{6}+k_{2}k_{4}k_{7}+k_{2}k_{5}k_{7})}{k_{3}k_{5}k_{7}}$ |
|  | $K_{m}\left( oac,mal \right)= \frac{{kI}_{6}(k_{2}k_{4}k_{6}+k_{2}k_{4}k_{7}+k_{2}k_{5}k_{7})}{k_{4}k_{6}k_{8}}$ |
| PPCK | $K_{m}\left( atp \right)= \frac{k_{2}k_{4}k_{6}+k_{2}k_{4}k_{7}+k_{2}k_{5}k_{7}}{k_{1}k_{4}k_{6}+k_{1}k_{4}k_{7}+k_{1}k_{5}k_{7}}$ |
|  | $K_{m}\left( oac \right)= \frac{k_{2}k_{4}k_{6}+k_{2}k_{4}k_{7}+k_{2}k_{5}k_{7}}{k_{3}k_{5}k_{7}}$ |
|  | $K_{m}\left( co2 \right)= \frac{k_{2}k_{4}k_{6}k_{9}+k_{2}k_{4}k_{7}k_{9}+k_{2}k_{5}k_{7}k_{9}}{k_{2}k_{4}k_{6}k_{10}+k_{2}k_{4}k_{7}k_{10}+k_{2}k_{5}k_{7}k_{10}}$ |
|  | $K_{m}\left( pep \right)= \frac{k_{2}k_{4}k_{6}k_{9}+k_{2}k_{4}k_{7}k_{9}+k_{2}k_{5}k_{7}k_{9}}{k_{2}k_{4}k_{6}k_{8}}$ |
|  | $kI\left( f6p \right)= {kI}_{1}$ |
|  | $kI\left( fdp \right)= {kI}_{2}$ |
|  | $kI\left( atp \right)= {kI}_{5}$ |
|  | $kI\left( pep \right)= {kI}_{7}$ |
|  | $K_{m}\left( atp,oac \right)= \frac{k_{2}k_{4}k_{6}k_{9}+k_{2}k_{4}k_{7}k_{9}+k_{2}k_{5}k_{7}k_{9}}{k_{1}k_{3}k_{5}k_{7}+k_{1}k_{3}k_{5}k_{9}+k_{1}k_{3}k_{6}k_{9}+k_{1}k_{3}k_{7}k_{9}}$ |
|  | $K_{m}\left( atp,pep \right)= \frac{k_{2}k_{4}k_{6}k_{9}+k_{2}k_{4}k_{7}k_{9}+k_{2}k_{5}k_{7}k_{9}}{k_{1}k_{4}k_{6}k_{8}}$ |
|  | $K_{m}\left( oac,nadh \right)= \frac{{kI}_{3}(k_{2}k_{4}k_{6}+k_{2}k_{4}k_{7}+k_{2}k_{5}k_{7})}{k_{1}k_{4}k_{6}+k_{1}k_{4}k_{7}+k_{1}k_{5}k_{6}}$ |
|  | $K_{m}\left( oac,dhap \right)= \frac{{kI}_{4}(k_{2}k_{4}k_{6}+k_{2}k_{4}k_{7}+k_{2}k_{5}k_{7})}{k_{1}k_{4}k_{6}+k_{1}k_{4}k_{7}+k_{1}k_{5}k_{6}}$ |
|  | $K_{m}\left( oac,atp \right)= \frac{{kI}_{6}\left( k_{2}k_{4}k_{6}+k_{2}k_{4}k_{7}+k_{2}k_{5}k_{7} \right)}{k_{1}k_{4}k_{6}+k_{1}k_{4}k_{7}+k_{1}k_{5}k_{6}}$ |
|  | $K_{m}\left( oac,pep \right)= \frac{{kI}_{8}(k_{2}k_{4}k_{6}+k_{2}k_{4}k_{7}+k_{2}k_{5}k_{7})}{k_{1}k_{4}k_{6}+k_{1}k_{4}k_{7}+k_{1}k_{5}k_{6}}$ |
|  | $K_{m}\left( oac,co2 \right)= \frac{k_{2}k_{4}k_{6}k_{9}+k_{2}k_{4}k_{7}k_{9}+k_{2}k_{5}k_{7}k_{9}}{k_{3}k_{5}k_{7}k_{10}}$ |
|  | $K_{m}\left( oac,f6p \right)= \frac{{kI}_{1}(k_{2}k_{4}k_{6}+k_{2}k_{4}k_{7}+k_{2}k_{5}k_{7})}{k_{3}k_{5}k_{7}}$ |
|  | $K_{m}\left( oac,fdp \right)= \frac{{kI}_{2}(k_{2}k_{4}k_{6}+k_{2}k_{4}k_{7}+k_{2}k_{5}k_{7})}{k_{3}k_{5}k_{7}}$ |
|  | $K_{m}\left( oac,atp \right)= \frac{{kI}_{5}(k_{2}k_{4}k_{6}+k_{2}k_{4}k_{7}+k_{2}k_{5}k_{7})}{k_{3}k_{5}k_{7}}$ |
|  | $K_{m}\left( oac,pep \right)= \frac{{kI}_{7}(k_{2}k_{4}k_{6}+k_{2}k_{4}k_{7}+k_{2}k_{5}k_{7})}{k_{3}k_{5}k_{7}}$ |
|  | $K_{m}\left( co2,pep \right)= \frac{k_{2}k_{4}k_{6}k_{9}+k_{2}k_{4}k_{7}k_{9}+k_{2}k_{5}k_{7}k_{9}}{k_{2}k_{4}k_{8}k_{10}+k_{2}k_{5}k_{8}k_{10}+k_{2}k_{6}k_{8}k_{10}+k_{4}k_{6}k_{8}k_{10}}$ |
|  | $K_{m}\left( pep,f6p \right)= \frac{{kI}_{1}(k_{2}k_{4}k_{6}k_{9}+k_{2}k_{4}k_{7}k_{9}+k_{2}k_{5}k_{7}k_{9})}{k_{2}k_{4}k_{6}k_{8}}$ |
|  | $K_{m}\left( pep,fdp \right)= \frac{{kI}_{2}(k_{2}k_{4}k_{6}k_{9}+k_{2}k_{4}k_{7}k_{9}+k_{2}k_{5}k_{7}k_{9})}{k_{2}k_{4}k_{6}k_{8}}$ |
|  | $K_{m}\left( pep,atp \right)= \frac{{kI}_{5}(k_{2}k_{4}k_{6}k_{9}+k_{2}k_{4}k_{7}k_{9}+k_{2}k_{5}k_{7}k_{9})}{k_{2}k_{4}k_{6}k_{8}}$ |
|  | $K_{m}\left( pep,pep \right)= \frac{{kI}_{7}(k_{2}k_{4}k_{6}k_{9}+k_{2}k_{4}k_{7}k_{9}+k_{2}k_{5}k_{7}k_{9})}{k_{2}k_{4}k_{6}k_{8}}$ |
|  | $K_{m}\left( atp,oac,pep \right)= \frac{k_{2}k_{4}k_{6}k_{9}+k_{2}k_{4}k_{7}k_{9}+k_{2}k_{5}k_{7}k_{9}}{k_{1}k_{3}k_{5}k_{8}+k_{1}k_{3}k_{6}k_{8}}$ |
|  | $K_{m}\left( atp,pep,nadh \right)= \frac{{kI}_{3}(k_{2}k_{4}k_{6}k_{9}+k_{2}k_{4}k_{7}k_{9}+k_{2}k_{5}k_{7}k_{9})}{k_{1}k_{4}k_{6}k_{8}}$ |
|  | $K_{m}\left( atp,pep,dhap \right)= \frac{{kI}_{4}(k_{2}k_{4}k_{6}k_{9}+k_{2}k_{4}k_{7}k_{9}+k_{2}k_{5}k_{7}k_{9})}{k_{1}k_{4}k_{6}k_{8}}$ |
|  | $K_{m}\left( atp,pep,atp \right)= \frac{{kI}_{6}(k_{2}k_{4}k_{6}k_{9}+k_{2}k_{4}k_{7}k_{9}+k_{2}k_{5}k_{7}k_{9})}{k_{1}k_{4}k_{6}k_{8}}$ |
|  | $K_{m}\left( atp,pep,pep \right)= \frac{{kI}_{8}(k_{2}k_{4}k_{6}k_{9}+k_{2}k_{4}k_{7}k_{9}+k_{2}k_{5}k_{7}k_{9})}{k_{1}k_{4}k_{6}k_{8}}$ |
|  | $K_{m}\left( oac,co2,pep \right)= \frac{k_{2}k_{4}k_{6}k_{9}+k_{2}k_{4}k_{7}k_{9}+k_{2}k_{5}k_{7}k_{9}}{k_{3}k_{5}k_{8}k_{10}+k_{3}k_{6}k_{8}k_{10}}$ |
|  | $K_{m}\left( co2,pep,nadh \right)= \frac{{kI}_{3}(k_{2}k_{4}k_{6}k_{9}+k_{2}k_{4}k_{7}k_{9}+k_{2}k_{5}k_{7}k_{9})}{k_{4}k_{6}k_{8}k_{10}}$ |
|  | $K_{m}\left( co2,pep,dhap \right)= \frac{{kI}_{4}(k_{2}k_{4}k_{6}k_{9}+k_{2}k_{4}k_{7}k_{9}+k_{2}k_{5}k_{7}k_{9})}{k_{4}k_{6}k_{8}k_{10}}$ |
|  | $K_{m}\left( co2,pep,atp \right)= \frac{{kI}_{6}(k_{2}k_{4}k_{6}k_{9}+k_{2}k_{4}k_{7}k_{9}+k_{2}k_{5}k_{7}k_{9})}{k_{4}k_{6}k_{8}k_{10}}$ |
|  | $K_{m}\left( co2,pep,pep \right)= \frac{{kI}_{8}(k_{2}k_{4}k_{6}k_{9}+k_{2}k_{4}k_{7}k_{9}+k_{2}k_{5}k_{7}k_{9})}{k_{4}k_{6}k_{8}k_{10}}$ |
| PTAr/ACKr | $K_{m}\left( accoa \right)= \frac{k_{2}k_{4}k_{7}+k_{2}k_{5}k_{7}+k_{3}k_{5}k_{7}}{k_{1}k_{3}k_{5}+k_{1}k_{3}k_{7}+k_{1}k_{4}k_{7}+k_{1}k_{5}k_{7}}$ |
|  | $K_{m}\left( ac \right)= \frac{k_{2}k_{4}k_{7}+k_{2}k_{5}k_{7}+k_{3}k_{5}k_{7}}{k_{2}k_{4}k_{8}+k_{2}k_{5}k_{8}+k_{3}k_{5}k_{8}}$ |
|  | $K_{m}\left( atp \right)= \frac{k_{2}k_{4}k_{7}+k_{2}k_{5}k_{7}+k_{3}k_{5}k_{7}}{k_{2}k_{4}k_{6}}$ |
|  | $K_{m}\left( accoa,atp \right)= \frac{k_{2}k_{4}k_{7}+k_{2}k_{5}k_{7}+k_{3}k_{5}k_{7}}{k_{1}k_{3}k_{6}+k_{1}k_{4}k_{6}}$ |
|  | $K_{m}\left( ac,atp \right)= \frac{k_{2}k_{4}k_{7}+k_{2}k_{5}k_{7}+k_{3}k_{5}k_{7}}{k_{2}k_{6}k_{8}+k_{3}k_{6}k_{8}+k_{4}k_{6}k_{8}}$ |
| GLUDy | $K_{m}\left( nadph \right)= \frac{k_{2}k_{6}k_{8}+k_{2}k_{6}k_{9}+k_{2}k_{7}k_{9}}{k_{1}k_{6}k_{8}+k_{1}k_{6}k_{9}+k_{1}k_{7}k_{9}}$ |
|  | $K_{m}\left( nh3 \right)= \frac{k_{2}k_{4}k_{6}k_{8}+k_{2}k_{4}k_{6}k_{9}+k_{2}k_{4}k_{7}k_{9}}{k_{2}k_{5}k_{7}k_{9}}$ |
|  | $K_{m}\left( glu \right)= \frac{k_{2}k_{4}k_{6}k_{8}+k_{2}k_{4}k_{6}k_{9}+k_{2}k_{4}k_{7}k_{9}}{k_{2}k_{4}k_{6}k_{10}+k_{2}k_{4}k_{7}k_{10}+k_{2}k_{4}k_{8}k_{10}+k_{2}k_{6}k_{8}k_{10}+k_{4}k_{6}k_{8}k_{10}}$ |
|  | $K_{m}\left( nh3,glu \right)= \frac{k_{2}k_{4}k_{6}k_{8}+k_{2}k_{4}k_{6}k_{9}+k_{2}k_{4}k_{7}k_{9}}{k_{2}k_{5}k_{7}k_{10}+k_{2}k_{5}k_{8}k_{10}}$ |
|  | $K_{m}\left( akg,glu \right)= \frac{k_{2}k_{4}k_{6}k_{8}+k_{2}k_{4}k_{6}k_{9}+k_{2}k_{4}k_{7}k_{9}}{k_{3}k_{6}k_{8}k_{10}}$ |
|  | $K_{m}\left( akg,nh3 \right)= \frac{k_{2}k_{4}k_{6}k_{8}+k_{2}k_{4}k_{6}k_{9}+k_{2}k_{4}k_{7}k_{9}}{k_{3}k_{5}k_{7}k_{9}}$ |
|  | $K_{m}\left( nadph,nh3 \right)= \frac{k_{2}k_{4}k_{6}k_{8}+k_{2}k_{4}k_{6}k_{9}+k_{2}k_{4}k_{7}k_{9}}{k_{1}k_{5}k_{7}k_{9}}$ |
|  | $K_{m}\left( nadph,akg \right)= \frac{k_{2}k_{4}k_{6}k_{8}+k_{2}k_{4}k_{6}k_{9}+k_{2}k_{4}k_{7}k_{9}}{k_{1}k_{3}k_{6}k_{8}+k_{1}k_{3}k_{6}k_{9}+k_{1}k_{3}k_{7}k_{9}}$ |
|  | $K_{m}\left( nadph,akg,nh3 \right)= \frac{k_{2}k_{4}k_{6}k_{8}+k_{2}k_{4}k_{6}k_{9}+k_{2}k_{4}k_{7}k_{9}}{k_{1}k_{3}k_{5}k_{7}+k_{1}k_{3}k_{5}k_{8}+k_{1}k_{3}k_{5}k_{9}}$ |
|  | $K_{m}\left( akg,nh3,glu \right)= \frac{k_{2}k_{4}k_{6}k_{8}+k_{2}k_{4}k_{6}k_{9}+k_{2}k_{4}k_{7}k_{9}}{k_{3}k_{5}k_{7}k_{10}+k_{3}k_{5}k_{8}k_{10}}$ |
| GLNS | $K_{m}\left( atp \right)= \frac{k_{2}k_{6}k_{8}+k_{2}k_{6}k_{9}+k_{2}k_{7}k_{9}}{k_{1}k_{6}k_{8}+k_{1}k_{6}k_{9}+k_{1}k_{7}k_{9}}$ |
|  | $K_{m}\left( glu \right)= \frac{k_{2}k_{4}k_{6}k_{8}+k_{2}k_{4}k_{6}k_{9}+k_{2}k_{4}k_{7}k_{9}}{k_{2}k_{5}k_{7}k_{9}}$ |
|  | $K_{m}\left( gln \right)= \frac{k_{2}k_{4}k_{6}k_{8}+k_{2}k_{4}k_{6}k_{9}+k_{2}k_{4}k_{7}k_{9}}{k_{2}k_{4}k_{6}k_{10}+k_{2}k_{4}k_{7}k_{10}+k_{2}k_{4}k_{8}k_{10}+k_{2}k_{6}k_{8}k_{10}+k_{4}k_{6}k_{8}k_{10}}$ |
|  | $K_{m}\left( glu,gln \right)= \frac{k_{2}k_{4}k_{6}k_{8}+k_{2}k_{4}k_{6}k_{9}+k_{2}k_{4}k_{7}k_{9}}{k_{2}k_{5}k_{7}k_{10}+k_{2}k_{5}k_{8}k_{10}}$ |
|  | $K_{m}\left( nh3,gln \right)= \frac{k_{2}k_{4}k_{6}k_{8}+k_{2}k_{4}k_{6}k_{9}+k_{2}k_{4}k_{7}k_{9}}{k_{3}k_{6}k_{8}k_{10}}$ |
|  | $K_{m}\left( atp,glu \right)= \frac{k_{2}k_{4}k_{6}k_{8}+k_{2}k_{4}k_{6}k_{9}+k_{2}k_{4}k_{7}k_{9}}{k_{1}k_{5}k_{7}k_{9}}$ |
|  | $K_{m}\left( atp,nh3 \right)= \frac{k_{2}k_{4}k_{6}k_{8}+k_{2}k_{4}k_{6}k_{9}+k_{2}k_{4}k_{7}k_{9}}{k_{1}k_{3}k_{6}k_{8}+k_{1}k_{3}k_{6}k_{9}+k_{1}k_{3}k_{7}k_{9}}$ |
|  | $K_{m}\left( atp,nh3,glu \right)= \frac{k_{2}k_{4}k_{6}k_{8}+k_{2}k_{4}k_{6}k_{9}+k_{2}k_{4}k_{7}k_{9}}{k_{1}k_{3}k_{5}k_{7}+k_{1}k_{3}k_{5}k_{8}+k_{1}k_{3}k_{5}k_{9}}$ |
|  | $K_{m}\left( nh3,glu,gln \right)= \frac{k_{2}k_{4}k_{6}k_{8}+k_{2}k_{4}k_{6}k_{9}+k_{2}k_{4}k_{7}k_{9}}{k_{3}k_{5}k_{7}k_{10}+k_{3}k_{5}k_{8}k_{10}}$ |
| ASPTA | $K_{m}\left( oac \right)= \frac{k_{2}k_{4}k_{6}+k_{2}k_{4}k_{7}+k_{2}k_{5}k_{7}}{k_{1}k_{4}k_{6}+k_{1}k_{4}k_{7}+k_{1}k_{5}k_{7}}$ |
|  | $K_{m}\left( glu \right)= \frac{k_{2}k_{4}k_{6}+k_{2}k_{4}k_{7}+k_{2}k_{5}k_{7}}{k_{3}k_{5}k_{7}}$ |
|  | $K_{m}\left( asp \right)= \frac{k_{2}k_{4}k_{6}k_{9}+k_{2}k_{4}k_{7}k_{9}+k_{2}k_{5}k_{7}k_{9}}{k_{2}k_{4}k_{6}k_{10}+k_{2}k_{4}k_{7}k_{10}+k_{2}k_{5}k_{7}k_{10}}$ |
|  | $K_{m}\left( akg \right)= \frac{k_{2}k_{4}k_{6}k_{9}+k_{2}k_{4}k_{7}k_{9}+k_{2}k_{5}k_{7}k_{9}}{k_{2}k_{4}k_{6}k_{8}}$ |
|  | $K_{m}\left( asp,akg \right)= \frac{k_{2}k_{4}k_{6}k_{9}+k_{2}k_{4}k_{7}k_{9}+k_{2}k_{5}k_{7}k_{9}}{k_{2}k_{4}k_{8}k_{10}+k_{2}k_{5}k_{8}k_{10}+k_{2}k_{6}k_{8}k_{10}+k_{4}k_{6}k_{8}k_{10}}$ |
|  | $K_{m}\left( glu,asp \right)= \frac{k_{2}k_{4}k_{6}k_{9}+k_{2}k_{4}k_{7}k_{9}+k_{2}k_{5}k_{7}k_{9}}{k_{3}k_{5}k_{7}k_{10}}$ |
|  | $K_{m}\left( oac,akg \right)= \frac{k_{2}k_{4}k_{6}k_{9}+k_{2}k_{4}k_{7}k_{9}+k_{2}k_{5}k_{7}k_{9}}{k_{1}k_{4}k_{6}k_{8}}$ |
|  | $K_{m}\left( oac,glu \right)= \frac{k_{2}k_{4}k_{6}k_{9}+k_{2}k_{4}k_{7}k_{9}+k_{2}k_{5}k_{7}k_{9}}{k_{1}k_{3}k_{5}k_{7}+k_{1}k_{3}k_{5}k_{9}+k_{1}k_{3}k_{6}k_{9}+k_{1}k_{3}k_{7}k_{9}}$ |
|  | $K_{m}\left( glu,asp,akg \right)= \frac{k_{2}k_{4}k_{6}k_{9}+k_{2}k_{4}k_{7}k_{9}+k_{2}k_{5}k_{7}k_{9}}{k_{3}k_{5}k_{8}k_{10}+k_{3}k_{6}k_{8}k_{10}}$ |
|  | $K_{m}\left( oac,glu,akg \right)= \frac{k_{2}k_{4}k_{6}k_{9}+k_{2}k_{4}k_{7}k_{9}+k_{2}k_{5}k_{7}k_{9}}{k_{1}k_{3}k_{5}k_{8}+k_{1}k_{3}k_{6}k_{8}}$ |
| GHMT2r | $K_{m}\left( ser \right)= \frac{k_{2}k_{4}k_{7}+k_{2}k_{5}k_{7}+k_{3}k_{5}k_{7}}{k_{1}k_{3}k_{5}+k_{1}k_{3}k_{7}+k_{1}k_{4}k_{7}+k_{1}k_{5}k_{7}}$ |
|  | $K_{m}\left( meethf \right)= \frac{k_{2}k_{4}k_{7}+k_{2}k_{5}k_{7}+k_{3}k_{5}k_{7}}{k_{2}k_{4}k_{8}+k_{2}k_{5}k_{8}+k_{3}k_{5}k_{8}}$ |
|  | $K_{m}\left( gly \right)= \frac{k_{2}k_{4}k_{7}+k_{2}k_{5}k_{7}+k_{3}k_{5}k_{7}}{k_{2}k_{4}k_{6}}$ |
|  | $K_{m}\left( ser,gly \right)= \frac{k_{2}k_{4}k_{7}+k_{2}k_{5}k_{7}+k_{3}k_{5}k_{7}}{k_{1}k_{3}k_{6}+k_{1}k_{4}k_{6}}$ |
|  | $K_{m}\left( meethf,gly \right)= \frac{k_{2}k_{4}k_{7}+k_{2}k_{5}k_{7}+k_{3}k_{5}k_{7}}{k_{2}k_{6}k_{8}+k_{3}k_{6}k_{8}+k_{4}k_{6}k_{8}}$ |
| GLYCL | $K_{m}\left( gly \right)= \frac{k_{2}k_{4}k_{7}k_{9}k_{11}+k_{2}k_{5}k_{7}k_{9}k_{11}+k_{3}k_{5}k_{7}k_{9}k_{11}}{k_{1}k_{3}k_{5}k_{7}k_{9}+k_{1}k_{3}k_{5}k_{7}k_{11}+k_{1}k_{3}k_{7}k_{9}k_{11}+k_{1}k_{4}k_{7}k_{9}k_{11}+k_{1}k_{5}k_{7}k_{9}k_{11}}$ |
|  | $K_{m}\left( meethf \right)= \frac{k_{2}k_{4}k_{11}+k_{2}k_{5}k_{11}+k_{3}k_{5}k_{11}}{k_{2}k_{4}k_{12}+k_{2}k_{5}k_{12}+k_{3}k_{4}k_{12}}$ |
|  | $K_{m}\left( nadh \right)= \frac{k_{2}k_{4}k_{7}+k_{2}k_{5}k_{7}+k_{3}k_{5}k_{7}}{k_{2}k_{4}k_{6}}$ |
|  | $K_{m}\left( co2,nadh \right)= \frac{k_{2}k_{4}k_{7}k_{9}+k_{2}k_{5}k_{7}k_{9}+k_{3}k_{5}k_{7}k_{9}}{k_{2}k_{4}k_{6}k_{8}}$ |
|  | $K_{m}\left( meethf,nadh \right)= \frac{k_{2}k_{4}k_{7}k_{11}+k_{2}k_{5}k_{7}k_{11}+k_{3}k_{5}k_{7}k_{11}}{k_{2}k_{4}k_{6}k_{12}}$ |
|  | $K_{m}\left( meethf,nh3 \right)= \frac{k_{2}k_{4}k_{9}k_{11}+k_{2}k_{5}k_{9}k_{11}+k_{3}k_{5}k_{9}k_{11}}{k_{2}k_{4}k_{10}k_{12}+k_{2}k_{5}k_{10}k_{12}+k_{3}k_{5}k_{10}k_{12}}$ |
|  | $K_{m}\left( gly,nadh \right)= \frac{k_{2}k_{4}k_{7}+k_{2}k_{5}k_{7}+k_{3}k_{5}k_{7}}{k_{1}k_{3}k_{6}+k_{1}k_{4}k_{6}}$ |
|  | $K_{m}\left( gly,co2 \right)= \frac{k_{2}k_{4}k_{7}k_{9}+k_{2}k_{5}k_{7}k_{9}+k_{3}k_{5}k_{7}k_{9}}{k_{1}k_{3}k_{5}k_{8}}$ |
|  | $K_{m}\left( gly,nh3 \right)= \frac{k_{2}k_{4}k_{9}k_{11}+k_{2}k_{5}k_{9}k_{11}+k_{3}k_{5}k_{9}k_{11}}{k_{1}k_{3}k_{5}k_{10}}$ |
|  | $K_{m}\left( nh3,co2,nadh \right)= \frac{k_{2}k_{4}k_{7}k_{9}k_{11}+k_{2}k_{5}k_{7}k_{9}k_{11}+k_{3}k_{5}k_{7}k_{9}k_{11}}{k_{2}k_{4}k_{6}k_{8}k_{10}}$ |
|  | $K_{m}\left( meethf,co2,nadh \right)= \frac{k_{2}k_{4}k_{7}k_{9}k_{11}+k_{2}k_{5}k_{7}k_{9}k_{11}+k_{3}k_{5}k_{7}k_{9}k_{11}}{k_{2}k_{4}k_{6}k_{8}k_{12}}$ |
|  | $K_{m}\left( meethf,nh3,nadh \right)= \frac{k_{2}k_{4}k_{7}k_{9}k_{11}+k_{2}k_{5}k_{7}k_{9}k_{11}+k_{3}k_{5}k_{7}k_{9}k_{11}}{k_{2}k_{4}k_{6}k_{10}k_{12}}$ |
|  | $K_{m}\left( meethf,nh3,co2 \right)= \frac{k_{2}k_{4}k_{7}k_{9}k_{11}+k_{2}k_{5}k_{7}k_{9}k_{11}+k_{3}k_{5}k_{7}k_{9}k_{11}}{k_{2}k_{4}k_{8}k_{10}k_{12}+k_{2}k_{5}k_{8}k_{10}k_{12}+k_{3}k_{4}k_{8}k_{10}k_{12}}$ |
|  | $K_{m}\left( gly,co2,nadh \right)= \frac{k_{2}k_{4}k_{7}k_{9}+k_{2}k_{5}k_{7}k_{9}+k_{3}k_{5}k_{7}k_{9}}{k_{1}k_{3}k_{6}k_{8}+k_{1}k_{4}k_{6}k_{8}}$ |
|  | $K_{m}\left( gly,nh3,co2 \right)= \frac{k_{2}k_{4}k_{7}k_{9}k_{11}+k_{2}k_{5}k_{7}k_{9}k_{11}+k_{3}k_{5}k_{7}k_{9}k_{11}}{k_{1}k_{3}k_{5}k_{8}k_{10}}$ |
|  | $K_{m}\left( gly,nh3,co2,nadh \right)= \frac{k_{2}k_{4}k_{7}k_{9}k_{11}+k_{2}k_{5}k_{7}k_{9}k_{11}+k_{3}k_{5}k_{7}k_{9}k_{11}}{k_{1}k_{3}k_{6}k_{8}k_{10}+k_{1}k_{4}k_{6}k_{8}k_{10}}$ |
|  | $K_{m}\left( meethf,nh3,co2,nadh \right)= \frac{k_{2}k_{4}k_{7}k_{9}k_{11}+k_{2}k_{5}k_{7}k_{9}k_{11}+k_{3}k_{5}k_{7}k_{9}k_{11}}{k_{2}k_{6}k_{8}k_{10}k_{12}+k_{3}k_{6}k_{8}k_{10}k_{12}+k_{4}k_{6}k_{8}k_{10}k_{12}}$ |
| SERD-L | $K_{m}\left( ser \right)= \frac{k_{2}k_{4}k_{7}+k_{2}k_{5}k_{7}+k_{3}k_{5}k_{7}}{k_{1}k_{3}k_{5}+k_{1}k_{3}k_{7}+k_{1}k_{4}k_{7}+k_{1}k_{5}k_{7}}$ |
|  | $K_{m}\left( nh3 \right)= \frac{k_{2}k_{4}k_{7}+k_{2}k_{5}k_{7}+k_{3}k_{5}k_{7}}{k_{2}k_{4}k_{8}+k_{2}k_{5}k_{8}+k_{3}k_{5}k_{8}}$ |
|  | $K_{m}\left( pyr \right)= \frac{k_{2}k_{4}k_{7}+k_{2}k_{5}k_{7}+k_{3}k_{5}k_{7}}{k_{2}k_{4}k_{6}}$ |
|  | $K_{m}\left( ser,pyr \right)= \frac{k_{2}k_{4}k_{7}+k_{2}k_{5}k_{7}+k_{3}k_{5}k_{7}}{k_{1}k_{3}k_{6}+k_{1}k_{4}k_{6}}$ |
|  | $K_{m}\left( nh3,pyr \right)= \frac{k_{2}k_{4}k_{7}+k_{2}k_{5}k_{7}+k_{3}k_{5}k_{7}}{k_{2}k_{6}k_{8}+k_{3}k_{6}k_{8}+k_{4}k_{6}k_{8}}$ |
| MTHFR2 | $K_{m}\left( nadh \right)= \frac{k_{2}k_{4}k_{6}+k_{2}k_{4}k_{7}+k_{2}k_{5}k_{7}}{k_{1}k_{4}k_{6}+k_{1}k_{4}k_{7}+k_{1}k_{5}k_{7}}$ |
|  | $K_{m}\left( meethf \right)= \frac{k_{2}k_{4}k_{6}+k_{2}k_{4}k_{7}+k_{2}k_{5}k_{7}}{k_{3}k_{5}k_{7}}$ |
|  | $K_{m}\left( methf \right)= \frac{k_{2}k_{4}k_{6}+k_{2}k_{4}k_{7}+k_{2}k_{5}k_{7}}{k_{2}k_{4}k_{8}+k_{2}k_{5}k_{8}+k_{2}k_{6}k_{8}+k_{4}k_{6}k_{8}}$ |
|  | $K_{m}\left( nadh,meethf \right)= \frac{k_{2}k_{4}k_{6}+k_{2}k_{4}k_{7}+k_{2}k_{5}k_{7}}{k_{1}k_{3}k_{5}+k_{1}k_{3}k_{6}+k_{1}k_{3}k_{7}}$ |
|  | $K_{m}\left( meethf,methf \right)= \frac{k_{2}k_{4}k_{6}+k_{2}k_{4}k_{7}+k_{2}k_{5}k_{7}}{k_{3}k_{5}k_{8}+k_{3}k_{6}k_{8}}$ |
| MTHFD | $K_{m}\left( meethf \right)= \frac{k_{2}k_{4}k_{7}+k_{2}k_{5}k_{7}+k_{3}k_{5}k_{7}}{k_{1}k_{3}k_{5}+k_{1}k_{3}k_{7}+k_{1}k_{4}k_{7}+k_{1}k_{5}k_{7}}$ |
|  | $K_{m}\left( fthf \right)= \frac{k_{2}k_{4}k_{7}+k_{2}k_{5}k_{7}+k_{3}k_{5}k_{7}}{k_{2}k_{4}k_{8}+k_{2}k_{5}k_{8}+k_{3}k_{5}k_{8}}$ |
|  | $K_{m}\left( nadph \right)= \frac{k_{2}k_{4}k_{7}+k_{2}k_{5}k_{7}+k_{3}k_{5}k_{7}}{k_{2}k_{4}k_{6}}$ |
|  | $K_{m}\left( meethf,nadph \right)= \frac{k_{2}k_{4}k_{7}+k_{2}k_{5}k_{7}+k_{3}k_{5}k_{7}}{k_{1}k_{3}k_{6}+k_{1}k_{4}k_{6}}$ |
|  | $K_{m}\left( fthf,nadph \right)= \frac{k_{2}k_{4}k_{7}+k_{2}k_{5}k_{7}+k_{3}k_{5}k_{7}}{k_{2}k_{6}k_{8}+k_{3}k_{6}k_{8}+k_{4}k_{6}k_{8}}$ |
| NADTRHD | $K_{m}\left( nadh \right)= \frac{k_{2}k_{4}+k_{2}k_{5}+k_{3}k_{5}}{k_{1}k_{3}+k_{1}k_{4}+k_{1}k_{5}}$ |
|  | $K_{m}\left( nadph \right)= \frac{k_{2}k_{4}+k_{2}k_{5}+k_{3}k_{5}}{k_{2}k_{6}+k_{3}k_{6}+k_{4}k_{6}}$ |
