# Supplementary material for: From Escherichia coli mutant 13C labeling data to a core kinetic model: A kinetic model parameterization pipeline
Source: PLoS Comput Biol. 2019 Sep 10;15(9):e1007319. doi: 10.1371/journal.pcbi.1007319 (PMC6759195; doi:10.1371/journal.pcbi.1007319)
Supplement: S6 File — (DOCX) [file pcbi.1007319.s006.docx]

**S6 File:** $\boldsymbol{Vmax}$ **rate constant expressed as a function of elementary kinetic parameters and enzyme concentrations for central carbon reactions in k-ecoli74**

| Reaction | Parameter |
| --- | --- |
| PGI | $Vmax\left( f \right)= \frac{k_{1}k_{3}k_{5}[PGI]}{k_{1}k_{3}+k_{1}k_{4}+k_{1}k_{5}}$ |
|  | $Vmax\left( r \right)= \frac{k_{2}k_{4}[PGI]}{k_{2}+k_{3}+k_{4}}$ |
| PFK | $Vmax\left( f \right)= \frac{k_{1}k_{3}k_{5}k_{7}[PFK]}{k_{2}k_{4}k_{6}+k_{2}k_{4}k_{7}+k_{2}k_{5}k_{7}}$ |
|  | $Vmax\left( r \right)= \frac{k_{2}k_{4}k_{6}[PFK]}{k_{2}k_{4}+k_{2}k_{5}+k_{2}k_{6}+k_{4}k_{6}}$ |
| FBP | $Vmax\left( f \right)= \frac{k_{1}k_{3}k_{5}[FBP]}{k_{1}k_{3}+k_{1}k_{4}+k_{1}k_{5}}$ |
|  | $Vmax\left( r \right)= \frac{k_{2}k_{4}[FBP]}{k_{2}+k_{3}+k_{4}}$ |
| FBA | $Vmax\left( f \right)= \frac{k_{1}k_{3}k_{5}k_{7}[FBA]}{k_{1}k_{3}k_{5}+k_{1}k_{3}k_{7}+k_{1}k_{4}k_{7}+k_{1}k_{5}k_{7}}$ |
|  | $Vmax\left( r \right)= \frac{k_{2}k_{4}k_{6}k_{8}[FBA]}{k_{2}k_{4}k_{7}+k_{2}k_{5}k_{7}+k_{3}k_{5}k_{7}}$ |
| TPI | $Vmax\left( f \right)= \frac{k_{1}k_{3}k_{5}[TPI]}{k_{1}k_{3}+k_{1}k_{4}+k_{1}k_{5}}$ |
|  | $Vmax\left( r \right)= \frac{k_{2}k_{4}[TPI]}{k_{2}+k_{3}+k_{4}}$ |
| GAPD/PGK | $Vmax\left( f \right)= \frac{k_{1}k_{3}k_{5}k_{7}k_{9}[GAPD-PGK]}{k_{1}k_{3}k_{5}k_{7}+k_{1}k_{3}k_{5}k_{9}+k_{1}k_{3}k_{7}k_{9}+k_{1}k_{4}k_{7}k_{9}+k_{1}k_{5}k_{7}k_{9}}$ |
|  | $Vmax\left( r \right)= \frac{k_{2}k_{4}k_{6}k_{8}k_{10}[GAPD-PGK]}{k_{2}k_{4}k_{7}k_{9}+k_{2}k_{5}k_{7}k_{9}+k_{3}k_{5}k_{7}k_{9}}$ |
| PGM/ENO | $Vmax\left( f \right)= \frac{k_{1}k_{3}k_{5}[PGM-ENO]}{k_{1}k_{3}+k_{1}k_{4}+k_{1}k_{5}}$ |
|  | $Vmax\left( r \right)= \frac{k_{2}k_{4}[PGM-ENO]}{k_{2}+k_{3}+k_{4}}$ |
| PYK | $Vmax\left( f \right)= \frac{k_{1}k_{3}k_{5}k_{7}[PYK]}{k_{1}k_{3}k_{5}+k_{1}k_{3}k_{7}+k_{1}k_{4}k_{7}+k_{1}k_{5}k_{7}}$ |
|  | $Vmax\left( r \right)= \frac{k_{2}k_{4}k_{6}k_{8}[PYK]}{k_{2}k_{4}k_{7}+k_{2}k_{5}k_{7}+k_{3}k_{5}k_{7}}$ |
| G6PDH2r | $Vmax\left( f \right)= \frac{k_{1}k_{3}k_{5}k_{7}[G6PDH2r]}{k_{1}k_{3}k_{5}+k_{1}k_{3}k_{7}+k_{1}k_{4}k_{7}+k_{1}k_{5}k_{7}}$ |
|  | $Vmax\left( r \right)= \frac{k_{2}k_{4}k_{6}k_{8}[G6PDH2r]}{k_{2}k_{4}k_{7}+k_{2}k_{5}k_{7}+k_{3}k_{5}k_{7}}$ |
| GND | $Vmax\left( f \right)= \frac{k_{1}k_{3}k_{5}k_{7}k_{9}[GND]}{k_{1}k_{3}k_{5}k_{7}+k_{1}k_{3}k_{5}k_{9}+k_{1}k_{3}k_{7}k_{9}+k_{1}k_{4}k_{7}k_{9}+k_{1}k_{5}k_{7}k_{9}}$ |
|  | $Vmax\left( r \right)= \frac{k_{2}k_{4}k_{6}k_{8}k_{10}[GND]}{k_{2}k_{4}k_{7}k_{9}+k_{2}k_{5}k_{7}k_{9}+k_{3}k_{5}k_{7}k_{9}}$ |
| RPE | $Vmax\left( f \right)= \frac{k_{1}k_{3}k_{5}[RPE]}{k_{1}k_{3}+k_{1}k_{4}+k_{1}k_{5}}$ |
|  | $Vmax\left( r \right)= \frac{k_{2}k_{4}[RPE]}{k_{2}+k_{3}+k_{4}}$ |
| RPI | $Vmax\left( f \right)= \frac{k_{1}k_{3}k_{5}[RPI]}{k_{1}k_{3}+k_{1}k_{4}+k_{1}k_{5}}$ |
|  | $Vmax\left( r \right)= \frac{k_{2}k_{4}[RPI]}{k_{2}+k_{3}+k_{4}}$ |
| TKThlf1 | $Vmax\left( f \right)= \frac{k_{1}k_{3}k_{5}k_{7}[TKThlf1]}{k_{1}k_{3}k_{5}+k_{1}k_{3}k_{7}+k_{1}k_{4}k_{7}+k_{1}k_{5}k_{7}}$ |
|  | $Vmax\left( r \right)= \frac{k_{2}k_{4}k_{6}k_{8}[TKThlf1]}{k_{2}k_{4}k_{7}+k_{2}k_{5}k_{7}+k_{3}k_{5}k_{7}}$ |
| TKThlf2 | $Vmax\left( f \right)= \frac{k_{1}k_{3}k_{5}k_{7}[TKThlf2]}{k_{1}k_{3}k_{5}+k_{1}k_{3}k_{7}+k_{1}k_{4}k_{7}+k_{1}k_{5}k_{7}}$ |
|  | $Vmax\left( r \right)= \frac{k_{2}k_{4}k_{6}k_{8}[TKThlf2]}{k_{2}k_{4}k_{7}+k_{2}k_{5}k_{7}+k_{3}k_{5}k_{7}}$ |
| TKThlf3 | $Vmax\left( f \right)= \frac{k_{1}k_{3}k_{5}k_{7}[TKThlf3]}{k_{1}k_{3}k_{5}+k_{1}k_{3}k_{7}+k_{1}k_{4}k_{7}+k_{1}k_{5}k_{7}}$ |
|  | $Vmax\left( r \right)= \frac{k_{2}k_{4}k_{6}k_{8}[TKThlf3]}{k_{2}k_{4}k_{7}+k_{2}k_{5}k_{7}+k_{3}k_{5}k_{7}}$ |
| TALA | $Vmax\left( f \right)= \frac{k_{1}k_{3}k_{5}k_{7}[TALA]}{k_{2}k_{4}k_{6}+k_{2}k_{4}k_{7}+k_{2}k_{5}k_{7}}$ |
|  | $Vmax\left( r \right)= \frac{k_{2}k_{4}k_{6}k_{8}k_{10}[TALA]}{k_{2}k_{4}k_{6}k_{9}+k_{2}k_{4}k_{7}k_{9}+k_{2}k_{5}k_{7}k_{9}}$ |
| EDD | $Vmax\left( f \right)= \frac{k_{1}k_{3}k_{5}[EDD]}{k_{1}k_{3}+k_{1}k_{4}+k_{1}k_{5}}$ |
|  | $Vmax\left( r \right)= \frac{k_{2}k_{4}[EDD]}{k_{2}+k_{3}+k_{4}}$ |
| EDA | $Vmax\left( f \right)= \frac{k_{1}k_{3}k_{5}k_{7}[EDA]}{k_{1}k_{3}k_{5}+k_{1}k_{3}k_{7}+k_{1}k_{4}k_{7}+k_{1}k_{5}k_{7}}$ |
|  | $Vmax\left( r \right)= \frac{k_{2}k_{4}k_{6}k_{8}[EDA]}{k_{2}k_{4}k_{7}+k_{2}k_{5}k_{7}+k_{3}k_{5}k_{7}}$ |
| PDH | $Vmax\left( f \right)= \frac{k_{1}k_{3}k_{5}k_{7}k_{9}[PDH]}{k_{1}k_{3}k_{5}k_{7}+k_{1}k_{3}k_{5}k_{9}+k_{1}k_{3}k_{7}k_{9}+k_{1}k_{4}k_{7}k_{9}+k_{1}k_{5}k_{7}k_{9}}$ |
|  | $Vmax\left( r \right)= \frac{k_{2}k_{4}k_{6}k_{8}k_{10}[PDH]}{k_{2}k_{4}k_{7}k_{9}+k_{2}k_{5}k_{7}k_{9}+k_{3}k_{5}k_{7}k_{9}}$ |
| CS | $Vmax\left( f \right)= \frac{k_{1}k_{3}k_{5}k_{7}[CS]}{k_{2}k_{4}k_{6}+k_{2}k_{4}k_{7}+k_{2}k_{5}k_{7}}$ |
|  | $Vmax\left( r \right)= \frac{k_{2}k_{4}k_{6}[CS]}{k_{2}k_{4}+k_{2}k_{5}+k_{2}k_{6}+k_{4}k_{6}}$ |
| ACONT | $Vmax\left( f \right)= \frac{k_{1}k_{3}k_{5}[ACONT]}{k_{1}k_{3}+k_{1}k_{4}+k_{1}k_{5}}$ |
|  | $Vmax\left( r \right)= \frac{k_{2}k_{4}[ACONT]}{k_{2}+k_{3}+k_{4}}$ |
| ICDHyr | $Vmax\left( f \right)= \frac{k_{1}k_{3}k_{5}k_{7}k_{9}[ICDHyr]}{k_{1}k_{3}k_{5}k_{7}+k_{1}k_{3}k_{5}k_{9}+k_{1}k_{3}k_{7}k_{9}+k_{1}k_{4}k_{7}k_{9}+k_{1}k_{5}k_{7}k_{9}}$ |
|  | $Vmax\left( r \right)= \frac{k_{2}k_{4}k_{6}k_{8}k_{10}[ICDHyr]}{k_{2}k_{4}k_{7}k_{9}+k_{2}k_{5}k_{7}k_{9}+k_{3}k_{5}k_{7}k_{9}}$ |
| AKGDH | $Vmax\left( f \right)= \frac{k_{1}k_{3}k_{5}k_{7}k_{9}[AKGDH]}{k_{1}k_{3}k_{5}k_{7}+k_{1}k_{3}k_{5}k_{9}+k_{1}k_{3}k_{7}k_{9}+k_{1}k_{4}k_{7}k_{9}+k_{1}k_{5}k_{7}k_{9}}$ |
|  | $Vmax\left( r \right)= \frac{k_{2}k_{4}k_{6}k_{8}k_{10}[AKGDH]}{k_{2}k_{4}k_{7}k_{9}+k_{2}k_{5}k_{7}k_{9}+k_{3}k_{5}k_{7}k_{9}}$ |
| SUCOAS | $Vmax\left( f \right)= \frac{k_{1}k_{3}k_{5}k_{7}[SUCOAS]}{k_{1}k_{3}k_{5}+k_{1}k_{3}k_{7}+k_{1}k_{4}k_{7}+k_{1}k_{5}k_{7}}$ |
|  | $Vmax\left( r \right)= \frac{k_{2}k_{4}k_{6}k_{8}[SUCOAS]}{k_{2}k_{4}k_{7}+k_{2}k_{5}k_{7}+k_{3}k_{5}k_{7}}$ |
| SUCDi | $Vmax\left( f \right)= \frac{k_{1}k_{3}k_{5}k_{7}[SUCDi]}{k_{1}k_{3}k_{5}+k_{1}k_{3}k_{7}+k_{1}k_{4}k_{7}+k_{1}k_{5}k_{7}}$ |
|  | $Vmax\left( r \right)= \frac{k_{2}k_{4}k_{6}k_{8}[SUCDi]}{k_{2}k_{4}k_{7}+k_{2}k_{5}k_{7}+k_{3}k_{5}k_{7}}$ |
| FUM | $Vmax\left( f \right)= \frac{k_{1}k_{3}k_{5}[FUM]}{k_{1}k_{3}+k_{1}k_{4}+k_{1}k_{5}}$ |
|  | $Vmax\left( r \right)= \frac{k_{2}k_{4}[FUM]}{k_{2}+k_{3}+k_{4}}$ |
| MDH | $Vmax\left( f \right)= \frac{k_{1}k_{3}k_{5}k_{7}[MDH]}{k_{1}k_{3}k_{5}+k_{1}k_{3}k_{7}+k_{1}k_{4}k_{7}+k_{1}k_{5}k_{7}}$ |
|  | $Vmax\left( r \right)= \frac{k_{2}k_{4}k_{6}k_{8}[MDH]}{k_{2}k_{4}k_{7}+k_{2}k_{5}k_{7}+k_{3}k_{5}k_{7}}$ |
| ICL | $Vmax\left( f \right)= \frac{k_{1}k_{3}k_{5}k_{7}[ICL]}{k_{1}k_{3}k_{5}+k_{1}k_{3}k_{7}+k_{1}k_{4}k_{7}+k_{1}k_{5}k_{7}}$ |
|  | $Vmax\left( r \right)= \frac{k_{2}k_{4}k_{6}k_{8}[ICL]}{k_{2}k_{4}k_{7}+k_{2}k_{5}k_{7}+k_{3}k_{5}k_{7}}$ |
| MALS | $Vmax\left( f \right)= \frac{k_{1}k_{3}k_{5}k_{7}[MALS]}{k_{2}k_{4}k_{6}+k_{2}k_{4}k_{7}+k_{2}k_{5}k_{7}}$ |
|  | $Vmax\left( r \right)= \frac{k_{2}k_{4}k_{6}[MALS]}{k_{2}k_{4}+k_{2}k_{5}+k_{2}k_{6}+k_{4}k_{6}}$ |
| ME2 | $Vmax\left( f \right)= \frac{k_{1}k_{3}k_{5}k_{7}k_{9}[ME2]}{k_{1}k_{3}k_{5}k_{7}+k_{1}k_{3}k_{5}k_{9}+k_{1}k_{3}k_{7}k_{9}+k_{1}k_{4}k_{7}k_{9}+k_{1}k_{5}k_{7}k_{9}}$ |
|  | $Vmax\left( r \right)= \frac{k_{2}k_{4}k_{6}k_{8}k_{10}[ME2]}{k_{2}k_{4}k_{7}k_{9}+k_{2}k_{5}k_{7}k_{9}+k_{3}k_{5}k_{7}k_{9}}$ |
| ME1 | $Vmax\left( f \right)= \frac{k_{1}k_{3}k_{5}k_{7}k_{9}[ME1]}{k_{1}k_{3}k_{5}k_{7}+k_{1}k_{3}k_{5}k_{9}+k_{1}k_{3}k_{7}k_{9}+k_{1}k_{4}k_{7}k_{9}+k_{1}k_{5}k_{7}k_{9}}$ |
|  | $Vmax\left( r \right)= \frac{k_{2}k_{4}k_{6}k_{8}k_{10}[ME1]}{k_{2}k_{4}k_{7}k_{9}+k_{2}k_{5}k_{7}k_{9}+k_{3}k_{5}k_{7}k_{9}}$ |
| PPC | $Vmax\left( f \right)= \frac{k_{1}k_{3}k_{5}k_{7}[PPC]}{k_{2}k_{4}k_{6}+k_{2}k_{4}k_{7}+k_{2}k_{5}k_{7}}$ |
|  | $Vmax\left( r \right)= \frac{k_{2}k_{4}k_{6}[PPC]}{k_{2}k_{4}+k_{2}k_{5}+k_{2}k_{6}+k_{4}k_{6}}$ |
| PPCK | $Vmax\left( f \right)= \frac{k_{1}k_{3}k_{5}k_{7}[PPCK]}{k_{2}k_{4}k_{6}+k_{2}k_{4}k_{7}+k_{2}k_{5}k_{7}}$ |
|  | $Vmax\left( r \right)= \frac{k_{2}k_{4}k_{6}k_{8}k_{10}[PPCK]}{k_{2}k_{4}k_{6}k_{9}+k_{2}k_{4}k_{7}k_{9}+k_{2}k_{5}k_{7}k_{9}}$ |
| PTAr/ACKr | $Vmax\left( f \right)= \frac{k_{1}k_{3}k_{5}k_{7}[PTAr-ACKr]}{k_{1}k_{3}k_{5}+k_{1}k_{3}k_{7}+k_{1}k_{4}k_{7}+k_{1}k_{5}k_{7}}$ |
|  | $Vmax\left( r \right)= \frac{k_{2}k_{4}k_{6}k_{8}[PTAr-ACKr]}{k_{2}k_{4}k_{7}+k_{2}k_{5}k_{7}+k_{3}k_{5}k_{7}}$ |
| GLUDy | $Vmax\left( f \right)= \frac{k_{1}k_{3}k_{5}k_{7}k_{9}[GLUDy]}{k_{2}k_{4}k_{6}k_{8}+k_{2}k_{4}k_{6}k_{9}+k_{2}k_{4}k_{7}k_{9}}$ |
|  | $Vmax\left( r \right)= \frac{k_{2}k_{4}k_{6}k_{8}[GLUDy]}{k_{2}k_{4}k_{6}+k_{2}k_{4}k_{7}+k_{2}k_{4}k_{8}+k_{2}k_{6}k_{8}+k_{4}k_{6}k_{8}}$ |
| GLNS | $Vmax\left( f \right)= \frac{k_{1}k_{3}k_{5}k_{7}k_{9}[GLNS]}{k_{2}k_{4}k_{6}k_{8}+k_{2}k_{4}k_{6}k_{9}+k_{2}k_{4}k_{7}k_{9}}$ |
|  | $Vmax\left( r \right)= \frac{k_{2}k_{4}k_{6}k_{8}[GLNS]}{k_{2}k_{4}k_{6}+k_{2}k_{4}k_{7}+k_{2}k_{4}k_{8}+k_{2}k_{6}k_{8}+k_{4}k_{6}k_{8}}$ |
| GHMT2r | $Vmax\left( f \right)= \frac{k_{1}k_{3}k_{5}k_{7}[GHMT2r]}{k_{1}k_{3}k_{5}+k_{1}k_{3}k_{7}+k_{1}k_{4}k_{7}+k_{1}k_{5}k_{7}}$ |
|  | $Vmax\left( r \right)= \frac{k_{2}k_{4}k_{6}k_{8}[GHMT2r]}{k_{2}k_{4}k_{7}+k_{2}k_{5}k_{7}+k_{3}k_{5}k_{7}}$ |
| GLYCL | $Vmax\left( f \right)= \frac{k_{1}k_{3}k_{5}k_{7}k_{9}k_{11}[GLYCL]}{\left[ \begin{aligned} k_{1}k_{3}k_{5}k_{7}k_{9}+k_{1}k_{3}k_{5}k_{7}k_{11}+k_{1}k_{3}k_{5}k_{9}k_{11}+\ldots\\ \ldots+k_{1}k_{3}k_{7}k_{9}k_{11}+k_{1}k_{4}k_{7}k_{9}k_{11}+k_{1}k_{5}k_{7}k_{9}k_{11} \end{aligned} \right]}$ |
|  | $Vmax\left( r \right)= \frac{k_{2}k_{4}k_{6}k_{8}k_{10}k_{12}[GLYCL]}{k_{2}k_{4}k_{7}k_{9}k_{11}+k_{2}k_{5}k_{7}k_{9}k_{11}+k_{3}k_{5}k_{7}k_{9}k_{11}}$ |
| SERD-L | $Vmax\left( f \right)= \frac{k_{1}k_{3}k_{5}k_{7}[SERD-L]}{k_{1}k_{3}k_{5}+k_{1}k_{3}k_{7}+k_{1}k_{4}k_{7}+k_{1}k_{5}k_{7}}$ |
|  | $Vmax\left( r \right)= \frac{k_{2}k_{4}k_{6}k_{8}[SERD-L]}{k_{2}k_{4}k_{7}+k_{2}k_{5}k_{7}+k_{3}k_{5}k_{7}}$ |
| MTHFR2 | $Vmax\left( f \right)= \frac{k_{1}k_{3}k_{5}k_{7}[MTHFR2]}{k_{2}k_{4}k_{6}+k_{2}k_{4}k_{7}+k_{2}k_{5}k_{7}}$ |
|  | $Vmax\left( r \right)= \frac{k_{2}k_{4}k_{6}[MTHFR2]}{k_{2}k_{4}+k_{2}k_{5}+k_{2}k_{6}+k_{4}k_{6}}$ |
| MTHFD | $Vmax\left( f \right)= \frac{k_{1}k_{3}k_{5}k_{7}[MTHFD]}{k_{1}k_{3}k_{5}+k_{1}k_{3}k_{7}+k_{1}k_{4}k_{7}+k_{1}k_{5}k_{7}}$ |
|  | $Vmax\left( r \right)= \frac{k_{2}k_{4}k_{6}k_{8}[MTHFD]}{k_{2}k_{4}k_{7}+k_{2}k_{5}k_{7}+k_{3}k_{5}k_{7}}$ |
| NADTRHD | $Vmax\left( f \right)= \frac{k_{1}k_{3}k_{5}[NADTRHD]}{k_{1}k_{3}+k_{1}k_{4}+k_{1}k_{5}}$ |
|  | $Vmax\left( r \right)= \frac{k_{2}k_{4}[NADTRHD]}{k_{2}+k_{3}+k_{4}}$ |
