# Supplementary material for: From Escherichia coli mutant 13C labeling data to a core kinetic model: A kinetic model parameterization pipeline
Source: PLoS Comput Biol. 2019 Sep 10;15(9):e1007319. doi: 10.1371/journal.pcbi.1007319 (PMC6759195; doi:10.1371/journal.pcbi.1007319)
Supplement: S7 File — (DOCX) [file pcbi.1007319.s007.docx]

**S7 File: Michaelis-Menten rate expressions for central carbon metabolism reactions in k-ecoli74**

| **PGI** |
| --- |
| $\frac{Vmax\left( f \right)*\left[ g6p \right]-Vmax\left( r \right)*[f6p]}{\left[ 1+\frac{\left[ g6p \right]}{K_{m}\left( g6p \right)}+\frac{\left[ f6p \right]}{K_{m}\left( f6p \right)}+kI\left( pep \right)*\left[ pep \right]+kI\left( 6pg \right)*\left[ 6pg \right]+\frac{\left[ f6p \right]*[6pg]}{K_{m}\left( f6p,6pg \right)}+\frac{\left[ g6p \right]*[6pg]}{K_{m}\left( g6p,6pg \right)} \right]}$ |
| **PFK** |
| $\frac{Vmax\left( f \right)*\left[ f6p \right]*[atp]-Vmax\left( r \right)*[fdp]}{\left[ \begin{aligned} 1+\frac{\left[ f6p \right]}{K_{m}\left( f6p \right)}+\frac{\left[ atp \right]}{K_{m}\left( atp \right)}+\frac{\left[ fdp \right]}{K_{m}\left( fdp \right)}+kI\left( atp \right)*\left[ atp \right]+kI\left( pep \right)*\left[ pep \right]+kI\left( f6p \right)*\left[ f6p \right]+\ldots\\ \ldots+\frac{\left[ f6p \right]*\left[ atp \right]}{K_{m}\left( f6p,6pg \right)}+\frac{\left[ f6p \right]*\left[ pep \right]}{K_{m}\left( f6p,pep \right)}+\frac{\left[ f6p \right]^{2}}{K_{m}\left( f6p,f6p \right)}+\frac{\left[ atp \right]*\left[ fdp \right]}{K_{m}\left( atp,fdp \right)}+\frac{\left[ atp \right]^{2}}{K_{m}\left( atp,atp \right)}+\ldots\\ \ldots+\frac{\left[ atp \right]*\left[ pep \right]}{K_{m}\left( atp,pep \right)}+\frac{\left[ atp \right]*\left[ f6p \right]}{K_{m}\left( atp,f6p \right)}+\frac{\left[ fdp \right]*\left[ pep \right]}{K_{m}\left( fdp,pep \right)}+\frac{\left[ fdp \right]*\left[ f6p \right]}{K_{m}\left( fdp,f6p \right)} \end{aligned} \right]}$ |
| **FBP** |
| $\frac{Vmax\left( f \right)*\left[ fdp \right]-Vmax\left( r \right)*[f6p]}{\left[ \begin{aligned} 1+\frac{\left[ fdp \right]}{K_{m}\left( fdp \right)}+\frac{\left[ f6p \right]}{K_{m}\left( f6p \right)}+kI\left( fdp \right)*\left[ fdp \right]+kI\left( pep \right)*\left[ pep \right]+kI\left( g6p \right)*\left[ g6p \right]+\ldots\\ \ldots+\frac{\left[ f6p \right]*\left[ g6p \right]}{K_{m}\left( f6p,g6p \right)}+\frac{\left[ f6p \right]*\left[ pep \right]}{K_{m}\left( f6p,pep \right)}+\frac{\left[ fdp \right]*\left[ g6p \right]}{K_{m}\left( fdp,g6p \right)}+\frac{\left[ fdp \right]*\left[ pep \right]}{K_{m}\left( fdp,pep \right)} \end{aligned} \right]}$ |
| **FBA** |
| $\frac{Vmax\left( f \right)*\left[ fdp \right]-Vmax\left( r \right)*\left[ g3p \right]*[dhap]}{\left[ \begin{aligned} 1+\frac{\left[ fdp \right]}{K_{m}\left( fdp \right)}+\frac{\left[ g3p \right]}{K_{m}\left( g3p \right)}+\frac{\left[ dhap \right]}{K_{m}\left( dhap \right)}+kI\left( 3gp \right)*\left[ 3gp \right]+kI\left( cys \right)*\left[ cys \right]+kI\left( dhap \right)*\left[ dhap \right]+\ldots\\ \ldots+kI\left( g3p \right)*\left[ g3p \right]+\frac{\left[ fdp \right]*\left[ dhap \right]}{K_{m}^{1}\left( fdp,dhap \right)}+\frac{\left[ fdp \right]*\left[ dhap \right]}{+K_{m}^{2}\left( fdp,dhap \right)}+\frac{\left[ fdp \right]*\left[ g3p \right]}{K_{m}\left( fdp,g3p \right)}+\frac{\left[ g3p \right]*\left[ dhap \right]}{K_{m}\left( g3p,dhap \right)}+\ldots\\ \ldots+\frac{\left[ dhap \right]*\left[ 3pg \right]}{K_{m}\left( dhap,3pg \right)}+\frac{\left[ dhap \right]*\left[ cys \right]}{K_{m}\left( dhap,cys \right)}+\frac{\left[ dhap \right]^{2}}{K_{m}\left( dhap,dhap \right)}+\frac{\left[ dhap \right]*\left[ g3p \right]}{K_{m}\left( dhap,g3p \right)}+\ldots\\ \ldots+\frac{\left[ fdp \right]*\left[ dhap \right]^{2}}{K_{m}\left( fdp,dhap,dhap \right)}+\frac{\left[ fdp \right]*\left[ dhap \right]*\left[ g3p \right]}{K_{m}\left( fdp,dhap,g3p \right)}+\frac{\left[ g3p \right]^{2}*\left[ dhap \right]}{K_{m}\left( g3p,dhap,g3p \right)}+\frac{\left[ g3p \right]*\left[ dhap \right]^{2}}{K_{m}\left( g3p,dhap,dhap \right)} \end{aligned} \right]}$ |
| **TPI** |
| $\frac{Vmax\left( f \right)*\left[ dhap \right]-Vmax\left( r \right)*[g3p]}{\left[ 1+\frac{\left[ dhap \right]}{K_{m}\left( dhap \right)}+\frac{\left[ g3p \right]}{K_{m}\left( g3p \right)} \right]}$ |
| **GAPD/PGK** |
| $\frac{Vmax\left( f \right)*\left[ g3p \right]-Vmax\left( r \right)*[atp]*[3pg]*[nadh]}{\left[ \begin{aligned} 1+\frac{\left[ g3p \right]}{K_{m}\left( g3p \right)}+\frac{\left[ nadh \right]}{K_{m}\left( nadh \right)} +\frac{\left[ atp \right]}{K_{m}\left( atp \right)}+kI\left( atp \right)*\left[ atp \right]+\frac{\left[ atp \right]^{2}}{K_{m}\left( atp,atp \right)}+\frac{\left[ 3pg \right]*\left[ atp \right]}{K_{m}\left( 3pg,atp \right)}+\ldots\\ \ldots+\frac{\left[ g3p \right]*\left[ atp \right]}{K_{m}^{1}\left( g3p,atp \right)}+\frac{\left[ g3p \right]*\left[ atp \right]}{K_{m}^{2}\left( g3p,atp \right)}+\frac{\left[ 3pg \right]*\left[ atp \right]^{2}}{K_{m}\left( 3pg,atp,atp \right)}+\frac{\left[ 3pg \right]*\left[ nadh \right]*\left[ atp \right]}{K_{m}\left( 3pg,nadh,atp \right)}+\ldots\\ \ldots++\frac{\left[ g3p \right]*\left[ atp \right]^{2}}{K_{m}\left( g3p,atp,atp \right)}++\frac{\left[ 3pg \right]*\left[ nadh \right]*\left[ atp \right]}{K_{m}\left( 3pg,nadh,atp \right)}++\frac{\left[ g3p \right]*\left[ atp \right]^{2}}{K_{m}\left( g3p,atp,atp \right)}+\ldots\\ \ldots+\frac{\left[ g3p \right]*\left[ 3pg \right]*\left[ atp \right]}{K_{m}\left( g3p,3pg,atp \right)}+\frac{\left[ g3p \right]*\left[ 3pg \right]*\left[ atp \right]^{2}}{K_{m}\left( g3p,3pg,atp,atp \right)}+\frac{\left[ nadh \right]*\left[ 3pg \right]*\left[ atp \right]^{2}}{K_{m}\left( g3p,3pg,atp,atp \right)} \end{aligned} \right]}$ |
| **PGM/ENO** |
| $\frac{Vmax\left( f \right)*\left[ 3pg \right]-Vmax\left( r \right)*[pep]}{\left[ 1+\frac{\left[ 3pg \right]}{K_{m}\left( 3pg \right)}+\frac{\left[ pep \right]}{K_{m}\left( pep \right)} \right]}$ |
| **PYK** |
| $\frac{Vmax\left( f \right)*\left[ pep \right]-Vmax\left( r \right)*[pyr]*[atp]}{\left[ \begin{aligned} 1+\frac{\left[ pep \right]}{K_{m}\left( pep \right)}+\frac{\left[ pyr \right]}{K_{m}\left( pyr \right)}+\frac{\left[ atp \right]}{K_{m}\left( atp \right)} +kI\left( atp \right)*\left[ atp \right]+kI\left( succoa \right)*\left[ succoa \right]+\ldots\\ \ldots+\frac{\left[ pyr \right]*\left[ succoa \right]}{K_{m}\left( pyr,succoa \right)}+\frac{\left[ pyr \right]*\left[ atp \right]}{K_{m}^{1}\left( pyr,atp \right)}+\frac{\left[ pyr \right]*\left[ atp \right]}{{K_{m}}_{m}^{2}\left( pyr,atp \right)}+\frac{\left[ pep \right]*\left[ succoa \right]}{K_{m}\left( pep,succoa \right)}+\ldots\\ \ldots+\frac{\left[ pep \right]*\left[ atp \right]}{K_{m}^{1}\left( pep,atp \right)}+\frac{\left[ pep \right]*\left[ atp \right]}{{K_{m}}_{m}^{2}\left( pep,atp \right)}+\frac{\left[ pyr \right]*\left[ atp \right]*\left[ succoa \right]}{K_{m}\left( pyr,atp,succoa \right)}+\frac{\left[ pyr \right]*\left[ atp \right]^{2}}{K_{m}^{1}\left( pyr,atp,atp \right)}+\ldots\\ \ldots+\frac{\left[ pyr \right]*\left[ atp \right]^{2}}{K_{m}^{2}\left( pyr,atp,atp \right)}+\frac{\left[ pep \right]*\left[ atp \right]^{2}}{K_{m}\left( pep,atp,atp \right)} \end{aligned} \right]}$ |
| **G6PDH2r** |
| $\frac{Vmax\left( f \right)*\left[ g6p \right]-Vmax\left( r \right)*\left[ 6pg \right]*[nadph]}{\left[ \begin{aligned} 1+\frac{\left[ 6pg \right]}{K_{m}\left( 6pg \right)}+\frac{\left[ co2 \right]}{K_{m}\left( co2 \right)}+\frac{\left[ nadph \right]}{K_{m}\left( nadph \right)}+kI\left( atp \right)*\left[ atp \right]+kI\left( nadph \right)*\left[ nadph \right]+\ldots\\ \ldots+kI\left( fdp \right)*\left[ fdp \right]+kI\left( ru5p \right)*\left[ ru5p \right]+\frac{\left[ 6pg \right]*\left[ ru5p \right]}{K_{m}^{1}\left( 6pg,ru5p \right)}+\frac{\left[ 6pg \right]*\left[ nadph \right]}{K_{m}\left( 6pg,nadph \right)}+\ldots\\ \ldots+\frac{\left[ 6pg \right]*\left[ fdp \right]}{K_{m}\left( 6pg,fdp \right)}+\frac{\left[ 6pg \right]*\left[ ru5p \right]}{K_{m}\left( 6pg,ru5p \right)}+\frac{\left[ co2 \right]*\left[ ru5p \right]}{K_{m}\left( co2,ru5p \right)}+\frac{\left[ co2 \right]*\left[ nadph \right]}{K_{m}\left( co2,nadph \right)}+\ldots\\ \ldots+\frac{\left[ ru5p \right]*\left[ nadph \right]}{K_{m}\left( ru5p,nadph \right)}+\frac{\left[ nadph \right]*\left[ atp \right]}{K_{m}^{1}\left( nadph,atp \right)}+\frac{\left[ nadph \right]*\left[ atp \right]}{K_{m}^{2}\left( nadph,atp \right)}+\frac{\left[ nadph \right]^{2}}{K_{m}\left( nadph,nadph \right)}+\ldots\\ \ldots+\frac{\left[ nadph \right]*\left[ fdp \right]}{K_{m}^{2}\left( nadph,fdp \right)}+\frac{\left[ nadph \right]*\left[ ru5p \right]}{K_{m}^{2}\left( nadph,ru5p \right)}+\frac{\left[ 6pg \right]*\left[ ru5p \right]*\left[ nadph \right]}{K_{m}\left( 6pg,ru5p,nadph \right)}+\ldots\\ \ldots+\frac{\left[ 6pg \right]*\left[ nadph \right]*\left[ fdp \right]}{K_{m}\left( 6pg,ru5p,nadph \right)}+\frac{\left[ 6pg \right]*\left[ nadph \right]*\left[ ru5p \right]}{K_{m}\left( 6pg,nadph,ru5p \right)}+\frac{\left[ co2 \right]*\left[ nadph \right]*\left[ ru5p \right]}{K_{m}\left( co2,nadph,ru5p \right)}+\ldots\\ \ldots+\frac{\left[ ru5p \right]^{2}*\left[ nadph \right]}{K_{m}\left( ru5p,nadph,ru5p \right)}+\frac{\left[ ru5p \right]*\left[ nadph \right]*\left[ fdp \right]}{K_{m}\left( ru5p,nadph,fdp \right)}+\frac{\left[ ru5p \right]*\left[ nadph \right]^{2}}{K_{m}\left( ru5p,nadph,nadph \right)}+\ldots\\ \ldots+\frac{\left[ ru5p \right]*\left[ nadph \right]*\left[ atp \right]}{K_{m}\left( ru5p,nadph,atp \right)}+\frac{\left[ co2 \right]*\left[ ru5p \right]^{2}*\left[ nadph \right]}{K_{m}\left( co2,ru5p,nadph,ru5p \right)}+\frac{\left[ co2 \right]*\left[ ru5p \right]*\left[ nadph \right]*\left[ fdp \right]}{K_{m}\left( co2,ru5p,nadph,fdp \right)}+\ldots\\ \ldots+\frac{\left[ 6pg \right]*\left[ ru5p \right]^{2}*\left[ nadph \right]}{K_{m}\left( 6pg,ru5p,nadph,ru5p \right)}+\frac{\left[ 6pg \right]*\left[ ru5p \right]*\left[ nadph \right]*\left[ fdp \right]}{K_{m}\left( 6pg,ru5p,nadph,fdp \right)} \end{aligned} \right]}$ |
| **RPE** |
| $\frac{Vmax\left( f \right)*\left[ ru5p \right]-Vmax\left( r \right)*[x5p]}{\left[ 1+\frac{\left[ ru5p \right]}{K_{m}\left( ru5p \right)}+\frac{\left[ x5p \right]}{K_{m}\left( x5p \right)} \right]}$ |
| **RPI** |
| $\frac{Vmax\left( f \right)*\left[ ru5p \right]-Vmax\left( r \right)*[r5p]}{\left[ 1+\frac{\left[ ru5p \right]}{K_{m}\left( ru5p \right)}+\frac{\left[ r5p \right]}{K_{m}\left( r5p \right)}+kI\left( 6pg \right)*\left[ 6pg \right]+kI\left( f6p \right)*\left[ f6p \right] \right]}$ |
| **TKThlf1** |
| $\frac{Vmax\left( f \right)*\left[ x5p \right]-Vmax\left( r \right)*[ec2]*[g3p]}{\left[ 1+\frac{\left[ x5p \right]}{K_{m}\left( x5p \right)}+\frac{\left[ g3p \right]}{K_{m}\left( g3p \right)}+\frac{\left[ ec2 \right]}{K_{m}\left( ec2 \right)}+\frac{\left[ x5p \right]*\left[ ec2 \right]}{K_{m}\left( x5p,ec2 \right)}+\frac{\left[ g3p \right]*\left[ ec2 \right]}{K_{m}\left( g3p,ec2 \right)} \right]}$ |
| **TKThlf2** |
| $\frac{Vmax\left( f \right)*\left[ s7p \right]-Vmax\left( r \right)*[ec2]*[r5p]}{\left[ 1+\frac{\left[ s7p \right]}{K_{m}\left( s7p \right)}+\frac{\left[ r5p \right]}{K_{m}\left( r5p \right)}+\frac{\left[ ec2 \right]}{K_{m}\left( ec2 \right)}+\frac{\left[ s7p \right]*\left[ ec2 \right]}{K_{m}\left( s7p,ec2 \right)}+\frac{\left[ r5p \right]*\left[ ec2 \right]}{K_{m}\left( r5p,ec2 \right)} \right]}$ |
| **TKThlf3** |
| $\frac{Vmax\left( f \right)*\left[ f6p \right]-Vmax\left( r \right)*[ec2]*[e4p]}{\left[ 1+\frac{\left[ f6p \right]}{K_{m}\left( f6p \right)}+\frac{\left[ e4p \right]}{K_{m}\left( e4p \right)}+\frac{\left[ ec2 \right]}{K_{m}\left( ec2 \right)}+\frac{\left[ f6p \right]*\left[ ec2 \right]}{K_{m}\left( f6p,ec2 \right)}+\frac{\left[ e4p \right]*\left[ ec2 \right]}{K_{m}\left( e4p,ec2 \right)} \right]}$ |
| **TALA** |
| $\frac{Vmax\left( f \right)*\left[ g3p \right]*\left[ s7p \right]-Vmax\left( r \right)*[e4p]*[f6p]}{\left[ \begin{aligned} 1+\frac{\left[ g3p \right]}{K_{m}\left( g3p \right)}+\frac{\left[ s7p \right]}{K_{m}\left( s7p \right)}+\frac{\left[ e4p \right]}{K_{m}\left( e4p \right)}+\frac{\left[ f6p \right]}{K_{m}\left( f6p \right)}+kI\left( so4 \right)*\left[ so4 \right]+\frac{\left[ e4p \right]*\left[ f6p \right]}{K_{m}\left( e4p,f6p \right)}+\ldots\\ \ldots+\frac{\left[ s7p \right]*\left[ so4 \right]}{K_{m}\left( s7p,so4 \right)}+ \frac{\left[ s7p \right]*\left[ e4p \right]}{K_{m}\left( s7p,e4p \right)}+ \frac{\left[ g3p \right]*\left[ f6p \right]}{K_{m}\left( g3p,f6p \right)}+ \frac{\left[ g3p \right]*\left[ s7p \right]}{K_{m}\left( g3p,s7p \right)}+ \frac{\left[ s7p \right]*\left[ e4p \right]*\left[ f6p \right]}{K_{m}\left( s7p,e4p,f6p \right)}+\ldots\\ \ldots+ \frac{\left[ g3p \right]*\left[ s7p \right]*\left[ f6p \right]}{K_{m}\left( g3p,s7p,f6p \right)} \end{aligned} \right]}$ |
| **EDD** |
| $\frac{Vmax\left( f \right)*\left[ 6pg \right]-Vmax\left( r \right)*[kdpg]}{\left[ 1+\frac{\left[ 6pg \right]}{K_{m}\left( 6pg \right)}+\frac{\left[ kdpg \right]}{K_{m}\left( kdpg \right)}+kI\left( o2 \right)*\left[ o2 \right] \right]}$ |
| **EDA** |
| $\frac{Vmax\left( f \right)*\left[ kdpg \right]-Vmax\left( r \right)*[g3p]*[pyr]}{\left[ \begin{aligned} 1+\frac{\left[ kdpg \right]}{K_{m}\left( kdpg \right)}+\frac{\left[ pyr \right]}{K_{m}\left( pyr \right)}+\frac{\left[ g3p \right]}{K_{m}\left( g3p \right)}+kI\left( 6pg \right)*\left[ 6pg \right]+kI\left( g3p \right)*\left[ g3p \right]+ \frac{\left[ g3p \right]^{2}}{K_{m}\left( g3p,f6p \right)}+\ldots\\ \ldots+ \frac{\left[ g3p \right]*\left[ 6pg \right]}{K_{m}\left( g3p,6pg \right)} + \frac{\left[ pyr \right]*\left[ g3p \right]}{K_{m}\left( pyr,g3p \right)}+ \frac{\left[ kdpg \right]*\left[ g3p \right]}{K_{m}^{1}\left( kdpg,g3p \right)} + \frac{\left[ kdpg \right]*\left[ g3p \right]}{K_{m}^{2}\left( kdpg,g3p \right)} + \frac{\left[ pyr \right]*\left[ g3p \right]^{2}}{K_{m}\left( pyr,g3p,g3p \right)}+\ldots\\ \ldots+\frac{\left[ kdpg \right]*\left[ g3p \right]^{2}}{K_{m}\left( kdpg,g3p,g3p \right)} \end{aligned} \right]}$ |
| **PDH** |
| $\frac{Vmax\left( f \right)*\left[ pyr \right]-Vmax\left( r \right)*[nadh]*[co2]*[accoa]}{\left[ \begin{aligned} 1+\frac{\left[ pyr \right]}{K_{m}\left( pyr \right)}+\frac{\left[ accoa \right]}{K_{m}\left( accoa \right)}+\frac{\left[ nadh \right]}{K_{m}\left( nadh \right)}+ \frac{\left[ co2 \right]*[nadh]}{K_{m}\left( co2,nadh \right)}+ \frac{\left[ accoa \right]*[nadh]}{K_{m}\left( accoa,nadh \right)}+\ldots\\ \ldots+ \frac{\left[ accoa \right]*\left[ co2 \right]}{K_{m}\left( accoa,co2 \right)} + \frac{\left[ pyr \right]*\left[ nadh \right]}{K_{m}\left( pyr,nadh \right)}+ \frac{\left[ pyr \right]*\left[ co2 \right]}{K_{m}\left( pyr,co2 \right)} + \frac{\left[ pyr \right]*\left[ co2 \right]*[nadh]}{K_{m}\left( pyr,co2,nadh \right)}+\ldots\\ \ldots+\frac{\left[ accoa \right]*\left[ co2 \right]*[nadh]}{K_{m}\left( accoa,co2,nadh \right)} \end{aligned} \right]}$ |
| **CS** |
| $\frac{Vmax\left( f \right)*\left[ accoa \right]*[oac]-Vmax\left( r \right)*[cit]}{\left[ \begin{aligned} 1+\frac{\left[ accoa \right]}{K_{m}\left( accoa \right)}+\frac{\left[ oac \right]}{K_{m}\left( oac \right)}+\frac{\left[ cit \right]}{K_{m}\left( cit \right)}+ \frac{\left[ accoa \right]*[oac]}{K_{m}\left( accoa,oac \right)}+ \frac{\left[ oac \right]*[cit]}{K_{m}\left( oac,cit \right)} \end{aligned} \right]}$ |
| **ACONT** |
| $\frac{Vmax\left( f \right)*\left[ cit \right]-Vmax\left( r \right)*[icit]}{\left[ \begin{aligned} 1+\frac{\left[ cit \right]}{K_{m}\left( cit \right)}+\frac{\left[ icit \right]}{K_{m}\left( icit \right)} \end{aligned} \right]}$ |
| **ICDHyr** |
| $\frac{Vmax\left( f \right)*\left[ icit \right]-Vmax\left( r \right)*[nadph]*[co2]*[akg]}{\left[ \begin{aligned} 1+\frac{\left[ icit \right]}{K_{m}\left( icit \right)}+\frac{\left[ akg \right]}{K_{m}(akg)}+\frac{\left[ nadph \right]}{K_{m}\left( nadph \right)}+kI\left( glx \right)*\left[ glx \right]+kI\left( oac \right)*\left[ oac \right]+kI\left( pep \right)*\left[ pep \right]+\ldots\\ \ldots+ \frac{\left[ icit \right]*\left[ co2 \right]}{K_{m}\left( icit,co2 \right)}+ \frac{\left[ icit \right]*\left[ nadph \right]}{K_{m}\left( icit,nadph \right)}+ \frac{\left[ icit \right]*\left[ pep \right]}{K_{m}\left( icit,pep \right)}+ \frac{\left[ akg \right]*\left[ co2 \right]}{K_{m}\left( akg,co2 \right)}+ \frac{\left[ akg \right]*\left[ nadph \right]}{K_{m}\left( akg,nadph \right)}+\ldots\\ \ldots+ \frac{\left[ co2 \right]*\left[ nadph \right]}{K_{m}\left( co2,nadph \right)}+ \frac{\left[ nadph \right]*\left[ glx \right]}{K_{m}\left( nadph,glx \right)}+ \frac{\left[ nadph \right]*\left[ oac \right]}{K_{m}\left( nadph,oac \right)}+ \frac{\left[ nadph \right]*\left[ pep \right]}{K_{m}\left( nadph,pep \right)}+\ldots\\ \ldots+ \frac{\left[ icit \right]*\left[ co2 \right]*\left[ nadph \right]}{K_{m}\left( icit,co2,nadph \right)}+ \frac{\left[ icit \right]*\left[ nadph \right]*\left[ pep \right]}{K_{m}\left( icit,nadph,pep \right)}+ \frac{\left[ akg \right]*\left[ co2 \right]*\left[ nadph \right]}{K_{m}\left( akg,co2,nadph \right)}+\ldots\\ \ldots+ \frac{\left[ co2 \right]*\left[ nadph \right]*\left[ pep \right]}{K_{m}\left( co2,nadph,pep \right)}+ \frac{\left[ co2 \right]*\left[ nadph \right]*\left[ oac \right]}{K_{m}\left( co2,nadph,oac \right)}+ \frac{\left[ co2 \right]*\left[ nadph \right]*\left[ glx \right]}{K_{m}\left( co2,nadph,glx \right)}+\ldots\\ \ldots+ \frac{\left[ akg \right]*\left[ co2 \right]*\left[ nadph \right]*\left[ pep \right]}{K_{m}\left( akg,co2,nadph,pep \right)}+ \frac{\left[ icit \right]*\left[ co2 \right]*\left[ nadph \right]*\left[ pep \right]}{K_{m}\left( icit,co2,nadph,pep \right)} \end{aligned} \right]}$ |
| **AKGDH** |
| $\frac{Vmax\left( f \right)*\left[ akg \right]-Vmax\left( r \right)*[nadh]*[co2]*[succoa]}{\left[ \begin{aligned} 1+\frac{\left[ akg \right]}{K_{m}akg}+\frac{\left[ succoa \right]}{K_{m}\left( succoa \right)}+\frac{\left[ nadh \right]}{K_{m}\left( nadh \right)}+ \frac{\left[ co2 \right]*\left[ nadh \right]}{K_{m}\left( co2,nadh \right)}+ \frac{\left[ succoa \right]*\left[ nadh \right]}{K_{m}\left( succoa,nadh \right)}+\ldots\\ \ldots+ \frac{\left[ succoa \right]*\left[ co2 \right]}{K_{m}\left( succoa,co2 \right)}+ \frac{\left[ akg \right]*\left[ nadh \right]}{K_{m}\left( akg,nadh \right)}+ \frac{\left[ akg \right]*\left[ co2 \right]}{K_{m}\left( akg,co2 \right)}+ \frac{\left[ akg \right]*\left[ co2 \right]*\left[ nadh \right]}{K_{m}\left( akg,co2,nadh \right)}+\ldots\\ \ldots+ \frac{\left[ succoa \right]*\left[ co2 \right]*\left[ nadh \right]}{K_{m}\left( succoa,co2,nadh \right)} \end{aligned} \right]}$ |
| **SUCOAS** |
| $\frac{Vmax\left( f \right)*\left[ succoa \right]-Vmax\left( r \right)*[atp]*[suc]}{\left[ \begin{aligned} 1+\frac{\left[ succoa \right]}{K_{m}\left( succoa \right)}+\frac{\left[ suc \right]}{K_{m}(akg)}+\frac{\left[ atp \right]}{K_{m}\left( atp \right)}+kI\left( atp \right)*\left[ atp \right]+kI\left( nadh \right)*\left[ nadh \right]+\ldots\\ \ldots+kI\left( akg \right)*\left[ akg \right]+kI\left( suc \right)*\left[ suc \right] \frac{\left[ succoa \right]*\left[ atp \right]}{K_{m}\left( succoa,atp \right)}+ \frac{\left[ succoa \right]*\left[ suc \right]}{K_{m}\left( succoa,suc \right)}+ \frac{\left[ suc \right]*\left[ atp \right]}{K_{m}\left( suc,atp \right)}+\ldots\\ \ldots+ \frac{\left[ atp \right]^{2}}{K_{m}\left( atp,atp \right)}+ \frac{\left[ atp \right]*\left[ nadh \right]}{K_{m}\left( atp,nadh \right)}+\frac{\left[ atp \right]*\left[ akg \right]}{K_{m}\left( atp,akg \right)}+\frac{\left[ atp \right]*\left[ suc \right]}{K_{m}\left( atp,suc \right)}+\ldots\\ \ldots+ \frac{\left[ succoa \right]*\left[ atp \right]*\left[ suc \right]}{K_{m}\left( succoa,atp,suc \right)}+ \frac{\left[ suc \right]^{2}*\left[ atp \right]}{K_{m}\left( suc,atp,suc \right)} \end{aligned} \right]}$ |
| **SUCDi** |
| $\frac{Vmax\left( f \right)*\left[ x5p \right]-Vmax\left( r \right)*[ec2]*[g3p]}{\left[ 1+\frac{\left[ suc \right]}{K_{m}\left( suc \right)}+\frac{\left[ fadh2 \right]}{K_{m}\left( fadh2 \right)}+\frac{\left[ fum \right]}{K_{m}\left( fum \right)}+\frac{\left[ suc \right]*\left[ fum \right]}{K_{m}\left( suc,fum \right)}+\frac{\left[ fadh2 \right]*\left[ fum \right]}{K_{m}\left( fadh2,fum \right)} \right]}$ |
| **MDH** |
| $\frac{Vmax\left( f \right)*\left[ mal \right]-Vmax\left( r \right)*[nadh]*[oac]}{\left[ 1+\frac{\left[ mal \right]}{K_{m}\left( mal \right)}+\frac{\left[ oac \right]}{K_{m}\left( oac \right)}+\frac{\left[ nadh \right]}{K_{m}\left( nadh \right)}+\frac{\left[ mal \right]*\left[ nadh \right]}{K_{m}\left( mal,nadh \right)}+\frac{\left[ oac \right]*\left[ nadh \right]}{K_{m}\left( oac,nadh \right)} \right]}$ |
| **ICL** |
| $\frac{Vmax\left( f \right)*\left[ icit \right]-Vmax\left( r \right)*[glx]*[suc]}{\left[ \begin{aligned} 1+\frac{\left[ icit \right]}{K_{m}\left( icit \right)}+\frac{\left[ suc \right]}{K_{m}\left( suc \right)}+\frac{\left[ glx \right]}{K_{m}\left( glx \right)}+kI\left( glx \right)*\left[ glx \right]+kI\left( 3pg \right)*\left[ 3pg \right]+kI\left( so4 \right)*\left[ so4 \right]+\ldots\\ \ldots+kI\left( akg \right)*\left[ akg \right]+kI\left( suc \right)*\left[ suc \right]+\frac{\left[ icit \right]*\left[ glx \right]}{K_{m}\left( icit,glx \right)}+\frac{\left[ icit \right]*\left[ pep \right]}{K_{m}\left( icit,pep \right)} +\frac{\left[ icit \right]*\left[ akg \right]}{K_{m}\left( icit,akg \right)}+\ldots\\ \ldots+\frac{\left[ icit \right]*\left[ suc \right]}{K_{m}\left( icit,suc \right)} +\frac{\left[ suc \right]*\left[ glx \right]}{K_{m}\left( suc,glx \right)} +\frac{\left[ glx \right]^{2}}{K_{m}\left( glx,glx \right)} +\frac{\left[ glx \right]*\left[ 3pg \right]}{K_{m}\left( glx,3pg \right)}+\frac{\left[ glx \right]*\left[ so4 \right]}{K_{m}\left( glx,so4 \right)}+\ldots\\ \ldots+\frac{\left[ glx \right]*\left[ akg \right]}{K_{m}\left( glx,akg \right)}+\frac{\left[ glx \right]*\left[ suc \right]}{K_{m}\left( glx,suc \right)}+\frac{\left[ icit \right]*\left[ glx \right]*\left[ pep \right]}{K_{m}\left( icit,glx,pep \right)}+\frac{\left[ icit \right]*\left[ glx \right]*\left[ akg \right]}{K_{m}\left( icit,glx,akg \right)}+\ldots\\ \ldots+\frac{\left[ icit \right]*\left[ glx \right]*\left[ suc \right]}{K_{m}\left( icit,glx,suc \right)}+\frac{\left[ suc \right]*\left[ glx \right]*\left[ pep \right]}{K_{m}\left( suc,glx,pep \right)}+\frac{\left[ suc \right]*\left[ glx \right]*\left[ akg \right]}{K_{m}\left( suc,glx,akg \right)}+\frac{\left[ suc \right]^{2}*\left[ glx \right]}{K_{m}\left( suc,glx,suc \right)} \end{aligned} \right]}$ |
| **MALS** |
| $\frac{Vmax\left( f \right)*\left[ glx \right]*[accoa]-Vmax\left( r \right)*[mal]}{\left[ 1+\frac{\left[ glx \right]}{K_{m}\left( glx \right)}+\frac{\left[ accoa \right]}{K_{m}\left( accoa \right)}+\frac{\left[ mal \right]}{K_{m}\left( mal \right)}+\frac{\left[ glx \right]*\left[ accoa \right]}{K_{m}\left( glx,accoa \right)}+\frac{\left[ accoa \right]*\left[ mal \right]}{K_{m}\left( accoa,mal \right)} \right]}$ |
| **ME2** |
| $\frac{Vmax\left( f \right)*\left[ mal \right]-Vmax\left( r \right)*[pyr]*[nadph]*[co2]}{\left[ \begin{aligned} 1+\frac{\left[ mal \right]}{K_{m}\left( mal \right)}+\frac{\left[ co2 \right]}{K_{m}\left( co2 \right)}+\frac{\left[ pyr \right]}{K_{m}\left( pyr \right)}+kI\left( atp \right)*\left[ atp \right]+kI\left( accoa \right)*\left[ accoa \right]+\frac{\left[ mal \right]*\left[ nadph \right]}{K_{m}\left( mal,nadph \right)}+\ldots\\ \ldots+\frac{\left[ mal \right]*\left[ pyr \right]}{K_{m}\left( mal,pyr \right)}+\frac{\left[ mal \right]*\left[ atp \right]}{K_{m}\left( mal,atp \right)} +\frac{\left[ mal \right]*\left[ accoa \right]}{K_{m}\left( mal,accoa \right)} +\frac{\left[ co2 \right]*\left[ nadph \right]}{K_{m}\left( co2,nadph \right)}+\frac{\left[ co2 \right]*\left[ pyr \right]}{K_{m}\left( co2,pyr \right)}+\ldots\\ \ldots+\frac{\left[ nadph \right]*\left[ pyr \right]}{K_{m}\left( nadph,pyr \right)} +\frac{\left[ pyr \right]*\left[ atp \right]}{K_{m}\left( pyr,atp \right)} +\frac{\left[ pyr \right]*\left[ accoa \right]}{K_{m}\left( pyr,accoa \right)} +\frac{\left[ nadph \right]*\left[ pyr \right]*\left[ accoa \right]}{K_{m}\left( nadph,pyr,accoa \right)}+\ldots\\ \ldots+\frac{\left[ nadph \right]*\left[ pyr \right]*\left[ atp \right]}{K_{m}\left( nadph,pyr,atp \right)}+\frac{\left[ co2 \right]*\left[ nadph \right]*\left[ pyr \right]}{K_{m}\left( co2,nadph,pyr \right)}+\frac{\left[ mal \right]*\left[ pyr \right]*\left[ accoa \right]}{K_{m}\left( mal,pyr,accoa \right)}+\ldots\\ \ldots+\frac{\left[ mal \right]*\left[ pyr \right]*\left[ atp \right]}{K_{m}\left( mal,pyr,atp \right)}+\frac{\left[ co2 \right]*\left[ nadph \right]*\left[ pyr \right]*\left[ accoa \right]}{K_{m}\left( co2,nadph,pyr,accoa \right)}+\frac{\left[ co2 \right]*\left[ nadph \right]*\left[ pyr \right]*\left[ atp \right]}{K_{m}\left( co2,nadph,pyr,atp \right)}+\ldots\\ \ldots+\frac{\left[ mal \right]*\left[ nadph \right]*\left[ pyr \right]*\left[ accoa \right]}{K_{m}\left( mal,nadph,pyr,accoa \right)}+\frac{\left[ mal \right]*\left[ nadph \right]*\left[ pyr \right]*\left[ atp \right]}{K_{m}\left( mal,nadph,pyr,atp \right)} \end{aligned} \right]}$ |
| **ME1** |
| $\frac{Vmax\left( f \right)*\left[ mal \right]-Vmax\left( r \right)*[nadh]*[pyr]*[co2]}{\left[ \begin{aligned} 1+\frac{\left[ mal \right]}{K_{m}\left( mal \right)}+\frac{\left[ co2 \right]}{K_{m}\left( co2 \right)}+\frac{\left[ nadh \right]}{K_{m}\left( nadh \right)}+\frac{\left[ pyr \right]*\left[ nadh \right]}{K_{m}\left( pyr,nadh \right)}+\frac{\left[ co2 \right]*\left[ nadh \right]}{K_{m}\left( co2,nadh \right)}+\frac{\left[ co2 \right]*\left[ pyr \right]}{K_{m}\left( co2,pyr \right)}+\ldots\\ \ldots+\frac{\left[ mal \right]*\left[ nadh \right]}{K_{m}\left( mal,nadh \right)}+\frac{\left[ mal \right]*\left[ pyr \right]}{K_{m}\left( mal,pyr \right)}+\frac{\left[ mal \right]*\left[ pyr \right]*\left[ nadh \right]}{K_{m}\left( mal,pyr,nadh \right)}+\frac{\left[ co2 \right]*\left[ pyr \right]*\left[ nadh \right]}{K_{m}\left( co2,pyr,nadh \right)} \end{aligned} \right]}$ |
| **PPC** |
| $\frac{Vmax\left( f \right)*\left[ pep \right]*\left[ co2 \right]-Vmax\left( r \right)*[oac]}{\left[ \begin{aligned} 1+\frac{\left[ pep \right]}{K_{m}\left( pep \right)}+\frac{\left[ co2 \right]}{K_{m}\left( co2 \right)}+\frac{\left[ oac \right]}{K_{m}\left( oac \right)}+kI\left( suc \right)*\left[ suc \right]+kI\left( cit \right)*\left[ cit \right]+kI\left( fum \right)*\left[ fum \right]+\ldots\\ \ldots+kI\left( cys \right)*\left[ cys \right]+kI\left( mal \right)*\left[ mal \right]+\frac{\left[ pep \right]*\left[ co2 \right]}{K_{m}\left( pep,co2 \right)}+\frac{\left[ pep \right]*\left[ mal \right]}{K_{m}\left( pep,mal \right)}+\frac{\left[ co2 \right]*\left[ oac \right]}{K_{m}\left( co2,oac \right)}+\ldots\\ \ldots+\frac{\left[ co2 \right]*\left[ suc \right]}{K_{m}\left( co2,suc \right)}+\frac{\left[ co2 \right]*\left[ cit \right]}{K_{m}\left( co2,cit \right)}+\frac{\left[ co2 \right]*\left[ fum \right]}{K_{m}\left( co2,fum \right)}+\frac{\left[ co2 \right]*\left[ cys \right]}{K_{m}\left( co2,cys \right)}+\frac{\left[ co2 \right]*\left[ mal \right]}{K_{m}\left( co2,mal \right)}+\frac{\left[ oac \right]*\left[ mal \right]}{K_{m}\left( oac,mal \right)} \end{aligned} \right]}$ |
| **PPCK** |
| $\frac{Vmax\left( f \right)*\left[ atp \right]*[oac]-Vmax\left( r \right)*[co2]*[pep]}{\left[ \begin{aligned} 1+\frac{\left[ atp \right]}{K_{m}\left( atp \right)}+\frac{\left[ oac \right]}{K_{m}\left( oac \right)}+\frac{\left[ co2 \right]}{K_{m}\left( co2 \right)}+\frac{\left[ pep \right]}{K_{m}\left( pep \right)}+kI\left( f6p \right)*\left[ f6p \right]+kI\left( fdp \right)*\left[ fdp \right]+\ldots\\ \ldots+kI\left( atp \right)*\left[ atp \right]+kI\left( pep \right)*\left[ pep \right]+\frac{\left[ atp \right]*\left[ oac \right]}{K_{m}\left( atp,oac \right)}+\frac{\left[ atp \right]*\left[ pep \right]}{K_{m}\left( atp,pep \right)}+\frac{\left[ oac \right]*\left[ nadh \right]}{K_{m}\left( oac,nadh \right)}+\ldots\\ \ldots+\frac{\left[ oac \right]*\left[ dhap \right]}{K_{m}\left( oac,dhap \right)}+\frac{\left[ oac \right]*\left[ atp \right]}{K_{m}\left( oac,atp \right)}+\frac{\left[ oac \right]*\left[ pep \right]}{K_{m}\left( oac,pep \right)}+\frac{\left[ oac \right]*\left[ co2 \right]}{K_{m}\left( oac,co2 \right)}+\frac{\left[ oac \right]*\left[ f6p \right]}{K_{m}\left( oac,f6p \right)}+\ldots\\ \ldots+\frac{\left[ oac \right]*\left[ fdp \right]}{K_{m}\left( oac,fdp \right)}+\frac{\left[ oac \right]*\left[ atp \right]}{K_{m}\left( oac,atp \right)}+\frac{\left[ oac \right]*\left[ pep \right]}{K_{m}\left( oac,pep \right)}+\frac{\left[ co2 \right]*\left[ pep \right]}{K_{m}\left( co2,pep \right)}+\frac{\left[ pep \right]*\left[ f6p \right]}{K_{m}\left( pep,f6p \right)}+\ldots\\ \ldots+\frac{\left[ pep \right]*\left[ fdp \right]}{K_{m}\left( pep,fdp \right)}+\frac{\left[ pep \right]*\left[ atp \right]}{K_{m}\left( pep,atp \right)}+\frac{\left[ pep \right]^{2}}{K_{m}\left( pep,pep \right)}+\frac{\left[ atp \right]*\left[ oac \right]*\left[ pep \right]}{K_{m}\left( atp,oac,pep \right)}+\ldots\\ \ldots+\frac{\left[ atp \right]*\left[ pep \right]*\left[ nadh \right]}{K_{m}\left( atp,pep,nadh \right)}+\frac{\left[ atp \right]*\left[ pep \right]*\left[ dhap \right]}{K_{m}\left( atp,pep,dhap \right)}+\frac{\left[ atp \right]^{2}*\left[ pep \right]}{K_{m}\left( atp,pep,atp \right)}+\frac{\left[ atp \right]*\left[ pep \right]^{2}}{K_{m}\left( atp,pep,pep \right)}+\ldots\\ \ldots+\frac{\left[ oac \right]*\left[ co2 \right]*\left[ pep \right]}{K_{m}\left( oac,co2,pep \right)}+\frac{\left[ co2 \right]*\left[ pep \right]*\left[ nadh \right]}{K_{m}\left( co2,pep,nadh \right)}+\frac{\left[ co2 \right]*\left[ pep \right]*\left[ dhap \right]}{K_{m}\left( co2,pep,dhap \right)}+\ldots\\ \ldots+\frac{\left[ co2 \right]*\left[ pep \right]*\left[ atp \right]}{K_{m}\left( co2,pep,atp \right)}+\frac{\left[ co2 \right]*\left[ pep \right]^{2}}{K_{m}\left( co2,pep,pep \right)} \end{aligned} \right]}$ |
| **PTAr/ACKr** |
| $\frac{Vmax\left( f \right)*\left[ accoa \right]-Vmax\left( r \right)*[ac]*[atp]}{\left[ \begin{aligned} 1+\frac{\left[ accoa \right]}{K_{m}\left( accoa \right)}+\frac{\left[ ac \right]}{K_{m}\left( ac \right)}+\frac{\left[ atp \right]}{K_{m}\left( atp \right)}+\frac{\left[ accoa \right]*\left[ atp \right]}{K_{m}\left( accoa,atp \right)}+\frac{\left[ ac \right]*\left[ atp \right]}{K_{m}\left( ac,atp \right)} \end{aligned} \right]}$ |
| **GLUDy** |
| $\frac{Vmax\left( f \right)*\left[ nadph \right]*\left[ akg \right]*\left[ nh3 \right]-Vmax\left( r \right)*[glu]}{\left[ \begin{aligned} 1+\frac{\left[ nadph \right]}{K_{m}\left( nadph \right)}+\frac{\left[ nh3 \right]}{K_{m}\left( nh3 \right)}+\frac{\left[ glu \right]}{K_{m}\left( glu \right)}+\frac{\left[ nh3 \right]*\left[ glu \right]}{K_{m}\left( nh3,glu \right)}+\frac{\left[ akg \right]*\left[ glu \right]}{K_{m}\left( akg,glu \right)}+\frac{\left[ akg \right]*\left[ nh3 \right]}{K_{m}\left( akg,nh3 \right)}+\ldots\\ \ldots+\frac{\left[ nadph \right]*\left[ nh3 \right]}{K_{m}\left( nadph,nh3 \right)}+\frac{\left[ nadph \right]*\left[ akg \right]}{K_{m}\left( nadph,akg \right)}+\frac{\left[ nadph \right]*\left[ akg \right]*\left[ nh3 \right]}{K_{m}\left( nadph,akg,nh3 \right)}+\frac{\left[ akg \right]*\left[ nh3 \right]*\left[ glu \right]}{K_{m}\left( akg,nh3,glu \right)} \end{aligned} \right]}$ |
| **GLNS** |
| $\frac{Vmax\left( f \right)*\left[ atp \right]*\left[ nh3 \right]*\left[ glu \right]-Vmax\left( r \right)*[gln]}{\left[ \begin{aligned} 1+\frac{\left[ atp \right]}{K_{m}\left( atp \right)}+\frac{\left[ glu \right]}{K_{m}\left( glu \right)}+\frac{\left[ gln \right]}{K_{m}\left( gln \right)}+\frac{\left[ glu \right]*\left[ gln \right]}{K_{m}\left( glu,gln \right)}+\frac{\left[ nh3 \right]*\left[ gln \right]}{K_{m}\left( nh3,gln \right)}+\frac{\left[ nh3 \right]*\left[ glu \right]}{K_{m}\left( nh3,glu \right)}+\ldots\\ \ldots+\frac{\left[ atp \right]*\left[ glu \right]}{K_{m}\left( atp,glu \right)}+\frac{\left[ atp \right]*\left[ nh3 \right]}{K_{m}\left( atp,nh3 \right)}+\frac{\left[ atp \right]*\left[ nh3 \right]*\left[ glu \right]}{K_{m}\left( atp,nh3,gly \right)}+\frac{\left[ nh3 \right]*\left[ glu \right]*\left[ gln \right]}{K_{m}\left( nh3,glu,gln \right)} \end{aligned} \right]}$ |
| **GHMT2r** |
| $\frac{Vmax\left( f \right)*\left[ ser \right]-Vmax\left( r \right)*[gly]*[meethf]}{\left[ \begin{aligned} 1+\frac{\left[ ser \right]}{K_{m}\left( ser \right)}+\frac{\left[ meethf \right]}{K_{m}\left( meethf \right)}+\frac{\left[ gly \right]}{K_{m}\left( gly \right)}+\frac{\left[ ser \right]*\left[ gly \right]}{K_{m}\left( ser,gly \right)}+\frac{\left[ meethf \right]*\left[ gly \right]}{K_{m}\left( meethf,gly \right)} \end{aligned} \right]}$ |
| **GLYCL** |
| $\frac{Vmax\left( f \right)*\left[ gly \right]-Vmax\left( r \right)*[nadh]*[co2]*[nh3]*[meethf]}{\left[ \begin{aligned} 1+\frac{\left[ gly \right]}{K_{m}\left( gly \right)}+\frac{\left[ meethf \right]}{K_{m}\left( meethf \right)}+\frac{\left[ madh \right]}{K_{m}\left( nadh \right)}+\frac{\left[ co2 \right]*\left[ nadh \right]}{K_{m}\left( co2,nadh \right)}+\frac{\left[ meethf \right]*\left[ nadh \right]}{K_{m}\left( meethf,nadh \right)}+\ldots\\ \ldots+\frac{\left[ meethf \right]*\left[ nh3 \right]}{K_{m}\left( meethf,nh3 \right)}+\frac{\left[ gly \right]*\left[ nadh \right]}{K_{m}\left( gly,nadh \right)}+\frac{\left[ gly \right]*\left[ co2 \right]}{K_{m}\left( gly,co2 \right)}+\frac{\left[ gly \right]*\left[ nh3 \right]}{K_{m}\left( gly,nh3 \right)}+\frac{\left[ nh3 \right]*\left[ co2 \right]*\left[ nadh \right]}{K_{m}\left( nh3,co2,nadh \right)}+\ldots\\ \ldots+\frac{\left[ meethf \right]*\left[ co2 \right]*\left[ nadh \right]}{K_{m}\left( meethf,co2,nadh \right)}+\frac{\left[ meethf \right]*\left[ nh3 \right]*\left[ nadh \right]}{K_{m}\left( meethf,nh3,nadh \right)}+\frac{\left[ meethf \right]*\left[ nh3 \right]*\left[ co2 \right]}{K_{m}\left( meethf,nh3,co2 \right)}+\ldots\\ \ldots+\frac{\left[ gly \right]*\left[ co2 \right]*\left[ nadh \right]}{K_{m}\left( gly,co2,nadh \right)}+\frac{\left[ gly \right]*\left[ nh3 \right]*\left[ co2 \right]}{K_{m}\left( gly,nh3,co2 \right)}+\frac{\left[ gly \right]*\left[ nh3 \right]*\left[ co2 \right]*\left[ nadh \right]}{K_{m}\left( gly,nh3,co2,nadh \right)}+\ldots\\ \ldots+\frac{\left[ meethf \right]*\left[ nh3 \right]*\left[ co2 \right]*\left[ nadh \right]}{K_{m}\left( meethf,nh3,co2,nadh \right)} \end{aligned} \right]}$ |
| **SERD-L** |
| $\frac{Vmax\left( f \right)*\left[ ser \right]-Vmax\left( r \right)*[pyr]*[nh3]}{\left[ 1+\frac{\left[ ser \right]}{K_{m}\left( ser \right)}+\frac{\left[ nh3 \right]}{K_{m}\left( nh3 \right)}+\frac{\left[ pyr \right]}{K_{m}\left( pyr \right)}+\frac{\left[ ser \right]*\left[ pyr \right]}{K_{m}\left( ser,pyr \right)}+\frac{\left[ nh3 \right]*\left[ pyr \right]}{K_{m}\left( nh3,pyr \right)} \right]}$ |
| **METHFR2** |
| $\frac{Vmax\left( f \right)*\left[ nadh \right]*\left[ meethf \right]-Vmax\left( r \right)*[methf]}{\left[ 1+\frac{\left[ nadh \right]}{K_{m}\left( nadh \right)}+\frac{\left[ meethf \right]}{K_{m}\left( meethf \right)}+\frac{\left[ methf \right]}{K_{m}\left( methf \right)}+\frac{\left[ nadh \right]*\left[ meethf \right]}{K_{m}\left( nadh,meethf \right)}+\frac{\left[ meethf \right]*\left[ methf \right]}{K_{m}\left( meethf,methf \right)} \right]}$ |
| **MTHFD** |
| $\frac{Vmax\left( f \right)*\left[ meethf \right]-Vmax\left( r \right)*[nadph]*\left[ fthf \right]}{\left[ 1+\frac{\left[ meethf \right]}{K_{m}\left( meethf \right)}+\frac{\left[ fthf \right]}{K_{m}\left( fthf \right)}+\frac{\left[ nadph \right]}{K_{m}\left( nadph \right)}+\frac{\left[ meethf \right]*\left[ nadph \right]}{K_{m}\left( meethf,nadph \right)}+\frac{\left[ fthf \right]*\left[ nadph \right]}{K_{m}\left( fthf,nadph \right)} \right]}$ |
| **NADTRHD** |
| $\frac{Vmax\left( f \right)*\left[ nadh \right]-Vmax\left( r \right)*[nadph]}{\left[ 1+\frac{\left[ nadh \right]}{K_{m}\left( nadh \right)}+\frac{\left[ nadph \right]}{K_{m}\left( nadph \right)} \right]}$ |
